# Species composition of arbuscular mycorrhizal communities changes with elevation in the Andes of South Ecuador

**DOI:** 10.1371/journal.pone.0221091

**Published:** 2019-08-16

**Authors:** Ingeborg Haug, Sabrina Setaro, Juan Pablo Suárez

**Affiliations:** 1 Evolutionary Ecology of Plants, Eberhard-Karls-University, Tübingen, Germany; 2 Department of Biology, Wake Forest University, Winston-Salem, North Carolina, United States of America; 3 Departamento de Ciencias Biológicas, Universidad Técnica Particular de Loja, Loja, Ecuador; Friedrich Schiller University, GERMANY

## Abstract

Arbuscular mycorrhizal fungi (AMF) are the most prominent mycobionts of plants in the tropics, yet little is known about their diversity, species compositions and factors driving AMF distribution patterns. To investigate whether elevation and associated vegetation type affect species composition, we sampled 646 mycorrhizal samples in locations between 1000 and 4000 m above sea level (masl) in the South of Ecuador. We estimated diversity, distribution and species compositions of AMF by cloning and Sanger sequencing the 18S rDNA (the section between AML1 and AML2) and subsequent derivation of fungal OTUs based on 99% sequence similarity. In addition, we analyzed the phylogenetic structure of the sites by computing the mean pairwise distance (MPD) and the mean nearest taxon difference (MNTD) for each elevation level. It revealed that AMF species compositions at 1000 and 2000 masl differ from 3000 and 4000 masl. Lower elevations (1000 and 2000 masl) were dominated by members of Glomeraceae, whereas Acaulosporaceae were more abundant in higher elevations (3000 and 4000 masl). Ordination of OTUs with respect to study sites revealed a correlation to elevation with a continuous turnover of species from lower to higher elevations. Most of the abundant OTUs are not endemic to South Ecuador. We also found a high proportion of rare OTUs at all elevations: 79–85% of OTUs occurred in less than 5% of the samples. Phylogenetic community analysis indicated clustering and evenness for most elevation levels indicating that both, stochastic processes and habitat filtering are driving factors of AMF community compositions.

## Introduction

Arbuscular mycorrhizal fungi (AMF) are the most prominent mycobionts of plants in the Neotropics [1, 2]. Arbuscular mycorrhizal fungi belong to the phylum Glomeromycotina, and are crucial for providing minerals to the vast majority of plants including nearly all tree species in nutrient-poor environments [1, 2]. Thus, knowledge of this mutualistic plant-fungus association is indispensable for understanding the biology and ecology of tropical ecosystems. Surveys on birds, mammals, soil arthropods or aquatic animals in tropical forests have been conducted since centuries but surveys of tropical fungi—especially AMF—long been sparse, have been increasing during the last decade [3–7]. The main reason why AMF have not been studied until recently is that they are cryptic microorganisms that live exclusively in the roots of their plant hosts or within the soil as dormant spores or mycelium. These fungi only exist in an asexual state and are notoriously difficult to culture. The elusive nature of AMF makes it not only difficult to understand species boundaries and diversity estimation but also hinders the understanding of AMF community composition and their driving factors. Some of these factors have been identified, such as environment, distance-based effects, stochastic events and phylogenetic distance [8].

Most of the studies on the effect of elevation on AMF were done in alpine grasslands [9–17]. AMF community composition was identified with spores or through PCR-based analyses of AMF hyphae from soil or within mycorrhizas. Lugo *et al*. [10], Gai *et al*. [11]and Kotilinek *et al*. [17] showed species richness decreasing with increasing elevation, whereas in the study of Yang *et al*. [15] elevation did not significantly affect AMF OTU richness. Egan *et al*. [16] found lower phylogenetic diversity in alpine AMF communities relative to lower elevation communities. The elevation gradients differed in magnitude, the methods were varying, but overall there is the consensus that AMF community composition is affected by elevation and most studies find a decreasing richness of AMF in higher elevations.

In neotropical forests, studies were done by Geml *et al*. [18], Bonfim *et al*. [19], Looby *et al*. [20] and Camenzind *et al*. [21]. In the Andean Yungas Forests, Geml *et al*. [18] studied the community composition of fungi in three elevation belts (400 to 3000 masl) based on Ion Torrent sequencing of the ITS2 rDNA from soil samples. Geml and coauthors [18] found a strong community turnover across elevations. Glomeromycota comprised 2.91% of the entire soil fungal community—equaling 409 OTUs, of which 321 OTUs were Glomerales. AMF richness was negatively correlated with elevation. This contrasts the results of Bonfim *et al*. [19], who studied AMF communities on the basis of morphological spore analyses and molecular analyses of mycorrhizas (18S region: primer NS31 –AM1) in a Brazilian Atlantic forest at 80, 600, and 1000 masl. Bonfim *et al*. [19] found the highest AMF species richness and diversity to be in high elevations (1000 masl). *Glomus* and *Acaulospora* spores were the dominant morphotypes of all 58 identified AMF spore types, and Glomeraceae the only family found in all fourteen sequence groups [19]. In Costa Rica, Looby *et al*. [20] studied community compositions of fungi in soil samples along an elevation gradient (1305 to 1850 masl) in the tropical montane cloud forest sequencing ITS2 rDNA with Illumina Miseq. They observed decreasing AMF richness with higher elevation but only during the dry season, not the wet season. Camenzind *et al*. [21] conducted a study in a tropical montane forest in Ecuador along a large gradient spanning 2000m (1000 masl—3000 masl). They quantified AMF root colonization and extraradical AMF biomass; both significantly increased with elevation [21].

These four studies provided a first insight into AMF communities of neotropical forests but were conducted with different methods and revealed different results. There is a large range of AMF species/OTU counts among these studies. Next generation sequencing reveals OTU counts that are much higher than those yielded by morphotyping or Sanger sequencing with cloning. However, all these studies indicate that Glomeraceae are dominating. In order to better understand how neotropical AMF community structure is driven across elevation belts, more research is needed. Here we present results from our studies of AMF communities in the tropical Andes of Southern Ecuador. The Southern Ecuadorian Andes are a promising and suitable research area because they have a breadth of vegetation types, show a broad elevation range and are among the world’s hotspots of biodiversity [22]. This makes it possible to study the influence of elevation on biodiversity and community structure of AMF in order to reveal more about the ecosystems’ ecology. The goal of this study was to investigate distribution patterns of arbuscular mycorrhizal fungi in neotropical forests, specifically of Southern Ecuador, and to get an insight into the structure of these communities. We focused on four elevation belts with different types of vegetation: evergreen premontane forest (≈1000 masl), evergreen lower montane forest (≈2000 masl), upper montane forest (≈3000 masl) and grass páramo with patchy *Polylepis* forest (≈4000 masl). In this area, there is not only a pronounced turnover of tree species and families but also changes in structural features of the forest canopy [3, 23, 24]. Community analysis of AMF did not show a clear picture about the influence of elevation on community structure, as outlined above. In addition, our own research in the tropical mountain rain forest showed overlap of AMF OTUs among different sites and forest types [4, 25]. However, the previously studied sites were close in proximity and had a limited elevational gradient (1800 – 2200m). With this study, we increased the gradient by adding samples from 1000, 3000 and 4000 masl of elevation. We wanted to test the hypothesis that there is a turnover of AMF communities from lower to higher elevations in the Andes of South Ecuador.

## Materials and methods

### The study region

**Fig 1:**
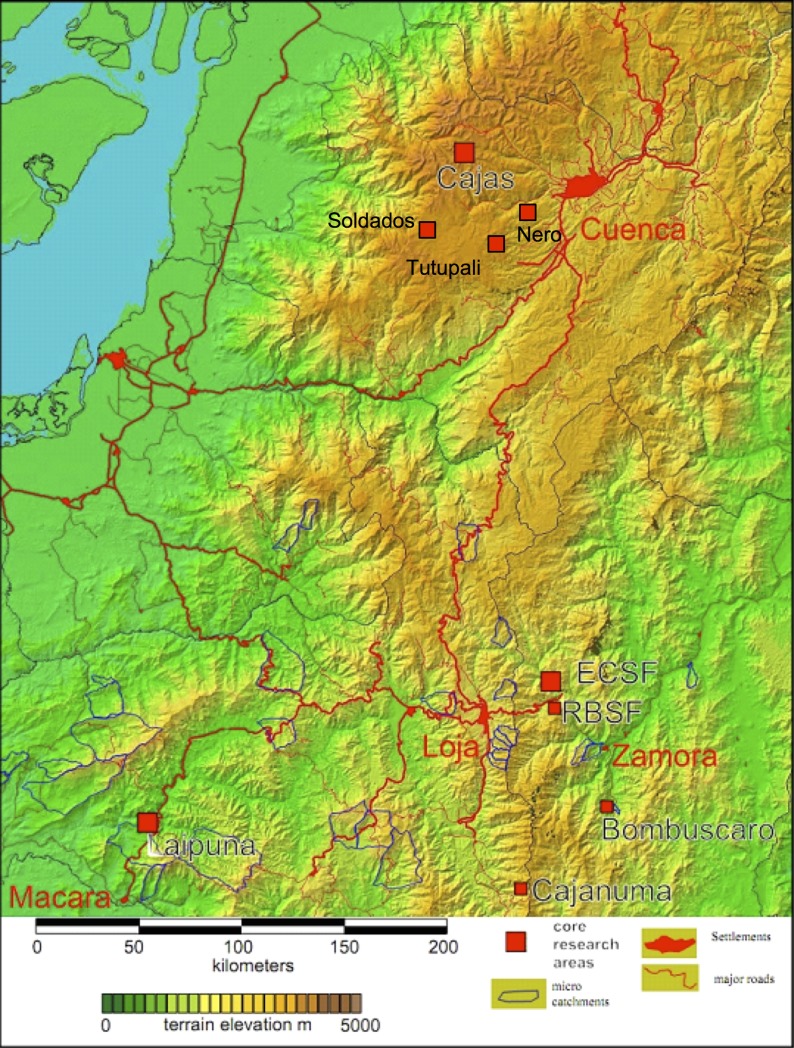
Map of the study region in Southern Ecuador. Location of the six study areas: Bombuscaro, Reserva Biologíca San Francisco (RBSF), Cajanuma, Nero, Soldados, Toreadora. http://vhrz669.hrz.uni-marburg.de/tmf_respect/data_pre.do?citid=1745.

Samples were taken at four elevation belts in Southern Ecuador: 1000 masl, 2000 masl, 3000 masl and 4000 masl (Fig 1, Table 1). The 1000 m sites are located in the Bombuscaro area in Parque Nacional Podocarpus (4°11’S, 78°96’W); the 2000 m sites in the Reserva Biológica San Francisco (RBSF; 3°58’S, 79°04’W) on the eastern slope of the Cordillera El Consuelo, Zamora-Chinchipe Province; the 3000 m sites in Cajanuma area (4°12'S, 79°17'W) in Parque Nacional Podocarpus and in the Nero area (2°95’S, 79°10’W) at Parque Nacional Cajas; and the 4000 m sites in Tutupali (3°03’S, 79°15’W), Soldados (2°98’S, 79°31’W) and the Toreadora area (2°47'S, 79°11'W) at Parque Nacional Cajas (Fig 1). The vegetation at 1000 masl consists of evergreen premontane rainforest, whereas the forest at 2000 masl is characterized by evergreen lower montane forest [23]. Upper montane forest occurs at the 3000 m sites; *Polylepis* forest and grass páramo can be found at the 4000 m sites. Details are summarized in Table 1 (data from [23, 24, 26–29]).

**Table 1:** Site characteristics for the four elevation levels.

Elevation level	1000 m site	2000 m site	3000 m site	4000 m site
Sites	PNP—Bombuscaro	RBSF	PNP—Cajanuma	Cajas NP—Toreadora
Elevation	1050 masl	1890 masl	3060 masl	3930 masl
Vegetation	Evergreen premontane forest	Evergreen lower montane forest	Upper montane forest, Shrub Páramo	Grass Páramo, *Polylepis* forest
LAI (= leaf area index)	5.1 ± 0.1	3.9 ± 0.2	2.9 ± 0.3	3.30–3.51
Annual mean air temperature	19.4°C	15.7°C	9.4°C	5.4°C
Annual rainfall	c. 2230 mm	c. 1950 mm	c. 4500 mm	c. 876 mm
Soil type (FAO)	Dystric Cambisol	Stagnic Cambisol	Stagnic Histosol	Histosol, Andosol
Thickness of organic layer	0 cm	10–30 cm	10–40 cm	10–40 cm
Organic layer pH-H2O		3.9	3.9	4.2–4.8
Mineral soil layer (top 5cm) pH-H2O	4.6	3.8	4.7	4.5–5.7
N (mg/cm3 soil), mean ± standard error	2.86 ± 0.44	3.13 ± 0.11	1.77 ± 0.14	
P (mg/cm3 soil), mean ± standard error	0.165 ± 0.023	0.088 ± 0.006	0.055 ± 0.007	
Stand height	20-25m, up to 40 m	18–22 m	8–10 m	
Abundant tree families	Fabaceae, Melastomataceae, Moraceae, Myristicaceae, Rubiaceae, Sapotaceae	Euphorbiaceae, Lauraceae, Melastomataceae, Rubiaceae	Aquifoliaceae, Clusiaceae, Cunoniaceae, Lauraceae, Melastomataceae	
Common tree species	*Pouteria porta*, *Clarisia racemosa*	*Graffenrieda emarginata*, *Myrica* sp.	*Weinmannia elliptica*, *W*. *loxensis*	*Polylepis* sp., *Gynoxys* sp.

### Study sites and sampling

At each elevation belt, we sampled three to four sites (S1 Table). We collected mycorrhizas directly from the soil without regarding the identity of the plant partner. This strategy allows for a more ample and random sample but inhibits studying the effects of plant species on AMF partners. We opted for this strategy, because association of AMF to specific plant partners seem to be weak [4, 9, 30] and sampling the same host plant species in different elevations is not feasible because of high turnover of plant communities with elevation [23, 31]. Mycorrhizas were sampled by collecting fine roots from the organic layer or the topsoil (2–10 cm soil depth) where most of the fine roots are located. The organic layer was loosened with a rake and sections of fine-root systems were sampled in plastic bags. The sampled root systems were cleaned under tap water on the same day. Fine root systems were sorted manually and dried out root systems were discarded because DNA extraction works best with fresh vital material [32]. Each fine-root system was placed in a separate PCR tube to ensure that each mycorrhizal sample is from a single host plant. Mycorrhizas were dried at about 50°C for 24 hours by placing open sample tubes on an electric dryer. After the drying step, silica gel was added and the tubes were closed for long-term storage.

In total, we successfully worked with 646 root samples: 211 at 1000 masl, 184 at 2000 masl, 128 at 3000 masl, and 123 at 4000 masl (see details in S1 Table).

### DNA isolation

Approximately 5–10 root segments, with a length of 2 cm were ground using a carbid ball and a mixer mill (2x1. 30 min, frequency 30/sec). Total DNA was isolated with the innuPREP Plant DNA Kit (Analytik Jena, Germany) and re-suspended in a final volume of 100 μl of elution buffer.

### PCR, cloning and Sanger sequencing

Part of the SSU region of the nuclear ribosomal rDNA repeat was amplified by PCR using a volume of 0.5 μl of the DNA template. A nested PCR approach was applied, amplifying the larger outer fragment with the primer combination NS1/NS4 [33, 34] and the smaller, inner fragment with the primer pair AML1/AML2 [34] for details see [4]. Amplified PCR products were cloned with the Invitrogen TA Cloning Kit (Life Technologies) following the manufacturer’s instructions but using a third of the indicated volumes. Inserts were re-amplified from clones with primers M13F/M13R by picking eight bacterial clones with a toothpick and the toothpick-sticking bacteria were transferred by stirring in a PCR reaction mixture. Inserts were digested with restriction enzymes HinfI or AfaI. Digested products were examined on a 0.7% agarose gel. Two clones of the same RFLP pattern were cleaned and sequenced by GATC Biotech (Konstanz, Germany). Whenever the two sequences were not identical, two additional clones of the same RFLP pattern were sequenced. In case analysis revealed more than three different Glomeromycota sequences per PCR product, an additional eight clones were picked and analyzed. About 5% of the sequences were non-Glomeromycotina (plants, ascomycetes, basidiomycetes, animals).

Sequences were edited with Sequencher (Version 4.9, Gene Codes, Ann Arbor, Michigan), and a BLAST search was performed against the nucleotide sequence database (NCBI) and against the MaarjAM database. When several inserts of a cloned PCR product belonged to the same OTU, only one sequence was included in the final data set.

The final dataset (S1 and S2 Tables) consisted of 1209 Glomeromycotan sequences: 995 sequences generated in this study and 214 sequences from former studies done on the same study sites [4, 5, 25]. Newly generated sequences (995 sequences) were deposited in GenBank (see accession numbers in S2 Table).

### OTU delimitation and estimation of species richness

All sequences were ~800 bp long and comprised part of the 18S. Sequences were aligned with MAFFT version 7 (https://mafft.cbrc.jp/alignment/server/, [35]; MAFFT-L-INS-i) and a distance matrix based on p-distances was created with PAUP for OTU delimitation.

Subsequently, Operational Taxonomic Units (OTUs) were defined as surrogates for species based on sequence similarity with OPTSIL [36]. We used intermediate linkage clustering and a cut-off value of 99% sequence similarity. This approach splits OTUs if 50% or more of their containing sequences are less than 99% similar to another OTU (for details see [36]). Fifteen OTUs contained only one sequence and were not included in subsequent analyses. We chose a similarity threshold of 99% instead of the more popular 97%, because we obtained fewer OTUs that spanned sequences from different genera (e.g. sequences from *Gigaspora* and *Scutellospora* (see S3 Table).

For all remaining OTUs, we calculated a sample-based rarefaction accumulation curve with 95% confidence intervals, estimated the total OTU richness with Chao2 and Jackknife2 and calculated similarity indices using the software *Estimate*S 9.1.0 [37].

### AMF community analyses

To explore differences in AMF community compositions among sampling sites, we carried out a non-metric multidimensional scaling (NMDS) ordination with metaMDS from the R package VEGAN [38]. Our data set contained 16 sites and 115 OTUs. The underlying matrix was a Bray-Curtis dissimilarity matrix derived from OTU abundances of each site. The Bray-Curtis dissimilarity index is often used for environmental data and focuses on abundant species between sites. The metaMDS function of the VEGAN package in R [38] allows for calculating the NMDS ordination multiple times with random starts in order to find a stable result (https://www.rdocumentation.org/packages/vegan/versions/2.4-2/topics/metaMDS). We used a maximum number of 1000 iterations in a 3-dimensional space.

In addition, we performed PERMANOVA with the same distance matrix as for the NMDS ordination using elevation levels as variable. PERMANOVA was carried out with the ADONIS function in VEGAN [38] and 999 permutations.

We also ran pairwise PERMANOVA (pairwise.adonis, R) to test for OTU similarity between each site using Holm’s correction to control for multiple comparisons.

To check for potential sampling errors, we tested whether OTU similarity is significantly correlated with sample size using a ranked mantel test with the Spearman method in VEGAN [38]. We chose the Spearman method to calculate a ranked mantel test to keep the method consistent with our NMDS analysis. Randomization was computed with 999 runs. The first matrix was the same distance matrix as for NMDS ordination and PERMANOVA and the second matrix was an euclidean distance matrix based on samples taken for each sample site.

### Phylogenetic community analyses

The same alignment as for OTU delimitation (see paragraph above) was used to reconstruct a phylogeny. We computed a Maximum Likelihood tree (S1 Fig) RAxML v.8.2.11 [39] with 1000 rapid bootstrap inferences and a thorough ML optimized under the GAMMA model of rate heterogeneity. The Maximum Likelihood tree was pruned to contain only one sequence per OTU, which was picked randomly (S2 Table). This phylogeny was used to assess phylogenetic community structures of AMF for each elevation level using two measures: the standardized effect size of the mean pairwise distance (MPD) and the standardized effect size of the mean nearest taxon index (MNTI) both calculated with the R package PICANTE [40]. The MPD and the MNTI are equivalent to -1 times of the Nearest Relative Index (NRI) and the Nearest Taxon Index (NTI), respectively.

We tested four different null models making different speciation assumptions with 1000 iterations each. All of these null models were included in PICANTE:

1. Randomization of taxon labels in the distance matrix (parameter “taxa.labels”). This null model shuffles phylogenetic relationships among OTUs assuming phylogenetic evenness.
2. Randomization of abundances within samples, maintaining species richness of elevation levels (parameter “richness”).
3. Randomization by drawing OTUs across all samples in the distance matrix with equal probability (parameter “phylogeny.pool”). This null model assumes equal probability of AMF community membership among sites.
4. Randomization with trial-and-swap algorithm maintaining OTU frequency in elevation levels and species richness (parameter “trialswap”).

## Results and discussion

### Turnover of AMF communities with increasing elevation

**Fig 2:**
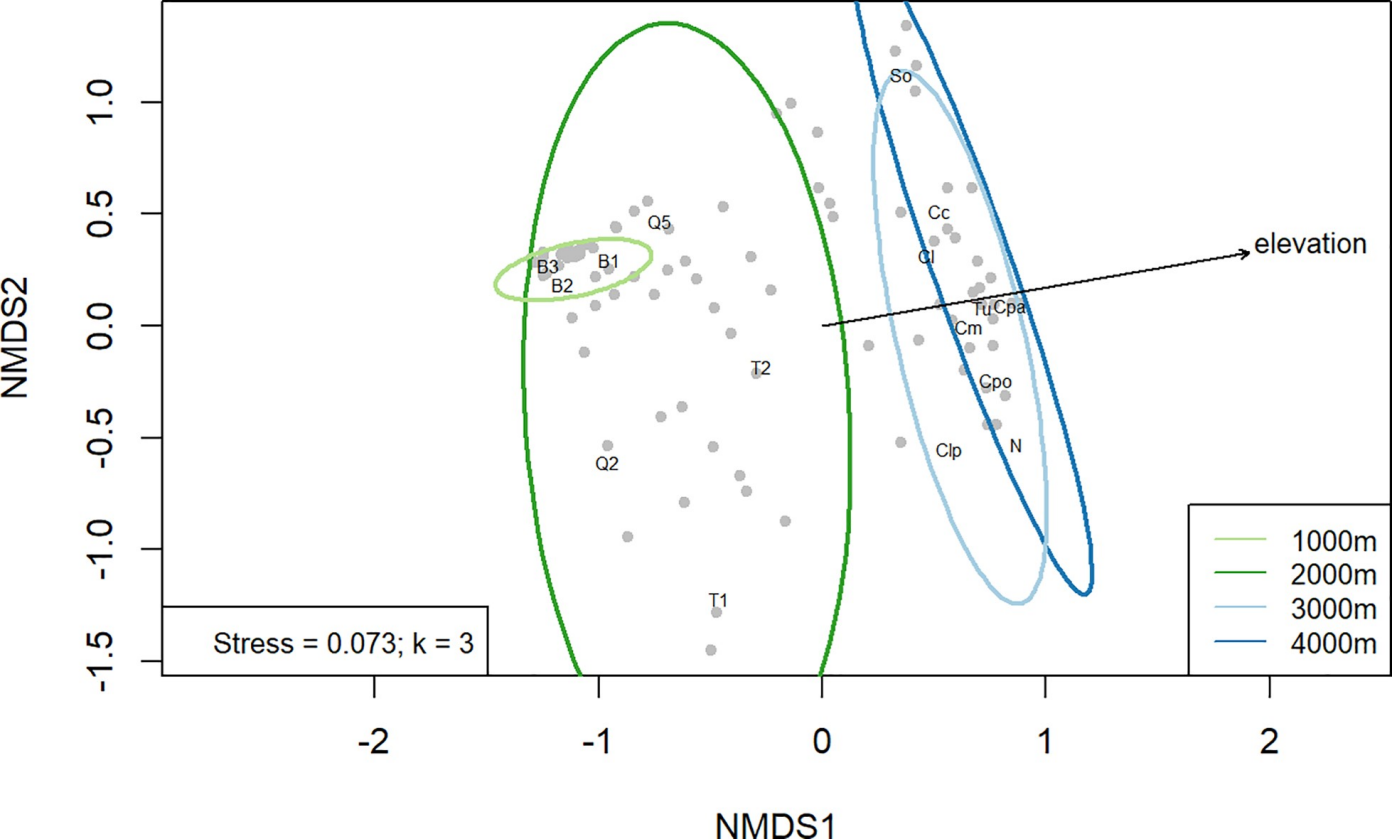
NMDS plot for AM fungal communities. NMDS ordination showing turnover of species composition along elevational gradient. Species scores are shown as gray dots and site scores as black labels. The ellipses around data points show the 95% confidence interval for the 1000 m (light green), 2000 m (green), 3000 m (light blue) and 4000 m (blue) sites. The 1000 m and 2000 m confidence ellipses were not overlapping with the 3000 m and 4000 m ellipses, indicating significant turnover of AMF communities from 2000 m to 3000 m. Overall effect of elevation on AMF communities is supported by PERMANOVA (F = 2.6488, p = 0.001). Overall stress of the ordination is 0.073 with 3 dimensions (k = 3).

**Fig 3:**
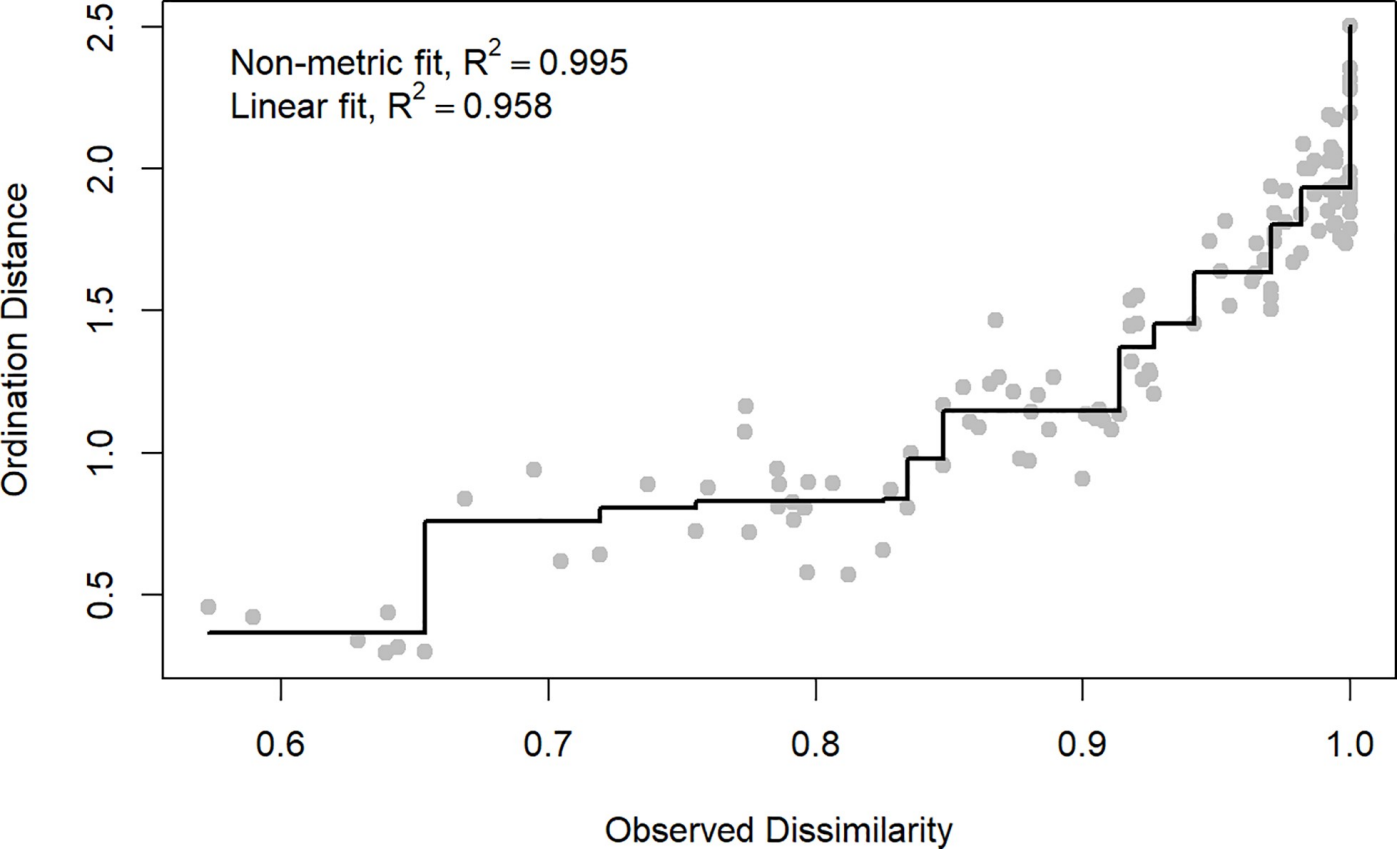
Shepard plot of the NMDS ordination. Shepard plot showing the relationship of the observed dissimilarity with the calculated ordination distance as a measure of goodness of fit.

The four elevation levels (1000, 2000, 3000 and 4000 masl) had distinct AMF communities (Fig 2) with many OTUs (50%) occurring only at one elevation level. The NMDS analysis and PERMANOVA (F = 2.6488, p = 0.001) indicated a significant correlation between elevation and community composition of AMF (Figs 2 and 3). Mantel statistics indicated no correlation between sample size and OTU similarity among the sites (r = 0.08828, p = 0.184).

The effect of elevation on AMF communities corroborate the findings of Gai *et al*. [11], who studied AMF spore communities along 1990–4648 masl. It is also in line with findings of Li *et al*. [13] and Yang *et al*. [15], who investigated AMF in soil samples along a gradient in an elevation of 4149 to 5033 masl and 3200–3800 masl, respectively. Also Kotilinek *et al*. [17] found a significant effect of elevation (3400 to 6150 masl) on AMF community composition in plant roots; and Li *et al*. [9] observed a clear separation of AMF communities among four elevations (3105, 3877, 4306, 4556 masl).

Ordination results showed an overlap in OTU composition between 1000m and 2000m as well as between 3000m and 4000m (Fig 2), with little overlap between 2000m and 3000m. This is supported by the results of pairwise PERMANOVA only showing significant difference in AMF community for 2000 masl and 3000 masl elevation (p = 0.008 without Holm correction, p = 0.048 after Holm correction, Table 2). Overlap of OTUs in the neighboring elevation levels of 1000 masl and 2000 masl was 23%, and between 3000 masl and 4000 masl it was 32% (S2 Fig, S4 Table). Whereas the most distant elevation levels 1000 masl and 4000 masl only had five OTUs in common and all similarity indices for these sites were low (S5 Table). However, a distinct AMF community between the 1000 masl and 4000 masl m level was not supported statistically with PERMANOVA after Holm correction (p = 0.128, Table 2).

**Table 2:** P-values of pairwise PERMANOVA for each elevation level.

Comparison	1000	2000	3000	4000
1000	-	0.056	0.021	0.038
2000	**0.148**	-	0.006	0.037
3000	**0.105**	0.036*	-	0.479
4000	**0.148**	**0.148**	**0.479**	-

P-values after Holm Correction are shown in bold. Uncorrected P-values are in normal font.

* = p<0.05.

The amount of shared OTUs in neighboring elevations would explain why studies investigating the effect of elevation in smaller gradients did not find distinct AMF communities (~ 800 m—[41]; ~550 m—[16]; ~900 m—[13]).

All elevation levels had a similar estimated diversity, but OTU accumulation curves indicated (S3 Fig) that additional sampling may have resulted in the detection of more additional OTUs at the 3000 and 4000 masl level.

Similar diversity among elevation levels was also found in Qinghai-Tibet Plateau by Yang et al. [15]. Declining richness with elevation was found in studies analyzing AMF spores [10, 11] and in very high elevations above 4000 masl [17]. Studies showed a higher spore formation under higher temperatures [42], which explains declining richness of spores with elevation.

### Reverse dominance patterns of *Glomus* and *Acaulospora* in higher elevations

**Fig 4:**
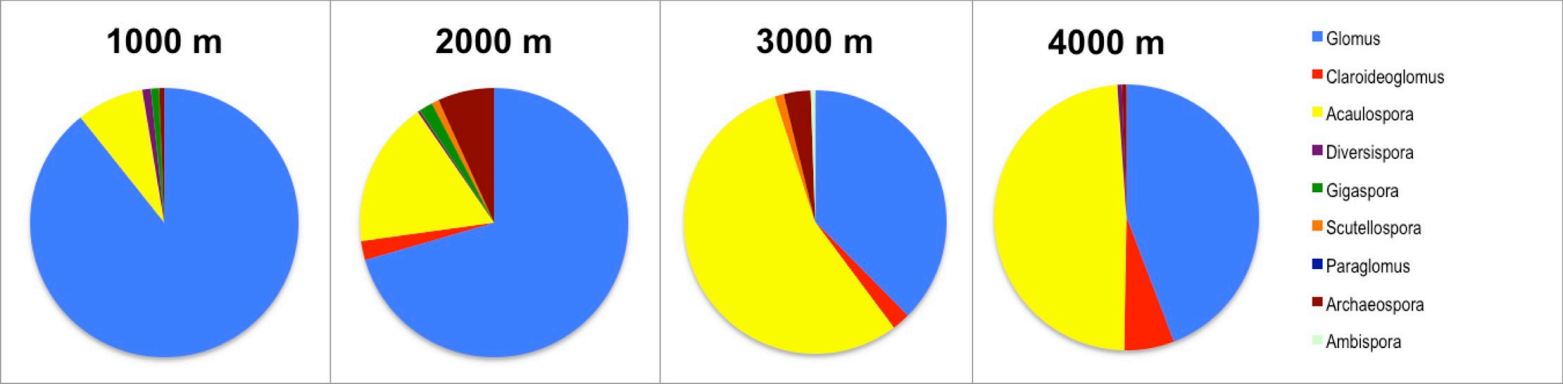
Relative abundance of Glomeromycotina genera for each elevation level in number of sequences. Members of *Glomus* (in light blue) were the dominant mycorrhizal fungi in evergreen premontane forest (≈1000 masl) and the evergreen lower montane forest (≈2000 masl). In the upper montane forest (≈3000 masl) and páramo (≈4000 masl) members of the genus *Acaulospora* (in yellow) were most abundant.

**Fig 5:**
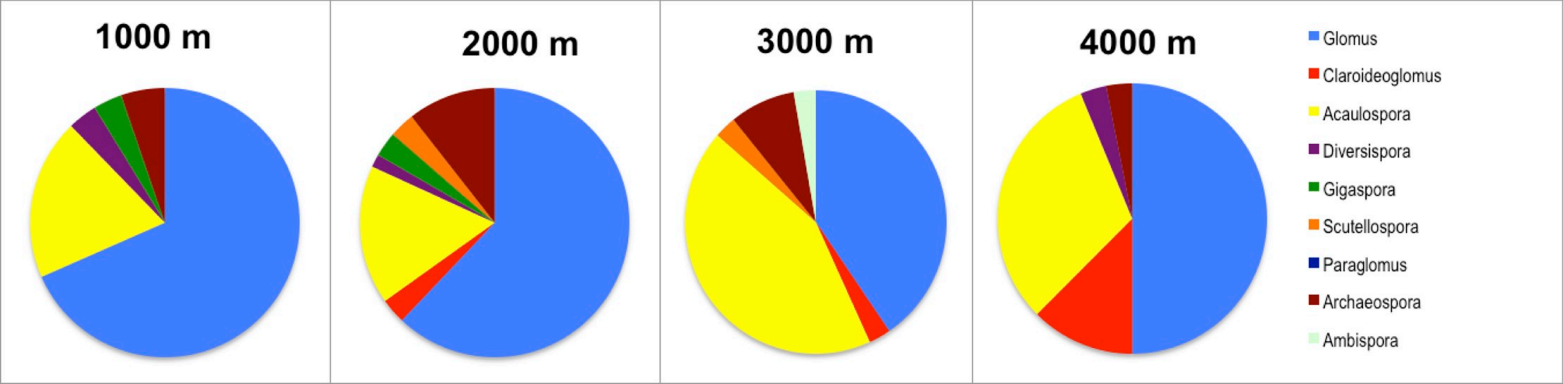
Relative abundance of Glomeromycotina genera for each elevation level in number of OTUs. The frequency of *Glomus* OTUs was high at all levels, the frequency of *Acaulospora* OTUs increased in particular at 3000 masl and also at 4000 masl in comparison to 1000 and 2000 masl.

*Glomus* was the most abundant genus overall in terms of sequence frequency (Fig 4) as well as OTU frequency (Fig 5, S6 Table) followed by *Acaulospora*. However, dominance of *Glomus* decreased at 3000 masl and 4000 masl (Fisher’s Exact Two-Sided Test: p = 0.0154), whereas *Acaulospora* became significantly more abundant at the 3000/4000 masl level in comparison to the 1000/2000 masl level (Fisher’s Exact Two-Sided Test: p = 0.0178). Abundance of all other taxa combined did not shift in higher elevations (Fisher’s Exact Two-Sided Test: p = 1), which does not mean that individual genera are equally frequent or present in all elevations. For example, there was a relatively high frequency of *Claroideoglomus* sequences (6,1%, Fig 4) at the 4000 masl level.

**Fig 6:**
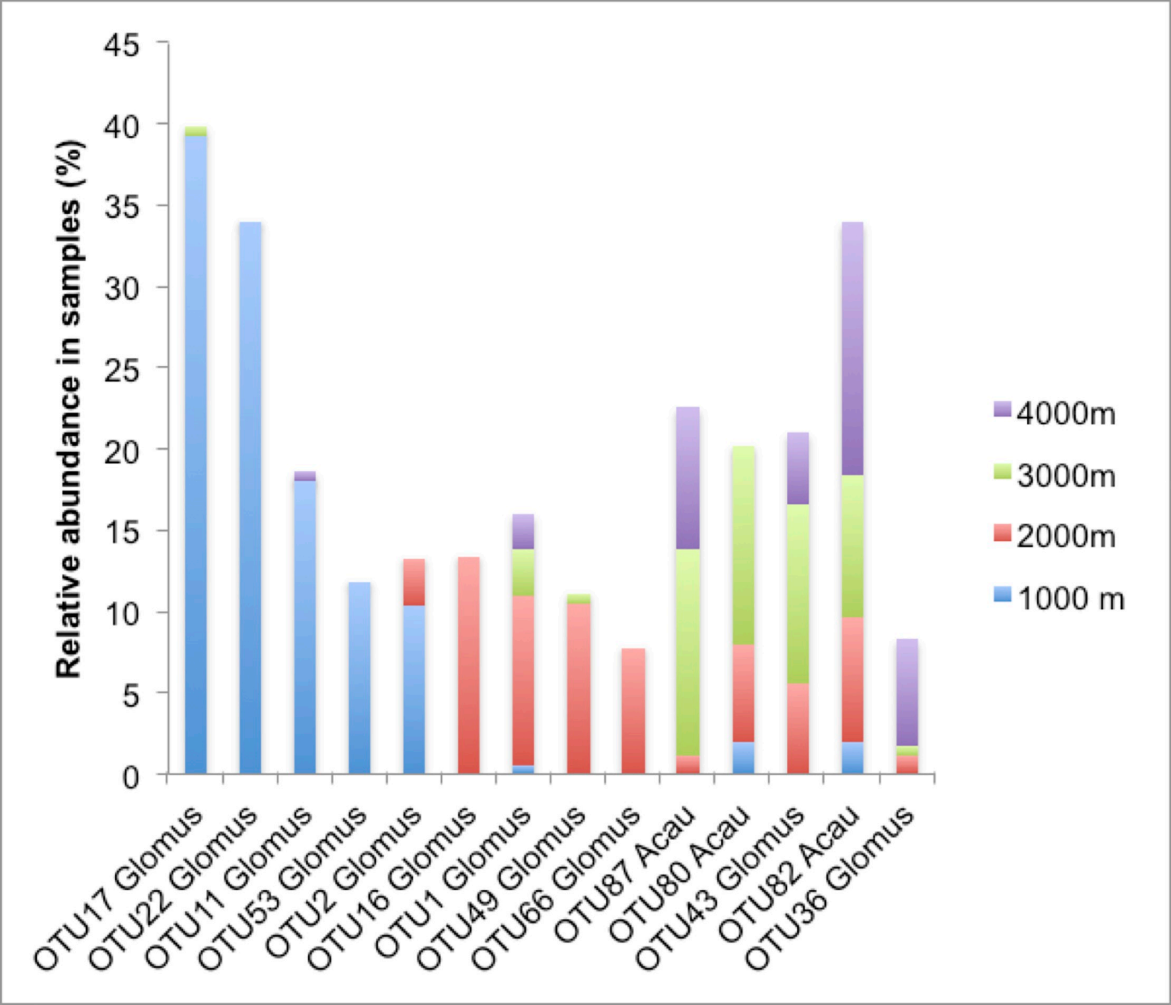
Relative abundance of most frequent OTUs (> 10% of samples) for each elevation level. At the 1000 masl level, five *Glomus* OTUs were frequent with OTU17 and OTU22 occurring in over 30% of all samples. These OTUs showed low frequencies at other elevation levels. At 2000–4000 masl, the most abundant OTU was OTU16 (*Glomus*) at 2000 masl, and the most frequent OTUs at 3000 and 4000 masl were almost identical and also occurred at the other elevations.

Overall, samples (n = 211) from the evergreen premontane forest in Bombuscaro, Podocarpus National Park (1000 masl), revealed 57 AMF OTUs (494 sequences). The rarefaction curve nearly reached the asymptote (S3 Fig), and richness indices estimated a total of 65 to 71 OTUs to be present in the samples (S7 Table). Two *Glomus* OTUs (OTU17, OTU22) were especially abundant occurring in 39% (OTU17) and 34% (OTU22) of all samples (Fig 6). The second most abundant genus was *Acaulospora* comprising an OTU frequency of 19.3% of all OTUs (11 total) and 8.1% of all sequences (Figs 4 and 5, S6 Table). All other genera had low frequencies for both, number of OTUs and sequences and low sequence numbers (Figs 4 and 5, S6 Table).

At the 2000 masl elevation level, 66 OTUs were found (184 samples, 353 sequences). Seventy percent of the sequences belonged to the genus *Glomus* (Fig 4), with the most abundant *Glomus* OTUs being OTU16, OTU49, and OTU1. However, these OTUs occurred only in 10–13% of all samples (Fig 6, S8 Table). *Glomus* was also the most diverse genus displaying 62% of all OTUs (Fig 5, S6 Table), followed by *Acaulospora* (17%) and *Archaeospora* (11%). The genera *Claroideoglomus*, *Diversispora*, *Gigaspora*, and *Scutellospora* had low OTU and sequence (4%) numbers (Figs 4 and 5, S6 Table). The majority of AMF OTUs from RBSF at ≈2000 masl were detected, as shown by the saturated rarefaction curve (S3 Fig).

The analysis of mycorrhizal samples at the 3000 masl elevation level revealed 37 OTUs (181 sequences in 128 samples). This number is lower than the total number of estimated OTUs, as shown by the rarefaction curve not reaching a stable phase (S3 Fig). Richness indices (S7 Table) estimated 49 to 51 OTUs in total (Chao2, Jack1). More than half of the sequences (55%) belonged to the genus *Acaulospora* and 38% to the genus *Glomus* (Fig 4). With 16, respectively 15 OTUs, the genera *Acaulospora* and *Glomus* represented high proportions (43+40 = 83%) of the overall AMF community (Fig 5, S6 Table). All other genera showed very low OTU and sequence numbers (Figs 4 and 5, S6 Table). The most abundant OTUs were *Acaulospora* OTU87 and OTU80 occurring in 12,7%, respectively 12%, of all samples and *Glomus* OTU43 in 11% of all samples (Fig 6, S8 Table). As is the case for the other elevations, many OTUs were rare, with 83% of all OTUs (n = 31) occurring in less than 5% of all samples (S8 Table).

The samples from ≈4000 masl sites at Cajas National Park (n = 123) revealed 32 OTUs (181 sequences). As is the case for the sampling at 3000 masl, the rarefaction curve showed that the area is still under-sampled (S3 Fig.) and richness indices estimated a total of 48 to 45 OTUs (Chao2, Jack1). *Acaulospora* and *Glomus* were, again, the most dominant AMF.

With 49%, respectively 44%, the *Acaulospora*, and *Glomus* sequences were most abundant (Fig 4). With 16 OTUs, the genus *Glomus* represented a high proportion (50%) of the overall AMF community (Fig 5, S6 Table). The second most common genus *Acaulospora* comprised 10 OTUs (31%, Fig 5, S6 Table). There was also a high proportion of *Claroideoglomus* sequences (6,1%, Fig 4) at ≈4000 masl. All other genera showed low OTU and low sequence numbers (Figs 4 and 5, S6 Table). Most OTUs occurred in low numbers with 75% only occurring in up to 5% of the samples. Exceptions are four *Acaulospora* OTUs (82, 23, 25, 36, 41) and four *Glomus* OTUs (23, 25, 36, 41) with occurrences ranging from 6 to 16% of all samples (Fig 6, S8 Table).

There was a pronounced switch from the 1000/2000 masl level to the 3000 masl level in respect to the dominance of *Glomus* and *Acaulospora*. *Glomus* was dominant in lower elevations, whereas *Acaulospora* took over in higher elevations (S6 Table). This result was surprising because we suspected that if a shift in dominance occurred, it would be from ≈3000 masl to ≈4000 masl as this marks the switch from forest vegetation to páramo. Cloud forests and Andean grasslands are various ecosystems with different carbon cycling and retention properties [43].

The dominance of *Glomus* at ≈1000 and ≈2000 masl is in accordance with many other studies using spore-, Sanger-sequence- or NGS-based sampling techniques from different ecosystems (e.g. [3, 44–48]). For *Acaulospora*, however, the literature is controversial. Dominance of Acaulosporaceae in higher elevations, as our results show, is supported by Egan *et al*. [16], who observed an increase in the number of *Acaulospora* species along a high elevation gradient in Montana (USA) and four studies who found the same pattern in the Himalaya [9, 12, 14, 15]. Also, two unknown *Acaulospora* species were the dominant colonizers of Andean potatoes growing between 2658 and 4075 masl [49]. Other studies in the Himalaya did not find increasing *Acaulospora* members at higher elevations [13, 17]. The reasons behind the frequency patterns of Glomeraceae and Acaulosporaceae is not clear and remains to be studied. One reason for this pattern may be the N content in the soil: Nitrogen additions in studies of Camenzind et al. [3] indicate that Diversisporales, the order of Acaulosporaceae, were inhibited by N additions. Soil analyses of our study sites show a decrease of nitrogen from 2000 to 3000 masl (Table 1). Unfortunately no *Acaulospora* species was tested in the experiments by Lerat *et al*. [50, 51] and thus nothing can be said about the carbon-sink strength of the genus *Acaulospora*. Egan *et al*. [16] explained the higher abundance of Acaulosporaceae at higher elevations with the observations of Maherali *et al*. [52] that these fungi produce the least amount of biomass, hence have less demand of N and are thus less taxing for plant hosts in higher elevations.

### AMF communities are phylogenetically clustered at the 1000 m and 2000 m level

All four null models revealed the same results: Mean Nearest Taxon Distance (MNTD) suggested phylogenetic clustering for 1000 masl and 2000 masl and mean pairwise distance (MPD) indicated phylogenetic evenness for all elevation levels by failing to reject the null hypotheses (Table 3). Phylogenetic clustering can be an indicator for habitat filtering and rapid speciation [53] as a driving force in speciation and community building, whereas phylogenetic evenness suggests that stochastic events are more important [53]. However, phylogenetic relatedness is not always correlated with similar traits and similar traits do not only occur in closely related species [54].

**Table 3:** Results of phylogenetic community analyses.

Elevation Level	Null Model	ntaxa	mpd.obs.z	mpd.obs.p	mntd.obs.z	mntd.obs.p
1000 masl	taxa.label	57	-1.4536769	0.082	-2.3974612	0.009*
2000 masl	taxa.label	66	1.1609529	0.888	-2.8744375	0.002**
3000 masl	taxa.label	37	0.4059329	0.657	0.2689927	0.606
4000 masl	taxa.label	32	-0.7284055	0.225	-1.4917689	0.073
1000 masl	phylogeny.pool	57	-1.3769144	0.086	-2.3724285	0.009*
2000 masl	phylogeny.pool	66	1.1336536	0.868	-2.9299391	0.003**
3000 masl	phylogeny.pool	37	0.355155	0.629	0.2947294	0.606
4000 masl	phylogeny.pool	32	-0.8410839	0.19	-1.4743974	0.067
1000 masl	richness	57	-1.4073049	0.084	-2.3473222	0.008*
2000 masl	richness	66	1.1535776	0.875	-2.8451998	0.001**
3000 masl	richness	37	0.3839886	0.65	0.2904128	0.629
4000 masl	richness	32	-0.7406456	0.218	-1.4937621	0.071
1000 masl	trialswap	57	-1.5613924	0.061	-2.0844214	0.026*
2000 masl	trialswap	66	1.0979397	0.863	-2.4227387	0.004**
3000 masl	trialswap	37	0.3798215	0.645	0.7073821	0.773
4000 masl	trialswap	32	-0.7807258	0.205	-1.1262514	0.135

Phylogenetic community analyses results for each elevation level show significantly negative MNTD values for the 1000/2000 masl elevation levels only. Significantly negative MPD and MNTD values indicate phyogenetic clustering. mpd.obs.z: effect size for mean phylogenetic distance; mpd.obs.p: p-value for MPD; mntd.obs.z: effect size for mean nearest taxon distance; mntd.obs.p: p-value for MNTD

* = p.value < 0.05

** = p.value < 0.005.

Phylogenetic clustered AMF communities were also found in three similar elevations (subalpine grassland 1682 masl, treeline 2064 masl, alpine tundra 2225 masl) in the Glacier National Park [16]. Li *et al*. [9] found a positively correlated net relatedness index (NRI) in the Hengduan Mountains region, southwest China, at the belts between 4300 and 5500 m asl.

### Most abundant OTUs from the study area are not endemic to Southern Ecuador

To gain information about distribution patterns of abundant AMF from this study, we compared the OTUs to their closest hits from the MaarjAM database (S9 Table). The results should be considered with caution because data on AMF occurrences are still very fragmented and many areas are undersampled. Many AMF from our study sites were found to have a worldwide distribution. One of the most widespread AMF OTUs with global distribution and occurring in different biomes belongs to the *Rhizophagus intraradices/irregularis/vesiculiferus*-group (MaarjAM ID: VT113/114/115 - [55, 56]) here called OTU1. This generalist OTU was abundant at the 2000 masl level. Another globally distributed generalist is OTU43 (MaarjAM ID: VT191), belonging to Glomeraceae. Found worldwide but restricted to forests, is OTU16 (MaarjAM ID: VT183). This OTU belongs to the family Glomeraceae and we found it at the 2000 masl level. Another OTU restricted to 2000 masl level in our study was OTU49 (Glomeraceae), the type sequence for VT183 (MaarjAM ID). OTU49 might be occurring only in tropical rain forests as it is so far only known from tropical rainforests in French Guiana, Gabon and Ecuador. The most abundant OTUs at 1000 m belt (*Glomus* OTUs 17 and 22) and found in our study only in this belt, have no close match neither in the MaarjAM nor the NCBI database (S9 Table). It remains to be seen whether the endemic status of OTU17 and 22 will hold with more sampling in other areas in South America or worldwide.

Three *Acaulospora* OTUs were abundant in our study area: OTU80 (Maarjam ID: VT12), an OTU occurring from ≈1000 masl to ≈3000 masl but with main abundance in the 3000 masl level, is otherwise only known from South America (Argentina and French Guiana); OTU 82 (Maarjam ID: VT14), a generalist that also occurs in Europe and Asia; and OTU87 (Maarjam ID: VT30) a ≈3000 masl specialist in our study that has worldwide distribution and is found in many biomes.

### Rare OTUs in AMF communities and their potential role in ecosystem functioning

Like previous studies of AM fungal community structure [57, 58] we found a high proportion of rare OTUs at all elevations: 79–85% of OTUs occurred in less than 5% of the samples (S8 Table). However, dominance of a single taxon representing on average 40% of total abundance as shown in previous studies [57, 58] was only present at the 1000 masl level. At higher elevations, the most common OTUs were detected in only 13–15% of the samples. This mirrors the situation for plants in tropical forests [59]. The processes behind these patterns are not fully understood, not even for plants [60]. Niche differentiation is considered to be an important factor in the structuring of communities, for plants [59, 60] and AM fungi [61], but stochastic processes also play a role [30, 62]. The study sites we investigated had a broad range of edaphic variability at each elevation, which could provide many microhabitats for AMF to specialize in.

The importance of rare plant species [63] and their distributions across ecosystems is discussed in several studies (e.g. [64, 65]). Determining the ecological similarity of rare and common species is not easy, particularly when a large number of species are is involved. We can assume, though, that the more species and the higher the phylogenetic diversity, the more traits are available in a community. The multitude of rare fungi in our study with a broad phylogenetic level at each elevation thus offers a wide range of traits with potential benefits to their plant partners. Therefore, rare species might be important for maintaining ecosystem functioning in a changing environment [66]. We can see evidence for this in our data as some generalist OTUs (e.g. OTU1, OTU43, OTU80, OTU82, OTU87) are rare at a certain elevation but become dominant in another elevations. More studies dedicated to the phenomenon of rare species are needed, though, as a better understanding of dominance and rarity in AMF might help to model AMF community structures in a changing climate.

### Future directions

Elevational changes correlate with many factors such as temperature, precipitation, and nutrient availability. Not only abiotic but also biotic factors change, e.g., vegetation and host plant availability. In this study, we only had few biotic and abiotic data available, and thus could not determine the driving factor behind the elevational turnover of AMF. Another factor that should be taken into account in future studies is nutrient availability, e.g., nitrogen and phosphorus sources but also carbon provided by host plants. Moser *et al*. [24] showed that the leaf area index (LAI—Table 1) decreases by 40–60% between 1000 and 3000 masl. This indicates that canopy carbon gain decreases with elevation and thus the amount of sugar that plants release to their AM fungi might decrease as well. In-vitro experiments showed different C-sink strength for different AM fungi [50, 51] and Kiers *et al*. [67] showed that plants could detect different AMF partners and therefore plants with lower LAI might select AMF with lower sugar requirements. Further studies in the field and under defined conditions are needed to unravel the factors that determine the composition of the AMF communities.

## Supporting information

S1 Fig Maximum Likelihood tree RAxML of the dataset. (TXT)

S2 Fig Shared and unique OTUs of each elevation belt. The horizontal bars connecting the same elevation indicate the amount of unique OTUs for this elevation. The highest number of unique OTUs both in richness and frequency was in the 1000 masl belt with a downward trend towards higher elevations. The thickness of connector lines represents the percentage of OTUs shared. The neighboring elevation levels 1000/2000 masl and 3000/4000 masl showed a high overlap of OTUs.
(TIFF)

S3 Fig Rarefaction analyses for each elevational belt. (TIFF)

S1 Table Sampling subsites with number of samples taken and sequences obtained per site. Abbreviations: **B1, B2, B3** Bombuscaro plot 1, 2, 3, **Q2, Q5** ravines, **T1, T2** ridges, **Cc** Cajanuma PNP Upper Montane Forest 2700 m, **Cm** Cajanuma PNP Upper Montane Forest 3000 m, **Cl** Cajanuma PNP Upper Montane Forest 2800 m, **Clp** Cajanuma PNP Shrub Páramo 3100 m, **N** Cajas NP Nero Shrub Páramo 3250 m, **Tu** Cajas NP Tutupali Grass Páramo 3500 m, **So** Cajas NP Soldados Grass Páramo 3750 m, **Cpo** Cajas NP Toreadora *Polylepis* 4000 m, **Cpa** Cajas NP Toreadora Grass Páramo 4000 m.
(PDF)

S2 Table List of sequences with accession number, site, OTU number and genus of Glomeromycotina. Abbreviations same as in S1 Table.
(PDF)

S3 Table List of sequences and corresponding OTU numbers with a cut-off value of 97% sequence similarity. (PDF)

S4 Table Shared OTUs between elevational belts. (PDF)

S5 Table Similarity indices. (PDF)

S6 Table Number of OTUs and number of sequences for the different genera at the elevational belts. (PDF)

S7 Table Richness indices. (PDF)

S8 Table Frequencies of OTUs. (**A)** Frequency of OTUs, divided into 4 classes: number of OTUs in <1% of the samples, in 1–5% of samples, in 5–10% of samples, in ≥10% of samples; (**B)** OTUs occurring in ≥ 10% of samples of the elevational belt.
(PDF)

S9 Table List of OTUs with number of sequences at the elevation levels and closest BLAST matches. Coloured backgrounds: Lilac: *Glomus*, green: *Acaulospora*, blue: *Diversispora*, red: *Gigaspora*, brown: *Scutellospora*, rose: *Claroideoglomus*, cyan: *Archaeospora*, light green: *Ambispora*. The red numbers indicate the most abundant OTUs.
(PDF)
