# Supplementary figures and images for: Species composition of arbuscular mycorrhizal communities changes with elevation in the Andes of South Ecuador
Source: PLoS One. 2019 Aug 16;14(8):e0221091. doi: 10.1371/journal.pone.0221091 (PMC6697372; doi:10.1371/journal.pone.0221091)

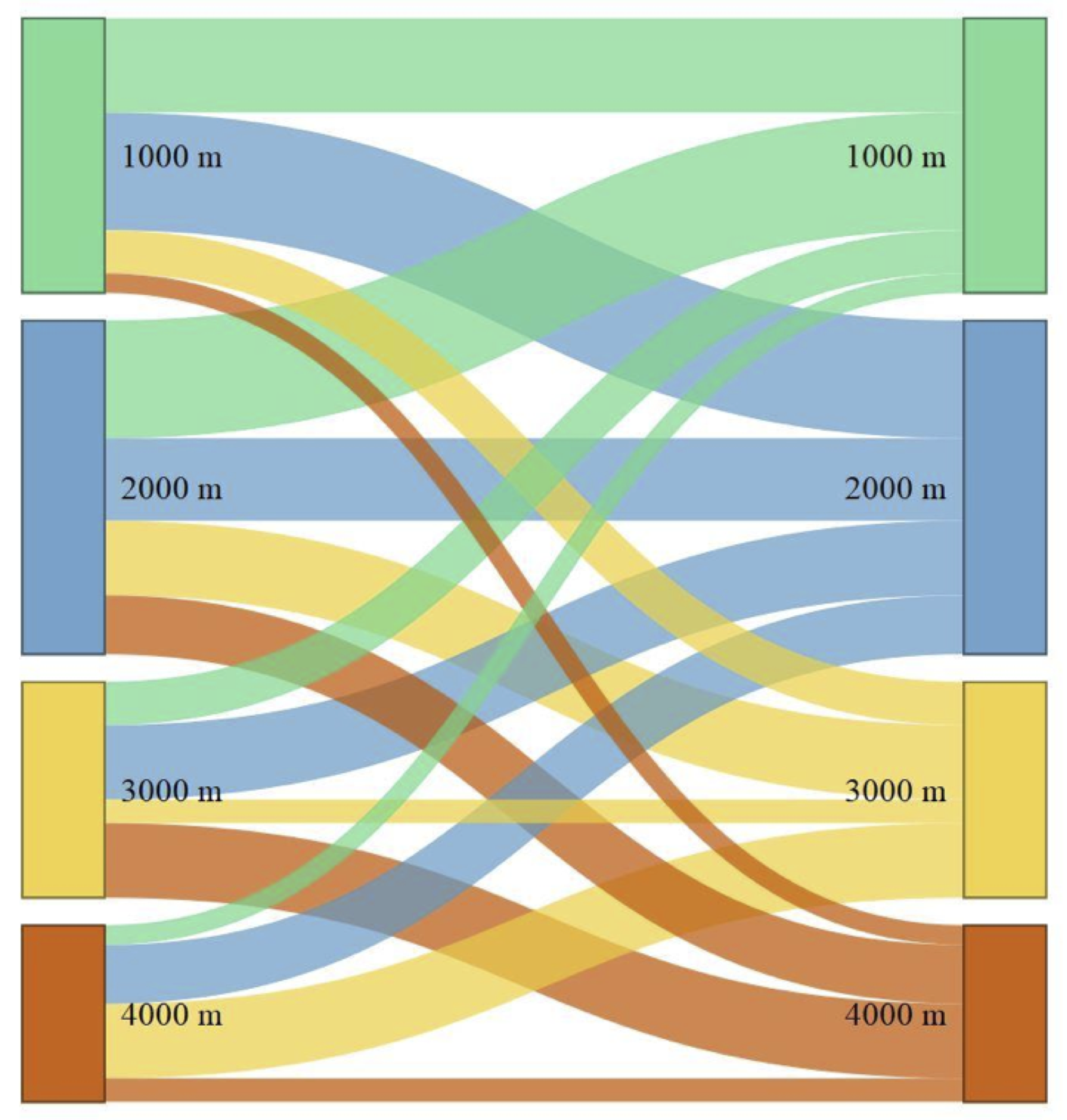

Supplement: S2 Fig — The horizontal bars connecting the same elevation indicate the amount of unique OTUs for this elevation. The highest number of unique OTUs both in richness and frequency was in the 1000 masl belt with a downward trend towards higher elevations. The thickness of connector lines represents the percentage of OTUs shared. The neighboring elevation levels 1000/2000 masl and 3000/4000 masl showed a high overlap of OTUs. (TIFF) [file pone.0221091.s002.tiff]
