# Supplementary material for: Species composition of arbuscular mycorrhizal communities changes with elevation in the Andes of South Ecuador
Source: PLoS One. 2019 Aug 16;14(8):e0221091. doi: 10.1371/journal.pone.0221091 (PMC6697372; doi:10.1371/journal.pone.0221091)
Supplement: S1 Table — Abbreviations: B1, B2, B3 Bombuscaro plot 1, 2, 3, Q2, Q5 ravines, T1, T2 ridges, Cc Cajanuma PNP Upper Montane Forest 2700 m, Cm Cajanuma PNP Upper Montane Forest 3000 m, Cl Cajanuma PNP Upper Montane Forest 2800 m, Clp Cajanuma PNP Shrub Páramo 3100 m, N Cajas NP Nero Shrub Páramo 3250 m, Tu Cajas NP Tutupali Grass Páramo 3500 m, So Cajas NP Soldados Grass Páramo 3750 m, Cpo Cajas NP Toreadora Polylepis 4000 m, Cpa Cajas NP Toreadora Grass Páramo 4000 m. (PDF) [file pone.0221091.s004.pdf]

S1 Table. Sampling subsites with number of samples taken and sequences obtained per site

Abbreviations: **B1, B2, B3** Bombuscaro plot 1, 2, 3, **Q2, Q5** ravines, **T1, T2** ridges, **Cc** Cajanuma PNP Upper Montane Forest 2700 m, **Cm** Cajanuma PNP Upper Montane Forest 3000 m, **Cl** Cajanuma PNP Upper Montane Forest 2800 m, **Clp** Cajanuma PNP Shrub Páramo 3100 m, **N** Cajas NP Nero Shrub Páramo 3250 m, **Tu** Cajas NP Tutupali Grass Páramo 3500 m, **So** Cajas NP Soldados Grass Páramo 3750 m, **Cpo** Cajas NP Toreadora *Polylepis* 4000 m, **Cpa** Cajas NP Toreadora Grass Páramo 4000 m

	**1000 m evergreen premontane forest**			**2000 m evergreen lower montane forest**				**3000 m upper montane forest/ shrub páramo**					**4000 m grass páramo/Polylepis forest**			
	**1000 - 1140 m**			**1900 - 2500 m**				**2880 - 3250 m**					**3500 - 4000 m**			
	B1	B2	B3	Q2	Q5	T1	T2	Cc	Cm	Cl	Clp	N	Tu	So	Cpo	Cpa
Mycorrhizal samples from former studies				10	48	6	34									
Mycorrhizal samples from this study	103	82	26		15		71	36	21	46	17	8	20	15	32	56
Total of samples analyzed in this study	**103**	**82**	**26**	**10**	**63**	**6**	**105**	**36**	**21**	**46**	**17**	**8**	**20**	**15**	**32**	**56**
Sequences from former studies				13	141	15	46									
Sequences from this study	246	193	55		32		106	55	28	67	20	11	30	29	41	81
**Total of sequences in this study**	**246**	**193**	**55**	**13**	**173**	**15**	**152**	**55**	**28**	**67**	**20**	**11**	**30**	**29**	**41**	**81**
