# Supplementary material for: Species composition of arbuscular mycorrhizal communities changes with elevation in the Andes of South Ecuador
Source: PLoS One. 2019 Aug 16;14(8):e0221091. doi: 10.1371/journal.pone.0221091 (PMC6697372; doi:10.1371/journal.pone.0221091)
Supplement: S2 Table — Abbreviations same as in S1 Table. (PDF) [file pone.0221091.s005.pdf]

S2 Tab				
**Object**	**Site**	**OTU No**	**Genus**	**randomly picked sequences for MPD and MNTI**
1_K493_2_KX108455_BombuscaroI_1s	B1	17	Glomus	
1_K494_1_KX108346_BombuscaroI_2s	B1	22	Glomus	x
1_K494_4_KX108392_BombuscaroI_2s	B1	17	Glomus	
1_K495_1_KX108444_BombuscaroI_3s	B1	17	Glomus	
1_K496_5_KX108260_BombuscaroI_5g	B1	11	Glomus	
1_K497_1_KX108446_BombuscaroI_6s	B1	17	Glomus	
1_K497_3_KX108354_BombuscaroI_6s	B1	22	Glomus	
1_K498_1_KX108198_BombuscaroI_7s	B1	0	Glomus	
1_K498_2_KX108600-BombuscaroI_7s	B1	62	Glomus	
1_K498_3_KX108366_BombuscaroI_7s	B1	22	Glomus	
1_K498_6_KX108666_BombuscaroI_7s	B1	94	Acaulospora	
1_K499_1_KX108198_BombuscaroI_8s	B1	22	Glomus	
1_K499_6_KX108432_BombuscaroI_8s	B1	17	Glomus	
1_K499_8_KX108652_BombuscaroI_8s	B1	93	Acaulospora	
1_K500_1_KX108360_BombuscaroI_9s	B1	22	Glomus	
1_K501_1_KX108603_BombuscaroI_10s	B1	62	Glomus	x
1_K501_2_KX108225_BombuscaroI_10s	B1	32	Glomus	x
1_K502_1_KX108324_BombuscaroI_11s	B1	22	Glomus	
1_K502_2_KX108419_BombuscaroI_11s	B1	17	Glomus	
1_K503_1_KX108650_BombuscaroI_12	B1	88	Acaulospora	
1_K503_2_KX108584_BombuscaroI_12	B1	68	Glomus	
1_K503_4_KX108415_BombuscaroI_12	B1	17	Glomus	
1_K503_6_KX108353_BombuscaroI_12	B1	22	Glomus	
1_K503_8_KX108485_BombuscaroI_12	B1	20	Glomus	
1_K504_2_KX108557_BombuscaroI_13	B1	50	Glomus	
1_K505_1_KX108532_BombuscaroI_14	B1	53	Glomus	
1_K505_2_KX108553_BombuscaroI_14	B1	50	Glomus	
1_K506_1_KX108560_BombuscaroI_15	B1	50	Glomus	
1_K506_2_KX108254_BombuscaroI_15	B1	11	Glomus	
1_K506_3_KX108420_BombuscaroI_15	B1	17	Glomus	
1_K507_1_KX108535_BombuscaroI_16	B1	53	Glomus	
1_K507_2_KX108574_BombuscaroI_16	B1	54	Glomus	
1_K508_1_KX108363_BombuscaroI_17	B1	22	Glomus	
1_K508_2_KX108565_BombuscaroI_17	B1	50	Glomus	
1_K508_3_KX108259_BombuscaroI_17	B1	11	Glomus	
1_K512_1_KX108351_BombuscaroI_4	B1	22	Glomus	
1_K512_2_KX108488_BombuscaroI_4	B1	20	Glomus	
1_K513_2_KX108393_BombuscaroI_18	B1	17	Glomus	
1_K513_5_KX108654_BombuscaroI_18	B1	93	Acaulospora	
1_K514_1_KX108224_BombuscaroI_19	B1	32	Glomus	

1_K514_2_KX108630_BombuscaroI	B1	46	Glomus	
1_K514_4_KX108397_BombuscaroI_19	B1	17	Glomus	
1_K515_1_KX108563_BombuscaroI_20	B1	50	Glomus	
1_K515_2_KX108631_BombuscaroI	B1	46	Glomus	x
1_K516_1_KX108690_BombuscaroI_21	B1	108	Gigaspora	
1_K516_2_KX108495_BombuscaroI_21	B1	41	Glomus	
1_K516_8_KX108641_BombuscaroI_21	B1	80	Acaulospora	
1_K517_1_KX108272_BombuscaroI_22	B1	11	Glomus	
1_K517_2_KX108437_BombuscaroI_22	B1	17	Glomus	
1_K517_3_KX108649_BombuscaroI_22	B1	83	Acaulospora	
1_K518_1_KX108403_BombuscaroI_23b	B1	17	Glomus	
1_K518_4_KX108642_BombuscaroI_23b	B1	80	Acaulospora	
1_K519_1_KX108594_BombuscaroI_24	B1	61	Glomus	
1_K519_2_KX108355_BombuscaroI_24	B1	22	Glomus	
1_K519_3_KX108216_BombuscaroI_24	B1	2	Glomus	
1_K520_1_KX108205_BombuscaroI_26	B1	2	Glomus	
1_K520_2_KX108588_BombuscaroI_26	B1	61	Glomus	
1_K520_3_KX108398_BombuscaroI_26	B1	17	Glomus	
1_K520_4_KX108533_BombuscaroI_26	B1	53	Glomus	
1_K521_1_KX108475_BombuscaroI_27	B1	17	Glomus	
1_K521_3_KX108352_BombuscaroI_27	B1	22	Glomus	
1_K521_4_KX108541_BombuscaroI_27	B1	53	Glomus	
1_K522_2_KX108547_BombuscaroI_28	B1	53	Glomus	
1_K522_4_KX108402_BombuscaroI_57	B1	17	Glomus	
1_K523_2_KX108290_BombuscaroI_29	B1	10	Glomus	
1_K523_3_KX108534_BombuscaroI_29	B1	53	Glomus	
1_K523_5_KX108395_BombuscaroI_29	B1	17	Glomus	
1_K524_1_KX108660_BombuscaroI_30	B1	94	Acaulospora	
1_K524_2_KX108476_BombuscaroI_30	B1	17	Glomus	
1_K525_9_KX108527_BombuscaroI_31	B1	53	Glomus	
1_K527_3_KX108373_BombuscaroI_33	B1	22	Glomus	
1_K527_8_KX108282_BombuscaroI_33	B1	11	Glomus	
1_K528_1_KX108516_BombuscaroI_33	B1	60	Glomus	
1_K528_2_KX108321_BombuscaroI_33	B1	22	Glomus	
1_K529_1_KX108487_BombuscaroI_34	B1	20	Glomus	
1_K529_4_KX108383_BombuscaroI_34	B1	22	Glomus	
1_K530_1_KX108653_BombuscaroI_35	B1	93	Acaulospora	
1_K530_2_KX108597_BombuscaroI_35	B1	68	Glomus	
1_K531_1_KX108362_BombuscaroI_36	B1	22	Glomus	
1_K531_2_KX108658_BombuscaroI_36	B1	94	Acaulospora	
1_K531_5_KX108517_BombuscaroI_36	B1	60	Glomus	
1_K532_1_KX108688_BombuscaroI_37	B1	128	Archaeospora	
1_K532_2_KX108394_BombuscaroI_37	B1	17	Glomus	
1_K532_7_KX108372_BombuscaroI_37	B1	22	Glomus	
1_K533_10_KX108552_BombuscaroI_38	B1	50	Glomus	
1_K533_14_KX108540_BombuscaroI_38	B1	53	Glomus	

1_K533_16_KX108421_BombuscaroI_38	B1	17	Glomus	
1_K533_9_KX108569_BombuscaroI_38	B1	54	Glomus	x
1_K534_12_KX108357_BombuscaroI_39	B1	22	Glomus	
1_K535_10_KX108449_BombuscaroI_40	B1	17	Glomus	
1_K535_11_KX108595_BombuscaroI_40	B1	61	Glomus	
1_K535_14_KX108206_BombuscaroI_40	B1	2	Glomus	
1_K536_1_KX108350_BombuscaroI_41g	B1	22	Glomus	
1_K536_4_KX108502_BombuscaroI_41g	B1	47	Glomus	
1_K536_5_KX108599_BombuscaroI_41g	B1	62	Glomus	
1_K536_7_KX108463_BombuscaroI_41g	B1	17	Glomus	
1_K537_1_KX108643_BombuscaroI_42	B1	80	Acaulospora	
1_K537_4_KX108661_BombuscaroI_42	B1	95	Acaulospora	
1_K538_1_KX108450_BombuscaroI_43	B1	17	Glomus	
1_K538_4_KX108598_BombuscaroI_43	B1	62	Glomus	
1_K538_5_KX108308_BombuscaroI_43	B1	30	Glomus	
1_K539_1_KX108662_BombuscaroI_44	B1	95	Acaulospora	
1_K539_2_KX108679_BombuscaroI_44	B1	101	Acaulospora?	
1_K540_1_KX108480_BombuscaroI	B1	19	Glomus	x
1_K540_2_KX108587_BombuscaroI_45	B1	61	Glomus	
1_K540_5_KX108663_BombuscaroI_45	B1	95	Acaulospora	
1_K540_6_KX108644_BombuscaroI_45	B1	80	Acaulospora	
1_K541_4_KX108331_BombuscaroI_46	B1	22	Glomus	
1_K541_5_KX108405_BombuscaroI_46	B1	17	Glomus	
1_K542_14_KX108414_BombuscaroI_47	B1	17	Glomus	
1_K542_5_KX108484_BombuscaroI_47	B1	20	Glomus	
1_K542_7_KX108593_BombuscaroI_47	B1	61	Glomus	
1_K543_10_KX108486_BombuscaroI_48	B1	20	Glomus	
1_K543_11_KX108664_BombuscaroI_48	B1	95	Acaulospora	
1_K543_12_KX108576_BombuscaroI_48	B1	68	Glomus	
1_K543_9_KX108369_BombuscaroI_48	B1	22	Glomus	
1_K544_10_KX108592_BombuscaroI_49	B1	61	Glomus	x
1_K545_5_KX108251_BombuscaroI	B1	33	Glomus	
1_K546_1_KX108311_BombuscaroI_51	B1	21	Glomus	
1_K546_7_KX108364_BombuscaroI_51	B1	22	Glomus	
1_K547_1_KX108575_BombuscaroI_52	B1	68	Glomus	
1_K547_7_KX108213_BombuscaroI_52	B1	2	Glomus	
1_K547_8_KX108447_BombuscaroI_52	B1	17	Glomus	
1_K548_1_KX108341_BombuscaroI_53g	B1	22	Glomus	
1_K548_2_KX108477_BombuscaroI_53g	B1	17	Glomus	
1_K548_5_KX108296_BombuscaroI_53g	B1	12	Glomus	
1_K548_6_KX108564_BombuscaroI_53g	B1	50	Glomus	
1_K549_2_KX108266_BombuscaroI_54g	B1	11	Glomus	
1_K549_4_KX108375_BombuscaroI_54g	B1	22	Glomus	
1_K550_1_KX108441_BombuscaroI_55	B1	17	Glomus	
1_K550_2_KX108380_BombuscaroI_55	B1	22	Glomus	
1_K550_3_KX108214_BombuscaroI_55	B1	2	Glomus	

1_K551_1_KX108589_BombuscaroI_56	B1	61	Glomus	
1_K551_3_KX108433_BombuscaroI_56	B1	17	Glomus	
1_K551_6_KX108367_BombuscaroI_56	B1	22	Glomus	
1_K552_1_KX108448_BombuscaroI_57	B1	17	Glomus	
1_K553_1_KX108207_BombuscaroI_58g	B1	2	Glomus	x
1_K553_2_KX108265_BombuscaroI_58g	B1	11	Glomus	
1_K554_1_KX108281_BombuscaroI_59g	B1	11	Glomus	
1_K554_2_KX108422_BombuscaroI_59g	B1	17	Glomus	
1_K554_4_KX108212_BombuscaroI_59g	B1	2	Glomus	
1_K555_1_KX108548_BombuscaroI_60	B1	53	Glomus	
1_K555_2_KX108673_BombuscaroI_50	B1	97	Acaulospora	x
1_K555_4_KX108323_BombuscaroI_60	B1	22	Glomus	
1_K556_2_KX108479_BombuscaroI_61	B1	19	Glomus	
1_K556_3_KX108348_BombuscaroI_61	B1	22	Glomus	
1_K557_2_KX108325_BombuscaroI_62	B1	22	Glomus	
1_K557_3_KX108396_BombuscaroI_62	B1	17	Glomus	
1_K558_1_KX108358_BombuscaroI_63	B1	22	Glomus	
1_K559_3_KX108332_BombuscaroI_63	B1	22	Glomus	
1_K559_7_KX108250_BombuscaroI_63	B1	33	Glomus	
1_K560_1_KX108566_BombuscaroI_64s	B1	50	Glomus	
1_K560_2_KX108322_BombuscaroI_64s	B1	22	Glomus	
1_K560_3_KX108413_BombuscaroI_64s	B1	17	Glomus	
1_K560_6_KX108655_BombuscaroI_64s	B1	93	Acaulospora	x
1_K561_2_KX108551_BombuscaroI_65s	B1	50	Glomus	
1_K561_4_KX108345_BombuscaroI_56s	B1	22	Glomus	
1_K562_2_KX108356_BombuscaroI_66	B1	22	Glomus	
1_K563_2_KX108435_BombuscaroI_66	B1	17	Glomus	
1_K563_4_KX108320_BombuscaroI_66	B1	22	Glomus	
1_K564_2_KX108289_BombuscaroI_67	B1	10	Glomus	
1_K564_3_KX108315_BombuscaroI_67	B1	21	Glomus	
1_K564_5_KX108408_BombuscaroI_67	B1	17	Glomus	
1_K565_10_KX108665_BombuscaroI_68b	B1	94	Acaulospora	x
1_K565_15_KX108368_BombuscaroI_68b	B1	22	Glomus	
1_K565_16_KX108651_BombuscaroI_68	B1	88	Acaulospora	
1_K566_11_KX108608_BombuscaroI	B1	63	Glomus	x
1_K566_16_KX108567_BombuscaroI_68b	B1	50	Glomus	
1_K566_9_KX108501_BombuscaroI_68b	B1	47	Glomus	
1_K567_1_KX108389_BombuscaroI_69	B1	22	Glomus	
1_K567_11_KX108210_BombuscaroI_69	B1	2	Glomus	
1_K567_2_KX108568_BombuscaroI_69	B1	53	Glomus	
1_K567_8_KX108452_BombuscaroI_69	B1	17	Glomus	
1_K568_2_KX108404_BombuscaroI_70	B1	17	Glomus	
1_K568_3_KX108326_BombuscaroI_70	B1	22	Glomus	
1_K569_2_KX108407_BombuscaroI_71	B1	17	Glomus	
1_K569_3_KX108571_BombuscaroI_71	B1	54	Glomus	
1_K569_4_KX108680_BombuscaroI_71	B1	101	Acaulospora?	x

1_K569_6_KX108329_BombuscaroI_71	B1	22	Glomus	
1_K570_1_KX108208_BombuscaroI_72	B1	2	Glomus	
1_K570_2_KX108371_BombuscaroI_72	B1	22	Glomus	
1_K570_4_KX108536_BombuscaroI_72	B1	53	Glomus	
1_K571_1_KX108299_BombuscaroI_73	B1	29	Glomus	x
1_K571_2_KX108423_BombuscaroI_73	B1	17	Glomus	
1_K571_3_KX108305_BombuscaroI_73	B1	30	Glomus	x
1_K572_1_KX108604_BombuscaroI_74	B1	62	Glomus	
1_K572_2_KX108327_BombuscaroI_74	B1	22	Glomus	
1_K572_3_KX108298_BombuscaroI_74	B1	29	Glomus	
1_K572_4_KX108411_BombuscaroI_74	B1	17	Glomus	
1_K572_6_KX108307_BombuscaroI_74	B1	30	Glomus	
1_K573_1_KX108466_BombuscaroII_5	B2	17	Glomus	
1_K573_3_KX108276_BombuscaroII_5	B2	11	Glomus	
1_K574_1_KX108200_BombuscaroI_75	B1	2	Glomus	
1_K574_2_KX108328_BombuscaroI_75	B1	22	Glomus	
1_K574_3_KX108681_BombuscaroI_75	B1	101	Acaulospora?	
1_K575_1_KX108426_BombuscaroI_76	B1	17	Glomus	
1_K575_2_KX108481_BombuscaroI	B1	19	Glomus	
1_K576_5_KX108482_BombuscaroII	B2	18	Glomus	x
1_K577_1_KX108255_BombuscaroII_1b	B2	11	Glomus	
1_K577_2_KX108622_BombuscaroII_1b	B2	65	Glomus	
1_K577_6_KX108509_BombuscaroII_1b	B2	57	Glomus	
1_K578_1_KX108478_BombuscaroII_2b	B2	18	Glomus	
1_K578_3_KX108645_BombuscaroII_2b	B2	82	Acaulospora	
1_K578_5_KX108508_BombuscaroII_2b	B2	57	Glomus	
1_K578_7_KX108518_BombuscaroII_2b	B2	58	Glomus	x
1_K579_1_KX108203_BombuscaroII_3	B2	2	Glomus	
1_K579_5_KX108682_BombuscaroII_3	B2	105	Diversispora	
1_K579_7_KX108647_BombuscaroII_3	B2	82	Acaulospora	
1_K580_1_KX108457_BombuscaroII_4	B2	17	Glomus	
1_K580_2_KX108384_BombuscaroII_4	B2	22	Glomus	
1_K581_1_KX108267_BombuscaroII_5	B2	11	Glomus	
1_K582_1_KX108456_BombuscaroII_6b	B2	17	Glomus	
1_K582_7_KX108387_BombuscaroII_6b	B2	22	Glomus	
1_K583_1_KX108504_BombuscaroII_6b	B2	57	Glomus	
1_K583_5_KX108472_BombuscaroII_6b	B2	17	Glomus	
1_K584_1_KX108506_BombuscaroII_7b	B2	57	Glomus	
1_K584_14_KX108277_BombuscaroII_7b	B2	11	Glomus	
1_K585_2_KX108623_BombuscaroII_8	B2	65	Glomus	
1_K585_3_KX108490_BombuscaroII_8	B2	20	Glomus	
1_K585_4_KX108490_BombuscaroII_8	B2	17	Glomus	
1_K586_2_KX108511_BombuscaroII_9b	B2	57	Glomus	
1_K586_4_KX108618_BombuscaroII_9b	B2	64	Glomus	
1_K590_14_KX108674_BombuscaroII_11b	B2	100	Acaulospora?	x
1_K590_9_KX108365_BombuscaroII_11b	B2	22	Glomus	

1_K591_10_KX108483_BombuscaroII	B2	18	Glomus	
1_K591_15_KX108425_BombuscaroII_12	B2	17	Glomus	
1_K591_5_KX108667_BombuscaroII_12	B2	94	Acaulospora	
1_K591_9_KX108365_BombuscaroII_12	B2	99	Acaulospora?	
1_K593_1_KX108580_BombuscaroII_13	B2	68	Glomus	
1_K593_2_KX108619_BombuscaroII_13	B2	64	Glomus	
1_K593_7_KX108347_BombuscaroII_13	B2	22	Glomus	
1_K594_3_KX108424_BombuscaroII_14s	B2	17	Glomus	
1_K594_5_KX108610_BombuscaroII_14s	B2	63	Glomus	
1_K594_7_KX108678_BombuscaroII_14s	B2	100	Acaulospora?	
1_K595_1_KX108342_BombuscaroII_14s	B2	22	Glomus	
1_K595_4_KX108562_BombuscaroII_14s	B2	50	Glomus	
1_K596_1_KX108381_BombuscaroII_15g	B2	22	Glomus	
1_K596_2_KX108459_BombuscaroII_15g	B2	17	Glomus	
1_K597_6_KX108637_BombuscaroII_16b	B2	72	Glomus	
1_K597_8_KX108221_BombuscaroII_16b	B2	2	Glomus	
1_K598_6_KX108634_BombuscaroII_17	B2	72	Glomus	
1_K599_3_KX108624_BombuscaroII	B2	69	Glomus	
1_K599_8_KX108684_BombuscaroII_18	B2	107	Diversispora	
1_K600_12_KX108638_BombuscaroII_18	B2	72	Glomus	
1_K600_9_KX108628_BombuscaroII_18	B2	70	Glomus	
1_K602_10_KX108219_BombuscaroII_19	B2	2	Glomus	
1_K602_13_KX108428_BombuscaroII_19	B2	17	Glomus	
1_K602_6_KX108343_BombuscaroII_19	B2	22	Glomus	
1_K602_9_KX108635_BombuscaroII_19	B2	72	Glomus	
1_K603_1_KX108268_BombuscaroII_20g	B2	11	Glomus	
1_K603_2_KX108460_BombuscaroII_20g	B2	17	Glomus	
1_K603_7_KX108512_BombuscaroII_20g	B2	57	Glomus	
1_K604_7_KX108499_BombuscaroII_21s	B2	44	Glomus	
1_K605_1_KX108585_BombuscaroII_22	B2	68	Glomus	
1_K605_4_KX108261_BombuscaroII_22	B2	11	Glomus	
1_K606_1_KX108586_BombuscaroII_23	B2	68	Glomus	
1_K606_2_KX108612_BombuscaroII_23	B2	63	Glomus	
1_K606_5_KX108614_BombuscaroII_23	B2	64	Glomus	x
1_K607_1_KX108252_BombuscaroII_24	B2	11	Glomus	
1_K607_7_KX108239_BombuscaroII_24	B2	13	Glomus	
1_K608_1_KX108648_BombuscaroII_25b	B2	82	Acaulospora	
1_K608_2_KX108670_BombuscaroII_25b	B2	95	Acaulospora	
1_K609_1_KX108256_BombuscaroII_26g	B2	11	Glomus	
1_K610_1_KX108615_BombuscaroII_27s	B2	64	Glomus	
1_K611_1_KX108467_BombuscaroII_28	B2	17	Glomus	
1_K612_1_KX108620_BombuscaroII_28	B2	64	Glomus	
1_K612_2_KX108382_BombuscaroII_28	B2	22	Glomus	
1_K612_4_KX108683_BombuscaroII_28	B2	107	Diversispora	x
1_K613_10_KX108464_BombuscaroII_29g	B2	17	Glomus	
1_K613_12_KX108253_BombuscaroII_29g	B2	11	Glomus	

1_K614_11_KX108639_BombuscaroII_30g	B2	72	Glomus	
1_K614_12_KX108453_BombuscaroII_30g	B2	17	Glomus	
1_K614_9_KX108528_BombuscaroII_30g	B2	53	Glomus	
1_K615_4_KX108312_BombuscaroII_31	B2	21	Glomus	
1_K615_5_KX108632_BombuscaroII_31	B2	46	Glomus	
1_K616_1_KX108279_BombuscaroII_32s	B2	11	Glomus	
1_K616_2_KX108199_BombuscaroII_32s	B2	0	Glomus	
1_K617_1_KX108330_BombuscaroII_33g	B2	22	Glomus	
1_K617_2_KX108505_BombuscaroII_33g	B2	57	Glomus	
1_K617_4_KX108497_BombuscaroII_33g	B2	44	Glomus	
1_K617_8_KX108257_BombuscaroII_33g	B2	11	Glomus	
1_K618_1_KX108513_BombuscaroII_34	B2	57	Glomus	x
1_K619_2_KX108285_BombuscaroII_35	B2	11	Glomus	
1_K619_5_KX108519_BombuscaroII_35	B2	75	Glomus	x
1_K619_7_KX108581_BombuscaroII_35	B2	68	Glomus	
1_K620_1_KX108240_BombuscaroII_36	B2	13	Glomus	
1_K620_2_KX108515_BombuscaroII_36	B2	57	Glomus	
1_K620_6_KX108671_BombuscaroII_36	B2	95	Acaulospora	
1_K620_7_KX108626_BombuscaroII_36	B2	70	Glomus	x
1_K621_1_KX108627_BombuscaroII_37s	B2	70	Glomus	
1_K621_4_KX108503_BombuscaroII_37s	B2	47	Glomus	
1_K622_2_KX108436_BombuscaroII_38g	B2	17	Glomus	
1_K622_3_KX108269_BombuscaroII_38g	B2	11	Glomus	
1_K622_4_KX108318_BombuscaroII_38g	B2	21	Glomus	
1_K623_1_KX108406_BombuscaroII_39s	B2	22	Glomus	
1_K623_2_KX108406_BombuscaroII_39s	B2	17	Glomus	
1_K624_7_KX108606_BombuscaroII_39s	B2	63	Glomus	
1_K625_10_KX108656_BombuscaroII_40	B2	93	Acaulospora	
1_K626_13_KX108427_BombuscaroII_41	B2	22	Glomus	
1_K626_16_KX108692_BombuscaroII_41	B2	109	Gigaspora	
1_K626_2_KX108427_BombuscaroII_41	B2	17	Glomus	
1_K626_9_KX108582_BombuscaroII_41	B2	68	Glomus	
1_K627_10_KX108340_BombuscaroII_42	B2	22	Glomus	
1_K628_1_KX108633_BombuscaroII_43s	B2	72	Glomus	
1_K628_11_KX108685_BombuscaroII_43s	B2	107	Diversispora	
1_K628_9_KX108245_BombuscaroII_43s	B2	13	Glomus	x
1_K629_1_KX108241_BombuscaroII_44g	B2	13	Glomus	
1_K629_2_KX108526_BombuscaroII_44g	B2	75	Glomus	
1_K629_6_KX108391_BombuscaroII_44g	B2	22	Glomus	
1_K629_8_KX108258_BombuscaroII_44g	B2	11	Glomus	
1_K630_4_KX108292_BombuscaroII_45g	B2	10	Glomus	x
1_K630_6_KX108470_BombuscaroII_45g	B2	17	Glomus	
1_K630_8_KX108514_BombuscaroII_45g	B2	57	Glomus	
1_K631_4_KX108686_BombuscaroII_46	B2	107	Diversispora	
1_K632_1_KX108377_BombuscaroII_47	B2	22	Glomus	
1_K632_2_KX108273_BombuscaroII_47	B2	11	Glomus	

1_K632_8_KX108295_BombuscaroII_47	B2	10	Glomus	
1_K633_3_KX108310_BombuscaroII_48	B2	21	Glomus	
1_K633_7_KX108474_BombuscaroII_48	B2	17	Glomus	
1_K637_1_KX108286_BombuscaroII_49	B2	11	Glomus	
1_K638_11_KX108629_BombuscaroII_50	B2	70	Glomus	
1_K638_16_KX108676_BombuscaroII_50	B2	99	Acaulospora?	x
1_K638_5_KX108659_BombuscaroII_50	B2	94	Acaulospora	
1_K638_6_KX108687_BombuscaroII_50	B2	122	Archaeospora	
1_K638_8_KX108625_BombuscaroII	B2	69	Glomus	x
1_K639_2_KX108262_BombuscaroII_51s	B2	11	Glomus	
1_K639_3_KX108491_BombuscaroII_51s	B2	20	Glomus	
1_K640_1_KX108246_BombuscaroII_52g	B2	13	Glomus	
1_K640_5_KX108520_BombuscaroII_52g	B2	75	Glomus	
1_K641_1_KX108525_BombuscaroII_53	B2	75	Glomus	
1_K641_2_KX108412_BombuscaroII_53	B2	17	Glomus	
1_K642_1_KX108579_BombuscaroII_54s	B2	68	Glomus	
1_K643_11_KX108284_BombuscaroII_55	B2	11	Glomus	
1_K643_13_KX108607_BombuscaroII	B2	63	Glomus	
1_K643_4_KX108616_BombuscaroII_55	B2	64	Glomus	
1_K643_9_KX108496_BombuscaroII_55	B2	44	Glomus	x
1_K644_3_KX108270_BombuscaroII_56	B2	11	Glomus	
1_K644_4_KX108609_BombuscaroII_56	B2	63	Glomus	
1_K644_6_KX108621_BombuscaroII_56	B2	64	Glomus	
1_K646_1_KX108287_BombuscaroII_56g	B2	11	Glomus	
1_K646_3_KX108309_BombuscaroII_57g	B2	21	Glomus	
1_K647_12_KX108465_BombuscaroII_58g	B2	17	Glomus	
1_K647_2_KX108226_BombuscaroII_58g	B2	32	Glomus	
1_K647_4_KX108215_BombuscaroII_58g	B2	2	Glomus	
1_K648_1_KX108461_BombuscaroII_59g	B2	17	Glomus	
1_K648_2_KX108385_BombuscaroII_59g	B2	22	Glomus	
1_K648_3_KX108227_BombuscaroII_59g	B2	32	Glomus	
1_K649_1_KX108228_BombuscaroII_60s	B2	32	Glomus	
1_K650_3_KX108263_BombuscaroII_63	B2	11	Glomus	
1_K650_4_KX108378_BombuscaroII_63	B2	22	Glomus	
1_K650_7_KX108530_BombuscaroII_63	B2	53	Glomus	
1_K651_2_KX108247_BombuscaroII_64	B2	13	Glomus	
1_K651_3_KX108271_BombuscaroII_64	B2	11	Glomus	
1_K651_4_KX108217_BombuscaroII_64	B2	2	Glomus	
1_K651_5_KX108693_BombuscaroII_64	B2	109	Gigaspora	
1_K652_1_KX108613_BombuscaroII_66s	B2	64	Glomus	
1_K652_7_KX108611_BombuscaroII_66s	B2	63	Glomus	
1_K653_2_KX108601_BombuscaroII_67	B2	62	Glomus	
1_K653_4_KX108201_BombuscaroII_67	B2	2	Glomus	
1_K654_4_KX108617_BombuscaroII_68	B2	64	Glomus	
1_K655_1_KX108264_BombuscaroII_69g	B2	11	Glomus	x
1_K655_2_KX108416_BombuscaroII_69g	B2	17	Glomus	

1_K655_5_KX108335_BombuscaroII_69g	B2	22	Glomus	
1_K656_12_KX108417_BombuscaroII_70g	B2	17	Glomus	
1_K656_13_KX108280_BombuscaroII_70g	B2	11	Glomus	
1_K656_2_KX108605_BombuscaroII_70g	B2	62	Glomus	
1_K657_12_KX108537_BombuscaroII_71	B2	53	Glomus	
1_K657_15_KX108418_BombuscaroII_71	B2	17	Glomus	
1_K658_3_KX108388_BombuscaroII_72	B2	22	Glomus	
1_K658_5_KX108573_BombuscaroII_72	B2	54	Glomus	
1_K658_6_KX108306_BombuscaroII_72	B2	30	Glomus	
1_K658_7_KX108546_BombuscaroII_72	B2	53	Glomus	
1_K659_1_KX108401_BombuscaroI_25	B1	17	Glomus	
1_K659_2_KX108334_BombuscaroI_25	B1	22	Glomus	
1_K659_4_KX108590_BombuscaroI_25	B1	61	Glomus	
1_K661_12_KX108646_BombuscaroI_1b	B1	82	Acaulospora	
1_K661_2_KX108209_BombuscaroI_1b	B1	2	Glomus	
1_K661_7_KX108386_BombuscaroI_1b	B1	22	Glomus	
1_K661_9_KX108454_BombuscaroI_1b	B1	17	Glomus	
1_K662_10_KX108204_BombuscaroI	B1	2	Glomus	
1_K662_12_KX108636-BombuscaroI_3	B1	72	Glomus	
1_K662_14_KX108507_BombuscaroI_3	B1	57	Glomus	
1_K663_16_KX108294_BombuscaroI_10b	B1	11	Glomus	
1_K663_9_KX108498_BombuscaroI_10b	B1	44	Glomus	
1_K664_1_KX108462_BombuscaroI_15g	B1	17	Glomus	
1_K741_2_KX108229_BombuscaroI_03_14	B1	32	Glomus	
1_K742_2_KX108234_BombuscaroI_03_14	B1	32	Glomus	
1_K742_4_KX108379_BombuscaroI_03_14	B1	22	Glomus	
1_K742_5_KX108405_BombuscaroI_03_14	B1	17	Glomus	x
1_K743_2_KX108232_BombuscaroI_03_14	B1	32	Glomus	
1_K744_2_KX108336_BombuscaroI_03_14	B1	22	Glomus	
1_K744_6_KX108468_BombuscaroI_03_14	B1	17	Glomus	
1_K745_1_KX108235_BombuscaroI_03_14	B1	32	Glomus	
1_K745_2_KX108316_BombuscaroI_03_14	B1	21	Glomus	
1_K745_8_KX108442_BombuscaroI_03_14	B1	17	Glomus	
1_K747_7_KX108202_BombuscaroI_03_14	B1	2	Glomus	
1_K750_13_KX108602_BombuscaroI_03_14	B1	62	Glomus	
1_K766_5_KX108230_BombuscaroI_03_14	B1	32	Glomus	
1_K768_5_KX108542_BombuscaroI_03_14	B1	53	Glomus	
1_K768_6_KX108570_BombuscaroI_03_14	B1	54	Glomus	
1_K768_7_KX108554_BombuscaroI_03_14	B1	50	Glomus	
1_K769_3_KX108529_BombuscaroI_03_14	B1	53	Glomus	
1_K770_4_KX108538_BombuscaroI_03_14	B1	53	Glomus	
1_K770_7_KX108429_BombuscaroI_03_14	B1	17	Glomus	
1_K770_8_KX108559_BombuscaroI_03_14	B1	50	Glomus	x
1_K772_1_KX108689_BombuscaroI_03_14	B1	131	Archaeospora	
1_K774_2_KX108550_BombuscaroI_03_14	B1	53	Glomus	
1_K774_7_KX108550_BombuscaroI_03_14	B1	22	Glomus	

1_K774_8_KX108400_BombuscaroI_03_14	B1	17	Glomus	
1_K776_4_KX108539_BombuscaroI_03_14	B1	53	Glomus	
1_K776_8_KX108494_BombuscaroI_03_14	B1	20	Glomus	
1_K777_1_KX108238_BombuscaroI_03_14	B1	2	Glomus	
1_K777_5_KX108313_BombuscaroI_03_14	B1	21	Glomus	
1_K777_8_KX108220_BombuscaroI_03_14	B1	3	Glomus	
1_K778_9_KX108244_BombuscaroI_03_14	B1	13	Glomus	
1_K783_5_KX108231_BombuscaroI_03_14	B1	32	Glomus	
1_K784_1_KX108218_BombuscaroI_03_14	B1	2	Glomus	
1_K784_3_KX108233_BombuscaroI_03_14	B1	32	Glomus	
1_K786_1_KX108521_BombuscaroI_03_14	B1	75	Glomus	
1_K788_1_KX108493_BombuscaroI_03_14	B1	20	Glomus	x
1_K788_2_KX108288_BombuscaroI_03_14	B1	11	Glomus	
1_K788_4_KX108657_BombuscaroI_03_14	B1	93	Acaulospora	
1_K788_8_KX108344_BombuscaroI_03_14	B1	22	Glomus	
1_K790_1_KX108524_BombuscaroII_03_14	B2	75	Glomus	
1_K790_7_KX108237_BombuscaroII	B2	4	Glomus	x
1_K791_1_KX108492_BombuscaroII_03_14	B2	20	Glomus	
1_K791_2_KX108443_BombuscaroII_03_14	B2	17	Glomus	
1_K791_3_KX108583_BombuscaroII_03_14	B2	68	Glomus	
1_K791_4_KX108669_BombuscaroII_03_14	B2	95	Acaulospora	
1_K791_5_KX108500_BombuscaroII_03_14	B2	44	Glomus	
1_K792_1_KX108492_BombuscaroII_03_14	B2	53	Glomus	
1_K792_2_KX108555_BombuscaroII_03_14	B2	50	Glomus	
1_K794_1_KX108510_BombuscaroII_03_14	B2	57	Glomus	
1_K795_1_KX108640_BombuscaroII_03_14	B2	72	Glomus	
1_K795_7_KX108248_BombuscaroII_03_14	B2	13	Glomus	
1_K797_5_KX108242_BombuscaroII_03_14	B2	13	Glomus	
1_K799_1_KX108249_BombuscaroII_03_14	B2	13	Glomus	
1_K799_5_KX108222_BombuscaroII_03_14	B2	3	Glomus	
1_K802_3_KX108243_BombuscaroII_03_14	B2	13	Glomus	
1_K803_2_KX108430_BombuscaroIII_03_14	B3	17	Glomus	
1_K803_3_KX108302_BombuscaroIII_03_14	B3	29	Glomus	
1_K803_6_KX108561_BombuscaroIII_03_14	B3	50	Glomus	
1_K804_1_KX108399_BombuscaroIII_03_14	B3	17	Glomus	
1_K805_1_KX108543_BombuscaroIII_03_14	B3	53	Glomus	x
1_K805_2_KX108558_BombuscaroIII_03_14	B3	50	Glomus	
1_K807_1_KX108283_BombuscaroIII_03_14	B3	11	Glomus	
1_K807_2_KX108314_BombuscaroIII_03_14	B3	21	Glomus	
1_K807_4_KX108431_BombuscaroIII_03_14	B3	17	Glomus	
1_K808_1_KX108275_BombuscaroIII_03_14	B3	11	Glomus	
1_K808_4_KX108390_BombuscaroIII_03_14	B3	22	Glomus	
1_K810_2_KX108274_BombuscaroIII_03_14	B3	11	Glomus	
1_K820_3_KX108591_BombuscaroIII_03_14	B3	61	Glomus	
1_K821_3_KX108278_BombuscaroIII_03_14	B3	11	Glomus	
1_K822_2_KX108596_BombuscaroIII_03_14	B3	61	Glomus	

1_K822_3_KX108337_BombuscaroIII_03_14	B3	22	Glomus	
1_K823_2_KX108672_BombuscaroIII_03_14	B3	95	Acaulospora	
1_K825_1_KX108677_BombuscaroIII_03_14	B3	100	Acaulospora?	
1_K825_2_KX108489_BombuscaroIII_03_14	B3	20	Glomus	
1_K825_3_KX108434_BombuscaroIII_03_14	B3	17	Glomus	
1_K826_1_KX108303_BombuscaroIII_03_14	B3	29	Glomus	
1_K827_1_KX108304_BombuscaroIII_03_14	B3	29	Glomus	
1_K827_2_KX108319_BombuscaroIII_03_14	B3	21	Glomus	
1_K828_1_KX108301_BombuscaroIII_03_14	B3	29	Glomus	
1_K828_2_KX108531_BombuscaroIII_03_14	B3	53	Glomus	
1_K828_6_KX108291_BombuscaroIII_03_14	B3	10	Glomus	
1_K828_7_KX108297_BombuscaroIII_03_14	B3	12	Glomus	x
1_K829_2_KX108293_BombuscaroIII_03_14	B3	10	Glomus	
1_K829_3_KX108556_BombuscaroIII_03_14	B3	50	Glomus	
1_K829_5_KX108338_BombuscaroIII_03_14	B3	22	Glomus	
1_K829_6_KX108549_BombuscaroIII_03_14	B3	53	Glomus	
1_K830_5_KX108438_BombuscaroIII_03_14	B3	17	Glomus	
1_K830_7_KX108691_BombuscaroIII_03_14	B3	109	Gigaspora	x
1_K831_3_KX108359_BombuscaroIII_03_14	B3	22	Glomus	
1_K831_4_KX108577_BombuscaroIII_03_14	B3	68	Glomus	
1_K832_15_KX108544_BombuscaroIII_03_14	B3	53	Glomus	
1_K833_3_KX108578_BombuscaroIII_03_14	B3	68	Glomus	
1_K834_1_KX108361_BombuscaroIII_03_14	B3	22	Glomus	
1_K834_3_KX108300_BombuscaroIII_03_14	B3	29	Glomus	
1_K834_4_KX108300_BombuscaroIII_03_14	B3	17	Glomus	
1_K835_1_KX108339_BombuscaroIII_03_14	B3	22	Glomus	
1_K835_5_KX108451_BombuscaroIII_03_14	B3	17	Glomus	
1_K836_1_KX108236_BombuscaroIII	B3	4	Glomus	
1_K836_2_KX108522_BombuscaroIII_03_14	B3	75	Glomus	
1_K837_1_KX108197_BombuscaroIII_03_14	B3	1	Glomus	
1_K837_7_KX108445_BombuscaroIII_03_14	B3	17	Glomus	
1_K838_2_KX108523_BombuscaroIII_03	B3	75	Glomus	
1_K838_5_KX108211_BombuscaroIII_03_14	B3	2	Glomus	
1_K838_7_KX108223_BombuscaroIII_03_14	B3	3	Glomus	x
1_K839_1_KX108317_BombuscaroIII_03_14	B3	21	Glomus	
1_K839_4_KX108572_BombuscaroIII_03_14	B3	54	Glomus	
1_K839_5_KX108410_BombuscaroIII_03_14	B3	17	Glomus	
1_K840_2_KX108694_BombuscaroIII_03_14	B3	109	Gigaspora	
1_K840_3_KX108370_BombuscaroIII_03_14	B3	22	Glomus	
1_K840_5_KX108440_BombuscaroIII_03_14	B3	17	Glomus	
2_F_EF447220_K1c3_Glomus_Alzatea_VTX191	T2	43	Glomus	
2_F_EF447223_K3c6_F_T2_Alzatea	T2	52	Glomus	
2_F_EF447226_K7c5_F_Alzatea	T2	43	Glomus	
2_F_EF447230_K8c4_F_T2_Alzatea	T2	49	Glomus	
2_F_EF447234_K11c8_F_T2_Alzatea	T2	71	Glomus	
2_F_EF447237_7.1.3b_F_T2_Alzatea	T2	16	Glomus	

2_F_EF447238_8.2.10_F_T2_Alzatea	T2	44	Glomus	
2_F_EF447239_K10c4_F_T2_Alzatea	T2	82	Acaulospora	
2_F_EF447241_K15c1_F_T2	T2	82	Acaulospora	
2_F_EF447242_K15c2_F_T2_Alzatea	T2	109	Gigaspora	
2_F_EF447243_K16c1_F_T2_Alzatea	T2	80	Acaulospora	
2_F_EF447244_5.4.1_F_T2_Alzatea	T2	80	Acaulospora	
2_F_EF447245_7.1.3_F_T2_Alzatea	T2	80	Acaulospora	
2_F_K1_1_JX2969181_Fp_Q5_Cedrela1	Q5	47	Glomus	
2_F_K1_12_JX296943_Fp_Q5_Cedrela1	Q5	68	Glomus	
2_F_K1_3_JX296974_Fp_Cedrela1	Q5	66	Glomus	
2_F_K1_5_JX296690_Fp_Q5_Cedrela1	Q5	1	Glomus	
2_F_K106_2_JX296959_Fp_Plant15	Q5	64	Glomus	
2_F_K106_4_JX296954_F_Q5_Plant15	Q5	68	Glomus	
2_F_K107_5_JX296983_F_Plant16	Q5	66	Glomus	
2_F_K107_8_JX296685_F_Q5_Plant16	Q5	0	Glomus	x
2_F_K108_3_KX108120_F_Q5_Roots	Q5	72	Glomus	
2_F_K109_1_JX296830_F_Q5_Plant17	Q5	21	Glomus	
2_F_K109_11_JX297117_F_Q5_Plant17	Q5	130	Archaeospora	
2_F_K109_16_JX297093_F_Q5_Plant17	Q5	122	Archaeospora	
2_F_K109_3_JX297091_F_Q5_Plant17	Q5	110	Scutellospora	x
2_F_K109_8_JX297056_F_Q5_Plant17	Q5	88	Acaulospora	
2_F_K114_1_JX296821_K114_1_F_Q5_Plant18	Q5	21	Glomus	
2_F_K12_2_JX296714_Fp_Q5_Cedrela5	Q5	1	Glomus	
2_F_K12_3_JX296945_Fp_Cedrela5	Q5	68	Glomus	
2_F_K127_1_JX297072_F_Q5_Plant19	Q5	93	Acaulospora	
2_F_K127_12_JX296980_F_Plant19	Q5	66	Glomus	
2_F_K127_15_JX296951_F_Plant19	Q5	68	Glomus	
2_F_K127_4_JX296748_F_Q5_Plant19	Q5	2	Glomus	
2_F_K128_28_DQ336488_F_Neill_Guarea	T2	33	Glomus	x
2_F_K128_29_DQ336489_F_Neill_Guarea_p	T2	5	Glomus	
2_F_K128n_10_JX297017_F_Q5	Q5	79	Funneliformis	
2_F_K128n_11_JX296929_F_Q5_Plant20	Q5	56	Glomus	x
2_F_K128n_12_JX297009_F_Q5	Q5	72	Glomus	x
2_F_K128n_7_JX297012_F_Q5	Q5	73	Glomus	
2_F_K129_14_DQ336490_F_Q2_Guarea_sp	Q2	47	Glomus	
2_F_K129_22_EU152132_F_Q2_Guarea_sp	Q2	72	Glomus	
2_F_K130_14_DQ336482_F_Q2_Guarea_k	Q2	0	Glomus	
2_F_K131_11_DQ336485_F_Guarea_cf_k	T2	67	Glomus	
2_F_K132_10_DQ336487_F_Q2_Guarea	Q2	47	Glomus	
2_F_K133_1_DQ336483_F_Q2_Guarea_k	Q2	29	Glomus	
2_F_K142_4_DQ336499_F_T2_Hyeronima_m	T2	49	Glomus	
2_F_K142_9_DQ336500_F_T2_Hyeronima_m	T2	71	Glomus	
2_F_K143_13_DQ336496_F_A_Hyeronima_a	T2	21	Glomus	x
2_F_K143_17_DQ336497_F_A_Hyeronima_a	T2	29	Glomus	
2_F_K144_10_DQ336504_F_A_Hyeronima	T2	47	Glomus	
2_F_K144_2_EU152134_F_A_Hyeronima_sp	T2	41	Glomus	

2_F_K146_5_DQ336502_F_Q2_Hyeronima_o	Q2	0	Glomus	
2_F_K147_21_DQ336476_F_A_Graffenrieda1	T2	16	Glomus	
2_F_K147_22_EU152135_F_T2_Graffenrieda1	T2	15	Glomus	
2_F_K149_4_DQ336478_F_T2_Graffenrieda2	T2	71	Glomus	
2_F_K149_5_DQ336479_F_T2_Graffenrieda2	T2	49	Glomus	
2_F_K150_1_DQ336480_F_A_Graffenrieda3	T2	49	Glomus	
2_F_K150_3_DQ336481_F_A_Graffenrieda3	T2	71	Glomus	
2_F_K151_2_JX297055_Fp_Q5_Tabebuia16	Q5	88	Acaulospora	
2_F_K151_3_JX296986_Fp_Tabebuia16	Q5	66	Glomus	
2_F_K152_1_JX2970071_Fp_Tabebuia17	Q5	66	Glomus	
2_F_K152_10_JX296759_Fp_Q5_Tabebuia17	Q5	29	Glomus	
2_F_K152_2_JX296952_Fp_Q5_Tabebuia17	Q5	68	Glomus	
2_F_K152_3_JX297058_Fp_Q5_Tabebuia17	Q5	88	Acaulospora	
2_F_K152_6_JX297025_Fp_Q5_Tabebuia17	Q5	93	Acaulospora	
2_F_K153_10_JX297053_Fp_Q5_Tabebuia18	Q5	88	Acaulospora	
2_F_K153_5_JX296950_Fp_Tabebuia18	Q5	68	Glomus	
2_F_K153_6_JX296995_Fp_Tabebuia18	Q5	66	Glomus	
2_F_K159_3_JX296827_Fn_Q5_Tabebuia22	Q5	21	Glomus	
2_F_K162_1_JX296926_Fn_Q5_Tabebuia23	Q5	56	Glomus	
2_F_K162_4_JX296836_Fn_Q5_Tabebuia23	Q5	21	Glomus	
2_F_K164_4_KX108188_FW	T2	109	Gigaspora	
2_F_K167_2_DQ336466_F_Q5_Cedrela61	Q5	1	Glomus	
2_F_K168_7_DQ336471_F_A_Clethra	T2	31	Glomus	
2_F_K168n_1_KX108186_FW	T2	128	Archaeospora	
2_F_K168n_5_KX108183_FW	T2	127	Archaeospora	
2_F_K17_6_JX297016_F_Q5_Cedrela14	Q5	79	Funneliformis	x
2_F_K17_7_JX297010_F_Q5_Cedrela14	Q5	72	Glomus	
2_F_K170_7_DQ336508_F_Q2_Inga3	Q2	68	Glomus	
2_F_K171_6_DQ336493_F_Q5_Heliocarpus11	Q5	31	Glomus	x
2_F_K172_10_JX296779_F_T1u_Plant4	T1	16	Glomus	
2_F_K172_11_JX296914_T1u_Plant4	T1	52	Glomus	
2_F_K172_12_JX297090_F_T1u_Plant4	T1	113	Scutellospora	
2_F_K172_14_JX297061_F_T1u_Plant4	T1	80	Acaulospora	
2_F_K173_2_DQ336522_F_A_Vismia	T2	16	Glomus	
2_F_K173_4_DQ336523_F_A_Vismia	T2	47	Glomus	
2_F_K173n_4_JX296761_F_T1u	T1	14	Glomus	
2_F_K174_2_DQ336524_F_Vismia	T2	68	Glomus	
2_F_K174n_1_JX296913_F_T1u_Plant6	T1	52	Glomus	
2_F_K174n_3_JX296893_F_T1u_Plant6	T1	49	Glomus	
2_F_K174n_6_JX296939_F_T1u_Plant6	T1	71	Glomus	
2_F_K175_7_DQ336514_F_Q5_Nectandra6	Q5	68	Glomus	
2_F_K180n_5_KX108189_FW	T2	109	Gigaspora	
2_F_K181_2_JX296883_T1o_Plant7	T1	45	Glomus	x
2_F_K181_5_JX296975_F_T1o_Plant7	T1	66	Glomus	
2_F_K181_8_JX296890_F_T1o_Plant7	T1	44	Glomus	
2_F_K181_9_JX296898_F_T1o_Plant7	T1	49	Glomus	

2_F_K182_1_JX296891_F_T1o	T1	44	Glomus	
2_F_K182_9_JX296884_F_T1o	T1	45	Glomus	
2_F_K183_4_JX296857_F_T1o_Plant9	T1	36	Glomus	
2_F_K184_15_JX297059_Fp_Q5_Tabebuia	Q5	87	Acaulospora	
2_F_K184_2_JX296887_Fp_Tabebuia	Q5	44	Glomus	
2_F_K184_3_JX296803_Fp_Q5_Tabebuia	Q5	30	Glomus	
2_F_K184_5_JX297100_Fp_Q5_Tabebuia	Q5	122	Archaeospora	
2_F_K184_6_JX296797_Fp_Q5_Tabebuia	Q5	29	Glomus	
2_F_K185n_1_JX297095_Fp_Q5_Nectandra1	Q5	122	Archaeospora	
2_F_K185n_12_JX297106_Fp_Q5_Nectandra1	Q5	126	Archaeospora	
2_F_K185n_3_JX296969_Fp_Nectandra1	Q5	67	Glomus	
2_F_K185n_5_JX297044_Fp_Q5_Nectandra1	Q5	95	Acaulospora	
2_F_K186n_1_JX296722_Fp_Q5_Nectandra2	Q5	1	Glomus	
2_F_K187n_2_JX296948_Fp_Q5_Tabebuia26	Q5	68	Glomus	
2_F_K187n_5_JX297015_Fp_Q5_Tabebuia26	Q5	78	Funneliformis	x
2_F_K187n_7_JX297118_Fp_Q5_Tabebuia26	Q5	130	Archaeospora	
2_F_K188_2_JX297109_Fp_Q5_Tabebuia27	Q5	127	Archaeospora	
2_F_K188_3_JX296804_Fp_Q5_Tabebuia27	Q5	30	Glomus	
2_F_K188_5_JX296832_Fp_Q5_Tabebuia27	Q5	21	Glomus	
2_F_K189_4_DQ336507_F_Inga2	T2	67	Glomus	x
2_F_K189n_3_JX296978_Fp_Nectandra3	Q5	66	Glomus	x
2_F_K190_1_DQ336505_F_Inga1	T2	66	Glomus	
2_F_K190n_4_JX296807_Fp_Q5_Nectandra4	Q5	30	Glomus	
2_F_K192_1_JX297079_Fp_Q5_Nectandra5	Q5	114	Claroideoglomus	
2_F_K192_6_JX296809_Fp_Q5_Nectandra5	Q5	30	Glomus	
2_F_K192_7_JX296723_Fp_Q5_Nectandra5	Q5	1	Glomus	
2_F_K207n_2_KX108190_FW	T2	109	Gigaspora	
2_F_K22_1_JX297088_Fp_Q5_Cedrela19	Q5	108	Gigaspora	x
2_F_K22_10_JX296742_Fp_Q5_Cedrela19	Q5	5	Glomus	
2_F_K22_11_JX296791_Fp_Q5_Cedrela19	Q5	29	Glomus	
2_F_K22_2_JX296801_Fp_Q5_Cedrela19	Q5	30	Glomus	
2_F_K22_3_JX296724_Fp_Q5_Cedrela19	Q5	1	Glomus	
2_F_K22_5_JX297103_Fp_Q5_Cedrela19	Q5	126	Archaeospora	
2_F_K228_2_DQ336516_F_Q5_Nectandra7	Q5	0	Glomus	
2_F_K228n_1_KX108153_Fp_Q5_Tabebuia	Q5	93	Acaulospora	
2_F_K228n_4_KX108161_Fp_Q5_Tabebuia	Q5	95	Acaulospora	
2_F_K229_2_DQ336474_F_Q3_Clusia_e	Q2	49	Glomus	
2_F_K23_1_JX296733_Fp_Q5_Cedrela20	Q5	1	Glomus	
2_F_K23_11_JX2971041_Fp_Q5_Cedrela20	Q5	126	Archaeospora	
2_F_K23_4_JX296968_Fp_Cedrela20	Q5	67	Glomus	
2_F_K23_5_JX296799_Fp_Q5_Cedrela20	Q5	30	Glomus	
2_F_K23_6_JX296923_Fp_Q5_Cedrela20	Q5	56	Glomus	
2_F_K23_7_JX296798_Fp_Q5_Cedrela20	Q5	29	Glomus	
2_F_K23_9_JX297081_Fp_Q5_Cedrela20	Q5	114	Claroideoglomus	x
2_F_K230_5_DQ336521_F_T2_Podocarpus1	T2	16	Glomus	
2_F_K232_1_EU152144_F_T2_Prumnopytis	T2	5	Glomus	x

2_F_K232_8_EU152145_F_T2_Prumnopytis	T2	7	Glomus	x
2_F_K235_23_EU152146_F_T2_Podocarpus3	T2	16	Glomus	
2_F_K24_1_JX297020_Fn_Q5_Cedrela21	Q5	93	Acaulospora	
2_F_K24_12_JX296741_Fn_Q5_Cedrela21	Q5	5	Glomus	
2_F_K24_13_JX297054_Fn_Q5_Cedrela21	Q5	88	Acaulospora	
2_F_K24_15_JX296924_Fn_Q5_Cedrela21	Q5	56	Glomus	
2_F_K24_3_KX108169_Fn_Q5_Cedrela21	Q5	96	Acaulospora	x
2_F_K24_4_JX297027_Fn_Q5_Cedrela21	Q5	95	Acaulospora	
2_F_K24_6_JX297113_Fn_Q5_Cedrela21	Q5	131	Archaeospora	
2_F_K24_7_JX296734_Fn_Q5_Cedrela21	Q5	1	Glomus	
2_F_K24_9_JX297108_Fn_Q5_Cedrela21	Q5	126	Archaeospora	
2_F_K241_14_KX108165_Fn_Q5_Cedrela	Q5	94	Acaulospora	
2_F_K241_3_KX107977_Fn_Q5_Cedrela	Q5	26	Glomus	
2_F_K243_1_KX108102_F_Q5_Hyeronima	Q5	66	Glomus	
2_F_K25_5_JX296947_Fn_Cedrela22	Q5	68	Glomus	
2_F_K25_6_JX296725_Fn_Q5_Cedrela22	Q5	1	Glomus	
2_F_K25_9_JX297082_Fn_Q5_Cedrela22	Q5	117	Claroideoglomus	
2_F_K252_1_EU152150_F_T2_Alzatea	T2	49	Glomus	
2_F_K253_1_EU152151_F_Q2_Campnosperma	Q2	14	Glomus	x
2_F_K253_7_EU152152_F_Q2_Campnosperma	Q2	16	Glomus	
2_F_K255_3_EU152153_F_T2_Podocarpus2	T2	15	Glomus	x
2_F_K257_3_EU152154_F_Q2_Faramea	Q2	14	Glomus	
2_F_K257_5_EU152155_F_Q2_Faramea	Q2	47	Glomus	x
2_F_K258_4_EU152156_F_A_Critoniopsis	T2	47	Glomus	
2_F_K259_1_EU152157_F_Q2_Miconia	Q2	47	Glomus	
2_F_K26_1_JX296727_F_Tom_Clusia_sp	T2	1	Glomus	
2_F_K26_4_JX297066_F_Tom_Clusia_sp	T2	82	Acaulospora	
2_F_K260_3_EU152158_F_T2_Prunus	T2	41	Glomus	
2_F_K260_7_EU152159_F_T2_Prunus_cf_o	T2	16	Glomus	
2_F_K262_2_KX108148_F_Q5_Baum	Q5	82	Acaulospora	
2_F_K262_3_KX108176_F_Q5	Q5	122	Archaeospora	
2_F_K262_4_KX108173_F_Q5	Q5	114	Claroideoglomus	
2_F_K262_7_KX108122_F_Q5_Baum00025	Q5	79	Funneliformis	
2_F_K280n_2_KX107920_Fp_Q5_Cedrela	Q5	2	Glomus	
2_F_K280n_4_KX108106_Fp_Cedrela	Q5	64	Glomus	
2_F_K280n_6_KX108045_Fp_Cedrela	Q5	63	Glomus	
2_F_K280n_7_KX108045_Fp_Q5_Cedrela	Q5	55	Glomus	
2_F_K281n_1_KX107900_Fp_Q5_Cedrela	Q5	55	Glomus	
2_F_K281n_5_KX108184_Fp_Q5_Cedrela	Q5	127	Archaeospora	x
2_F_K281n_6_KX107935_Fp_Q5_Cedrela	Q5	21	Glomus	
2_F_K282n_1_KX107936_Fp_Q5_Cedrela	Q5	21	Glomus	
2_F_K284n_1_KX108174_Fn_Q5_Cedrela	Q5	117	Claroideoglomus	
2_F_K284n_8_KX108149_Fn_Q5_Cedrela	Q5	88	Acaulospora	
2_F_K285n_2_KX107914_Fn_Q5_Cedrela	Q5	5	Glomus	
2_F_K29_10_JX296696_F_Q5_Cedrela24	Q5	1	Glomus	x
2_F_K29_4_JX297083_F_Q5_Cedrela24	Q5	109	Gigaspora	

2_F_K298n_1_KX108178_Fn_Q5_Cedrela	Q5	122	Archaeospora	x
2_F_K298n_2_KX107929_Fn_Q5_Cedrela	Q5	29	Glomus	
2_F_K298n_5_KX108179_Fn_Q5_Cedrela	Q5	124	Archaeospora	x
2_F_K31_9_JX296716_Fn_Q5_Cedrela	Q5	1	Glomus	
2_F_K314n_1_KX108150_Fp_Q5_Tabebuia	Q5	88	Acaulospora	
2_F_K315_1_KX108158_Fp_Q5_Tabebuia	Q5	95	Acaulospora	
2_F_K315_2_KX108181_Fp_Q5_Tabebuia	Q5	126	Archaeospora	x
2_F_K319_1_KX108194_F_T2_Graffenrieda1	T2	113	Scutellospora	x
2_F_K319_7_KX107945_F_T2_Graffenrieda1	T2	16	Glomus	
2_F_K32_10_JX296953_Fn_Q5_Cedrela27	Q5	68	Glomus	x
2_F_K32_11_JX296966_Fn_Cedrela27	Q5	67	Glomus	
2_F_K32_5_JX297022_Fn_Q5_Cedrela27	Q5	93	Acaulospora	
2_F_K32_6_JX297092_Fn_Q5_Cedrela27	Q5	122	Archaeospora	
2_F_K32_9_JX296717_Fn_Q5_Cedrela27	Q5	1	Glomus	
2_F_K320_1_KX108089_F_T2_Graffenrieda	T2	68	Glomus	
2_F_K320_8_KX108163_F_T2_Graffenrieda2	T2	94	Acaulospora	
2_F_K33_11_JX296758_Fp_Q5_Cedrela28	Q5	7	Glomus	
2_F_K331_9_KX108078_F_Q5_Piper	Q5	54	Glomus	
2_F_K334_2_KX107960_F_Q5_Wurzeln	Q5	15	Glomus	
2_F_K334_5_KX108090_F_Q5	Q5	68	Glomus	
2_F_K334_7_KX108121_F_Q5_Plant	Q5	72	Glomus	
2_F_K35_1_AY394664_F_T2_Graffenrieda2	T2	95	Acaulospora	
2_F_K367_3_KX108160_Fp_Q5_Tabebuia	Q5	95	Acaulospora	
2_F_K37_10_JX296946_F_Q5_Plant21	Q5	68	Glomus	
2_F_K37_12_JX297114_F_Q5_Plant21	Q5	131	Archaeospora	
2_F_K37_13_JX296916_F_Q5_Plant21	Q5	47	Glomus	
2_F_K37_16_JX297077_F_Q5_Plant21	Q5	114	Claroideoglomus	
2_F_K38_11_JX296915_F_Q5_Plant22	Q5	47	Glomus	
2_F_K38_13_JX296958_Fp_Plant22	Q5	64	Glomus	
2_F_K38_14_JX297024_F_Q5_Plant22	Q5	93	Acaulospora	
2_F_K39_1_JX297052_Fp_Q5_Plant28	Q5	88	Acaulospora	x
2_F_K39_11_JX296982_F_Q5_Plant28	Q5	66	Glomus	
2_F_K39_2_JX297032_F_Q5_Plant28	Q5	95	Acaulospora	
2_F_K399_1_KX107972_F_T2_2550m_Graffenrieda	T2	26	Glomus	x
2_F_K399_3_KX107944_F_T2_2550m_Graffenrieda	T2	16	Glomus	
2_F_K399_6_KX108151_F_T2_2550m_Graffenrieda	T2	91	Acaulospora	
2_F_K400_2_KX108145_F_T2_2550m_Graffenrieda	T2	83	Acaulospora	x
2_F_K423_4_KX108024_F_T2_1250	T2	41	Glomus	
2_F_K427_1_KX108025_F_T2_1250	T2	41	Glomus	
2_F_K427_4_KX107949_F_T2	T2	16	Glomus	
2_F_K43_10_JX297075_Fn_Q5_Cedrela33	Q5	105	Diversispora	x
2_F_K43_12_JX297105_Fn_Q5_Cedrela33	Q5	126	Archaeospora	
2_F_K43_8_JX296965_Fn_Cedrela33	Q5	67	Glomus	
2_F_K438_5_KX108113_F_T2_2550m	T2	71	Glomus	
2_F_K440_2_KX108023_F_T2_2550m	T2	41	Glomus	
2_F_K440_8_KX108084_F_T2_2550m	T2	52	Glomus	

2_F_K443_1_KX108068_F_T2_2500m	T2	49	Glomus	
2_F_K443_6_F_KX108137_T2_2550m	T2	82	Acaulospora	
2_F_K444_1_KX108111_F_T2_2550m	T2	71	Glomus	
2_F_K447_1_KX108109_F_T2_1250	T2	71	Glomus	
2_F_K448_1_KX108057_F_T2_950_Plant	T2	60	Glomus	
2_F_K448_5_KX108060_F_T2_950	T2	59	Glomus	x
2_F_K448_8_KX107911_F_T2_950	T2	1	Glomus	
2_F_K45_1_JX297028_Fp_Q5_Cedrela35	Q5	95	Acaulospora	
2_F_K45_2_JX297049_Fp_Q5_Cedrela35	Q5	87	Acaulospora	
2_F_K45_3_JX296751_Fp_Q5_Cedrela35	Q5	2	Glomus	
2_F_K450_1_KX108036_F_Graffenrieda_T2	T2	43	Glomus	
2_F_K450_3_KX108079_F_T2_950_Graffenrieda	T2	52	Glomus	
2_F_K450_4_KX108114_F_T2_950_Graffenrieda	T2	71	Glomus	
2_F_K451_4_F_KX108126_T2_2550m_Podocarpus	T2	80	Acaulospora	
2_F_K452_1_F_KX107951_T2_2550m_Graffenrieda	T2	16	Glomus	
2_F_K455_2_KX108080_F_T2_2550m_Plant	T2	52	Glomus	
2_F_K455_8_KX108110_T2_2500m	T2	71	Glomus	
2_F_K458_1_KX108125_F_T2_2550m_Plant	T2	80	Acaulospora	
2_F_K459_2_KX108140_F_T2_2550m_Clusia	T2	82	Acaulospora	
2_F_K460_1_KX108081_F_T2_2550m_Graffenrieda	T2	52	Glomus	
2_F_K53_3_JX296750_F_Q5_Plant24	Q5	2	Glomus	
2_F_K53_7_JX296967_F_Q5_Plant24	Q5	67	Glomus	
2_F_K665_1_KX108175_T2_1250	T2	117	Claroideoglomus	x
2_F_K666_1_KX108026_T2_1250	T2	44	Glomus	
2_F_K667_8_KX108132_T2_1250	T2	80	Acaulospora	
2_F_K669_7_KX108142_T2_1250	T2	82	Acaulospora	
2_F_K670_8_KX108124_T2_1250	T2	80	Acaulospora	
2_F_K670_9_KX108134_T2_1250	T2	82	Acaulospora	
2_F_K671_1_KX108138_T2_1250	T2	82	Acaulospora	
2_F_K671_2_KX108074_T2_1250	T2	49	Glomus	
2_F_K674_4_KX108141_T2_1250	T2	82	Acaulospora	
2_F_K676_2_KX108143_T2_1250	T2	82	Acaulospora	
2_F_K677_10_KX108082_T2_950	T2	52	Glomus	
2_F_K677_2_KX107958_T2_950	T2	16	Glomus	
2_F_K677_9_KX108077_T2_950	T2	49	Glomus	
2_F_K678_11_KX108020_T2_950	T2	41	Glomus	
2_F_K678_4_KX108027_T2_950	T2	44	Glomus	
2_F_K678_9_KX108001_T2_950	T2	36	Glomus	
2_F_K679_10_KX108021_T2_950	T2	41	Glomus	
2_F_K679_7_KX107968_T2_950	T2	25	Glomus	
2_F_K680_1_KX107969_T2_950	T2	25	Glomus	
2_F_K680_2_KX107950_T2_950	T2	16	Glomus	
2_F_K681_5_KX108018_T2_950	T2	41	Glomus	
2_F_K681_8_KX108072_T2_950	T2	49	Glomus	
2_F_K682_1_KX107942_T2_950	T2	16	Glomus	
2_F_K682_2_KX108071_T2_950	T2	49	Glomus	

2_F_K683_6_KX107909_T2_950	T2	1	Glomus	
2_F_K684_2_KX107908_T2_950	T2	1	Glomus	
2_F_K688_1_KX107902_T2_950	T2	0	Glomus	
2_F_K690_1_KX108061_T2_950	T2	59	Glomus	
2_F_K690_5_KX108058_T2_950	T2	60	Glomus	x
2_F_K691_6_KX108013_T2_950	T2	35	Glomus	x
2_F_K692_2_KX107953_T2_950	T2	16	Glomus	
2_F_K702_1_KX108073_T2_950	T2	49	Glomus	
2_F_K702_2_KX108085_T2_950	T2	52	Glomus	
2_F_K703_1_KX108135_T2_950	T2	82	Acaulospora	
2_F_K704_2_KX107957_T2_950	T2	16	Glomus	
2_F_K704_5_KX108123_T2_950	T2	80	Acaulospora	
2_F_K705_1_KX108066_T2_950	T2	49	Glomus	
2_F_K705_2_KX108115_T2_950	T2	71	Glomus	x
2_F_K707_4_KX108029_T2_550	T2	43	Glomus	
2_F_K708_1_KX108070_T2_550	T2	49	Glomus	
2_F_K708_5_KX107954_T2_550	T2	16	Glomus	
2_F_K710_1_KX108139_T2_550	T2	82	Acaulospora	
2_F_K710_2_KX108129_T2_550	T2	80	Acaulospora	x
2_F_K710_7_KX107946_T2_550	T2	16	Glomus	
2_F_K711_1_KX108076_T2_550	T2	49	Glomus	
2_F_K711_2_KX108130_T2_550	T2	80	Acaulospora	
2_F_K711_3_KX108086_T2_550	T2	52	Glomus	x
2_F_K712_2_KX108019_T2_550	T2	41	Glomus	
2_F_K713_1_KX108035_T2_550	T2	43	Glomus	
2_F_K714_1_KX108032_T2_550	T2	43	Glomus	
2_F_K715_5_KX108136_T2_550	T2	82	Acaulospora	
2_F_K716_1_KX108030_T2_550	T2	43	Glomus	
2_F_K717_3_KX108034_T2_550	T2	43	Glomus	
2_F_K721_1_KX108112_T2_550	T2	71	Glomus	
2_F_K721_3_KX108075_T2_550	T2	49	Glomus	
2_F_K722_2_KX108028_T2_550	T2	44	Glomus	
2_F_K722_3_KX108083_T2_550	T2	52	Glomus	
2_F_K723_3_KX108031_T2_550	T2	43	Glomus	
2_F_K724_2_KX107906_T2_250	T2	1	Glomus	
2_F_K725_8_KX108156_T2_250	T2	95	Acaulospora	
2_F_K726_2_KX108166_T2_250	T2	95	Acaulospora	
2_F_K727_8_KX107956_T2_250	T2	16	Glomus	
2_F_K728_1_KX108159_T2_250	T2	95	Acaulospora	x
2_F_K730_3_KX108067_T2_250	T2	49	Glomus	
2_F_K730_8_KX107955_T2_250	T2	16	Glomus	
2_F_K731_4_KX107943_T2_250_Graffenrieda	T2	16	Glomus	
2_F_K732_1_KX107952_T2_250	T2	16	Glomus	x
2_F_K733_6_KX108033_T2_250	T2	43	Glomus	
2_F_K734_1_KX107959_T2_250	T2	16	Glomus	
2_F_K736_2_KX108162_T2_250	T2	95	Acaulospora	

2_F_K78_1_JX297008_Fn_Q5_Cedrela50	Q5	72	Glomus	
2_F_K78_10_JX296917_Fn_Q5_Cedrela50	Q5	47	Glomus	
2_F_K78_11_JX297001_Fn_Cedrela50	Q5	66	Glomus	
2_F_K78_13_JX297111_Fn_Q5_Cedrela50	Q5	128	Archaeospora	
2_F_K78_2_JX296719_Fn_Q5_Cedrela50	Q5	1	Glomus	
2_F_K78_4_JX297019_Fn_Q5_Cedrela50	Q5	67	Glomus	
2_F_K78_5_JX297021_Fn_Q5_Cedrela50	Q5	93	Acaulospora	
2_F_K79_5_JX297116_Fn_Q5_Cedrela51	Q5	130	Archaeospora	x
2_F_K79_9_JX296985_Fn_Cedrela51	Q5	66	Glomus	
2_F_K80_2_JX297110_Fp_Q5_Cedrela52	Q5	128	Archaeospora	x
2_F_K80_6_JX296955_Fp_Cedrela52	Q5	68	Glomus	
2_F_K81_1_JX297051_Fp_Q5_Cedrela53	Q5	88	Acaulospora	
2_F_K81_19_JX296960_Fp_Cedrela53	Q5	64	Glomus	
2_F_K81_2_JX297080_Fp_Q5_Cedrela53	Q5	114	Claroideoglomus	
2_F_K81_20_JX296707_Fp_Q5_Cedrela53	Q5	0	Glomus	
2_F_K81_22_JX296984_Fp_Cedrela53	Q5	66	Glomus	
2_F_K81_3_JX296749_Fp_Q5_Cedrela53	Q5	2	Glomus	
2_F_K81_6_JX297756_Fp_Q5_Cedrela53	Q5	7	Glomus	
2_F_K85_1_JX297023_F_Q5_Plant25	Q5	93	Acaulospora	
2_F_K85_4_JX296888_F_Q5_Plant25	Q5	44	Glomus	
2_F_K95_2_JX396731_F_Q5_Plant26	Q5	1	Glomus	
2_F_K95_4_JX296961_F_Q5_Plant26	Q5	65	Glomus	x
2_F_K96_2_JX297011_F_Q5	Q5	73	Glomus	x
2_F_K96_3_JX296789_F_Q5_Plant27	Q5	24	Glomus	x
2_F_K96_4_JX297057_F_Q5_Plant27	Q5	89	Acaulospora	x
3_14_106_2_MH052251_Cajanmua_Rynchospora	Clp	43	Glomus	
3_14_107_1_MH052311_Cajanuma_Chusquea	Clp	82	Acaulospora	
3_14_110_1_MH052292_Cajanuma_Eriocaulon	Clp	80	Acaulospora	
3_14_111_1_MH052308_Cajanuma_Hypericum1	Clp	82	Acaulospora	
3_14_112_1_MH052270_Cajanuma_Hypericum2	Clp	52	Glomus	
3_14_112_3_MH052269_Cajanuma_Hypericum2	Clp	49	Glomus	x
3_14_116_2_MH052230_Cajanuma_Melastoma	Clp	41	Glomus	
3_K1187_1_MH052295_Cajanuma_Paramo	Clp	84	Acaulospora	
3_K1189_5_MH052298_Cajanuma_Paramo	Clp	83	Acaulospora	
3_K1192_3_MH052227_Cajanuma_Paramo	Clp	41	Glomus	
3_K1195_2_MH052300_Cajanuma_Paramo	Clp	84	Acaulospora	
3_K1195_4_MH052328_Cajanuma_Paramo	Clp	87	Acaulospora	
3_K1196_2_MH052297_Cajanuma_Paramo	Clp	84	Acaulospora	
3_K1197_1_MH052320_Cajanuma_Paramo	Clp	84	Acaulospora	
3_K1199_1_MH052299_Cajanuma_Paramo	Clp	84	Acaulospora	
3_K1201_1_MH052228_Cajanuma_Paramo	Clp	41	Glomus	
3_K1202_6_MH052296_Cajanuma_Paramo	Clp	84	Acaulospora	
3_K290_1_MH052345_Cajanuma3	Cl	91	Acaulospora	
3_K373_1_MH052372_Cajanuma1	Cl	126	Archaeospora	
3_K374_1_MH052346_Cajanuma2	Cl	91	Acaulospora	
3_K374_7_MH052355_Cajanuma2	Cl	99	Acaulospora?	

3_K377_2_MH052247_Cajanuma20	Cl	43	Glomus	
3_K378_2_MH052280_Cajanuma24	Cl	80	Acaulospora	
3_K379_1_MH052266_Cajanuma5	Cl	43	Glomus	
3_K380_1_MH052360_Cajanuma6	Cl	104	Acaulospora	
3_K380_2_MH052253_Cajanuma6	Cl	43	Glomus	
3_K381_1_MH052317_Cajanuma7	Cl	82	Acaulospora	
3_K381_4_MH052324_Cajanuma7	Cl	86	Acaulospora?	
3_K384_6_MH052236_Cajanuma18	Cl	42	Glomus	
3_K387_1_MH052283_Cajanuma25	Cl	80	Acaulospora	
3_K388_1_MH052305_Cajanuma26	Cl	82	Acaulospora	
3_K388_2_MH052359_Cajanuma26	Cl	103	Acaulospora?	
3_K392_1_MH052302_Cajanuma30	Cl	81	Acaulospora	
3_K394_2_MH052262_Cajanuma32	Cl	43	Glomus	
3_K397_8_MH052303_Cajanuma40	Cl	81	Acaulospora	x
3_K398_2_MH052306_Cajanuma41	Cl	82	Acaulospora	
3_K403_1_MH052290_Cajanuma36	Cl	80	Acaulospora	
3_K406_6_MH052281_Cajanuma37	Cl	80	Acaulospora	
3_K407_5_MH052307_Cajanuma39	Cl	82	Acaulospora	
3_K412_2_MH052341_P63_Cajanuma44	Cl	87	Acaulospora	
3_K429_1_MH052267_Cajanuma45	Cl	48	Glomus	
3_K429_3_MH052335_Cajanuma45	Cl	87	Acaulospora	
3_K430_2_MH052362_Cajanuma45	Cl	104	Acaulospora	
3_K432_2_MH052223_Cajanuma40	Cl	41	Glomus	
3_K471_1_MH052235_Cajanuma16	Cl	42	Glomus	
3_K471_2_MH052334_Cajanuma16	Cl	87	Acaulospora	
3_K472_1_MH052363_Cajanuma19	Cl	104	Acaulospora	x
3_K472_2_MH052248_Cajanuma19	Cl	43	Glomus	
3_K474_2_MH052353_Cajanuma22	Cl	98	Acaulospora	x
3_K474_4_MH052325_Cajanuma22	Cl	87	Acaulospora	
3_K475_6_MH052279_Cajanuma23	Cl	80	Acaulospora	
3_K476_1_MH052220_Cajanuma13	Cl	41	Glomus	
3_K476_3_MH052330_Cajanuma13	Cl	87	Acaulospora	
3_K476_7_MH052237_Cajanuma	Cl	42	Glomus	
3_K477_2_MH052361_Cajanuma14	Cl	104	Acaulospora	
3_K477_4_MH052255_Cajanmua	Cl	43	Glomus	
3_K478_1_MH052327_Cajanuma15	Cl	87	Acaulospora	
3_K478_7_MH052238_Cajanuma15	Cl	42	Glomus	
3_K479_2_MH052232_Cajanuma18	Cl	42	Glomus	
3_K480_3_MH052364_Cajanuma22	Cl	110	Scutellospora	
3_K481_2_MH052347_Cajanuma22	Cl	91	Acaulospora	
3_K481_6_MH052329_Cajanuma22	Cl	87	Acaulospora	
3_K482_2_MH052240_Cajanuma23	Cl	42	Glomus	
3_K483_1_MH052249_Cajanuma	Cl	43	Glomus	
3_K483_4_MH052337_Cajanuma26	Cl	87	Acaulospora	
3_K485_1_MH052225_Cajanuma35	Cl	41	Glomus	
3_K486_1_MH052219_Cajanuma12	Cl	41	Glomus	

3_K486_2_MH052354_Cajanuma12	Cl	98	Acaulospora	
3_K487_1_MH052288_Cajanuma36	Cl	80	Acaulospora	
3_K488_1_MH052221_Cajanuma38	Cl	41	Glomus	
3_K489_1_MH052282_Cajanuma40	Cl	80	Acaulospora	
3_K489_2_MH052294_Cajanuma40	Cl	84	Acaulospora	
3_K489_4_MH052375_Cajanuma	Cl	131	Archaeospora	
3_K489_7_MH052256_Cajanuma	Cl	43	Glomus	
3_K490_1_MH052254_Cajanuma41	Cl	43	Glomus	
3_K491_1_MH052272_Cajanuma44	Cl	60	Glomus	
3_K492_1_MH052268_Cajanuma45	Cl	48	Glomus	
3_K492_7_MH052352_Cajanuma45	Cl	93	Acaulospora	
3_K492_8_MH052216_Cajanuma	Cl	25	Glomus	
3_K509_1_MH052250_Cajanuma19	Cl	43	Glomus	
3_K511_1_MH052226_Cajanuma34	Cl	41	Glomus	
3_K634_1_MH052215_Cajanuma8	Cl	17	Glomus	
3_K634_3_MH052289_Cajanuma8	Cl	80	Acaulospora	
3_K635_3_MH052257_Cajanuma17	Cl	43	Glomus	
3_K811_2_MH052357_Cajanuma03_14_3200m	Clp	103	Acaulospora?	
3_K813_1_MH052322_Cajanuma03_14_3200m	Clp	86	Acaulospora?	
3_K813_5_MH052229_Cajanuma03_14_3200m	Clp	41	Glomus	
3_K908_1_MH052258_Cajanuma_Coreplot_unten	Cc	43	Glomus	x
3_K909_1_MH052373_Cajanuma_Coreplot_unten	Cc	126	Archaeospora	
3_K910_1_MH052239_Cajanuma_Coreplot_unten	Cc	42	Glomus	
3_K914_2_MH052376_Cajanuma_Coreplot_unten	Cc	133	Ambispora	x
3_K915_4_MH052351_Cajanuma_Coreplot_unten	Cc	91	Acaulospora	
3_K916_14_MH052371_Cajanuma_Coreplot_unten	Cc	126	Archaeospora	
3_K917_1_MH052318_Cajanuma_Coreplot_unten	Cc	82	Acaulospora	x
3_K917_4_MH052336_Cajanuma_Coreplot_unten	Cc	87	Acaulospora	
3_K920_14_MH052319_Cajanuma_Coreplot_unten	Cc	82	Acaulospora	
3_K923_6_MH052241_Cajanuma_Coreplot_unten	Cc	42	Glomus	
3_K925_1_MH052340_Cajanuma_Coreplot_unten	Cc	87	Acaulospora	
3_K926_11_MH052339_Cajanuma_Coreplot_unten	Cc	87	Acaulospora	
3_K927_9_MH052332_Cajanuma_Coreplot_unten	Cc	87	Acaulospora	
3_K929_1_MH052261_Cajanuma_Coreplot_unten	Cc	43	Glomus	
3_K929_3_MH052358_Cajanuma_Coreplot_unten	Cc	103	Acaulospora?	
3_K930_2_MH052374_Cajanuma_Coreplot_unten	Cc	131	Archaeospora	x
3_K931_2_MH052309_Cajanuma_Coreplot_unten	Cc	82	Acaulospora	
3_K932_6_MH052242_Cajanuma_Coreplot_unten	Cc	42	Glomus	
3_K933_4_MH052333_Cajanuma_Coreplot_unten	Cc	87	Acaulospora	
3_K933_8_MH052367_Cajanuma_Coreplot_unten	Cc	115	Claroideoglomus	
3_K934_1_MH052293_Cajanuma_Coreplot_unten	Cc	80	Acaulospora	
3_K934_13_MH052349_Cajanuma_Coreplot_unten	Cc	91	Acaulospora	
3_K936_4_MH052207_Cajanuma_Coreplot_unten	Cc	1	Glomus	
3_K936_6_MH052343_Cajanuma_Coreplot_unten	Cc	90	Acaulospora	
3_K937_3_MH052342_Cajanuma_Coreplot_unten	Cc	90	Acaulospora	x
3_K937_5_MH052365_Cajanuma_Coreplot_unten	Cc	110	Scutellospora	

3_K937_6_MH052326_Cajanuma_Coreplot_unten	Cc	87	Acaulospora	
3_K938_10_MH052366_Cajanuma_Coreplot_unten	Cc	115	Claroideoglomus	
3_K938_6_MH052208_Cajanuma_Coreplot_unten	Cc	1	Glomus	
3_K939_1_MH052224_Cajanuma_Coreplot_unten	Cc	41	Glomus	
3_K939_2_MH052217_Cajanuma_Coreplot_unten	Cc	38	Glomus	x
3_K939_3_MH052243_Cajanuma_Coreplot_unten	Cc	42	Glomus	
3_K940_2_MH052350_Cajanuma_Coreplot_unten	Cc	91	Acaulospora	
3_K942_10_MH052369_Cajanuma_Coreplot_unten	Cc	115	Claroideoglomus	
3_K943_5_MH052338_Cajanuma_Coreplot_unten	Cc	87	Acaulospora	
3_K944_1_MH052370_Cajanuma_Coreplot_unten	Cc	122	Archaeospora	
3_K944_11_MH052331_Cajanuma_Coreplot_unten	Cc	87	Acaulospora	
3_K945_10_MH052315_Cajanuma_Coreplot_unten	Cc	82	Acaulospora	
3_K945_12_MH052348_Cajanuma_Coreplot_unten	Cc	91	Acaulospora	x
3_K946_9_MH052245_Cajanuma_Coreplot_unten	Cc	42	Glomus	
3_K947_10_MH052271_Cajanuma_Coreplot_unten	Cc	55	Glomus	
3_K947_16_MH052356_Cajanuma_Coreplot_unten	Cc	102	Acaulospora	x
3_K947_9_MH052260_Cajanuma_Coreplot_unten	Cc	43	Glomus	
3_K949_9_MH052244_Cajanuma_Coreplot_unten	Cc	42	Glomus	
3_K950_10_MH052209_Cajanuma_Coreplot_unten	Cc	1	Glomus	
3_K950_15_MH052314_Cajanuma_Coreplot_unten	Cc	82	Acaulospora	
3_K950_16_MH052368_Cajanuma_Coreplot_unten	Cc	115	Claroideoglomus	
3_K950_9_MH052218_Cajanuma_Coreplot_unten	Cc	39	Glomus	x
3_K951_16_MH052344_Cajanuma_Coreplot_unten	Cc	90	Acaulospora	
3_K951_9_MH052233_Cajanuma_Coreplot_unten	Cc	42	Glomus	
3_K953_10_MH052212_Cajanuma_Coreplot_unten	Cc	6	Glomus	x
3_K953_4_MH052246_Cajanuma_Coreplot_unten	Cc	42	Glomus	
3_K954_2_MH052273_Cajanuma_Mirador	Cm	80	Acaulospora	
3_K955_2_MH052259_Cajanuma_Mirador	Cm	43	Glomus	
3_K956_1_MH052312_Cajanuma_Mirador	Cm	82	Acaulospora	
3_K956_3_MH052323_Cajanuma_Mirador	Cm	86	Acaulospora?	x
3_K956_8_MH052231_Cajanuma_Mirador	Cm	41	Glomus	
3_K958_3_MH052276_Cajanuma_Mirador	Cm	80	Acaulospora	
3_K959_4_MH052310_Cajanuma_Mirador	Cm	82	Acaulospora	
3_K961_5_MH052284_Cajanuma_Mirador	Cm	80	Acaulospora	
3_K962_1_MH052274_Cajanuma_Mirador	Cm	80	Acaulospora	
3_K963_1_MH052316_Cajanuma_Mirador	Cm	82	Acaulospora	
3_K963_2_MH052277_Cajanuma_Mirador	Cm	80	Acaulospora	
3_K964_3_MH052278_Cajanuma_Mirador	Cm	80	Acaulospora	
3_K965_1_MH052304_Cajanuma_Mirador	Cm	81	Acaulospora	
3_K967_15_MH052285_Cajanuma_Mirador	Cm	80	Acaulospora	
3_K968_12_MH052301_Cajanuma_Mirador	Cm	80	Acaulospora	
3_K968_5_MH052264_Cajanuma_Mirador	Cm	43	Glomus	
3_K969_3_MH052211_Cajanuma_Coreplot_unten	Cc	1	Glomus	
3_K970_14_MH052210_Cajanuma_Coreplot_unten	Cc	1	Glomus	
3_K970_4_MH052275_Cajanuma_Coreplot_unten	Cc	80	Acaulospora	
3_K974_8_MH052234_Cajanuma_Mirador	Cm	42	Glomus	x

3_K975_7_MH052286_Cajanuma_Mirador	Cm	80	Acaulospora	
3_K977_16_MH052214_Cajanuma_Mirador	Cm	6	Glomus	
3_K978_1_MH052265_Cajanuma_Mirador	Cm	43	Glomus	
3_K978_3_MH052222_Cajanuma_Mirador	Cm	41	Glomus	
3_K978_8_MH052213_Cajanuma_Mirador	Cm	6	Glomus	
3_K981_1_MH052263_Cajanuma_Mirador	Cm	43	Glomus	
3_K981_4_MH052287_Cajanuma_Mirador	Cm	80	Acaulospora	
3_K983_3_MH052321_Cajanuma_Mirador	Cm	85	Acaulospora	
3_K985_4_MH052252_Cajanuma_Mirador	Cm	43	Glomus	
3_K987_4_MH052291_Cajanuma_Mirador	Cm	80	Acaulospora	
3_K989_2_MH052313_Cajanuma_Mirador	Cm	82	Acaulospora	
33_K1155_2_MH052506_Nero_3300m	N	87	Acaulospora	
33_K1156_2_MH052507_Nero_3300m	N	87	Acaulospora	
33_K1159_5_MH052504_Nero_3300m	N	87	Acaulospora	
33_K1160_1_MH052499_Nero_3300m	N	36	Glomus	
33_K1161_1_MH052505_Nero_3300m	N	87	Acaulospora	
33_K1162_1_MH052501_Nero_3300m	N	84	Acaulospora	
33_K1162_2_MH052500_Nero_3300m	N	41	Glomus	x
33_K1162_3_MH052509_Nero_3300m	N	87	Acaulospora	
33_K1163_1_MH052508_Nero_3300m	N	87	Acaulospora	
33_K1163_2_MH052502_Nero_3300m	N	84	Acaulospora	
33_K1164_2_MH052503_Nero_3300m	N	82	Acaulospora	
35_K1026_1_MH052543_Tutupali	Tu	87	Acaulospora	
35_K1028_2_MH052558_Tutupali	Tu	26	Glomus	
35_K1029_1_MH052559_Tutupali	Tu	26	Glomus	
35_K1029_2_MH052539_Tutupali	Tu	87	Acaulospora	
35_K1030_2_MH052568_Tutupali	Tu	87	Acaulospora	
35_K1031_1_MH052563_Tutupali	Tu	84	Acaulospora	
35_K1031_2_MH052547_Tutupali	Tu	87	Acaulospora	
35_K1032_1_MH052567_Tutupali	Tu	84	Acaulospora	
35_K1032_3_MH052566_Tutupali	Tu	82	Acaulospora	
35_K1033_1_MH052561_Tutupali	Tu	41	Glomus	
35_K1033_2_MH052565_Tutupali	Tu	82	Acaulospora	
35_K1033_7_MH052541_Tutupali	Tu	87	Acaulospora	
35_K1053_2_MH052553_Tutupali	Tu	102	Acaulospora	
35_K1054_1_MH052545_Tutupali	Tu	87	Acaulospora	
35_K1056_1_MH052544_Tutupali	Tu	87	Acaulospora	
35_K1059_1_MH052552_Tutupali	Tu	92	Acaulospora	
35_K1059_2_MH052548_Tutupali	Tu	91	Acaulospora	
35_K1060_2_MH052551_Tutupali	Tu	91	Acaulospora	
35_K1060_5_MH052560_Tutupali	Tu	40	Glomus	x
35_K1061_1_MH052549_Tutupali	Tu	91	Acaulospora	
35_K1061_8_MH052546_Tutupali	Tu	87	Acaulospora	
35_K1062_3_MH052549_Tutupali	Tu	91	Acaulospora	
35_K1064_1_MH052562_Tutupali	Tu	84	Acaulospora	
35_K1064_3_MH052554_Tutupali	Tu	115	Claroideoglomus	

35_K1166_2_MH052556_Tutupali	Tu	0	Glomus	
35_K1167_1_MH052540_Tutupali	Tu	87	Acaulospora	
35_K1167_2_MH052564_Tutupali	Tu	84	Acaulospora	
35_K1168_9_MH052557_Tutupali	Tu	23	Glomus	
35_K1169_2_MH052542_Tutupali	Tu	87	Acaulospora	
35_K1259_1_MH052555_Tutupali	Tu	125	Archaeospora	x
37_K1072_2_MH052528_Soldados	So	91	Acaulospora	
37_K1072_3_MH052516_Soldados	So	41	Glomus	
37_K1073_4_MH052512_Soldados	So	23	Glomus	
37_K1075_2_MH052517_Soldados	So	41	Glomus	
37_K1075_5_MH052525_Soldados	So	55	Glomus	
37_K1075_7_MH052510_Soldados	So	23	Glomus	
37_K1076_2_MH052521_Soldados	So	55	Glomus	
37_K1077_1_MH052511_Soldados	So	23	Glomus	
37_K1078_1_MH052533_Soldados	So	116	Claroideoglomus	x
37_K1078_2_MH052520_Soldados	So	48	Glomus	
37_K1078_4_MH052538_Soldados	So	23	Glomus	
37_K1079_1_MH052514_Soldados	So	39	Glomus	
37_K1079_2_MH052526_Soldados	So	55	Glomus	x
37_K1079_7_MH052534_Soldados	So	117	Claroideoglomus	
37_K1080_2_MH052536_Soldados	So	1	Glomus	
37_K1080_8_MH052531_Soldados	So	115	Claroideoglomus	
37_K1081_1_MH052523_Soldados	So	55	Glomus	
37_K1081_2_MH052515_Soldados	So	39	Glomus	
37_K1081_4_MH052529_Soldados	So	106	Diversispora	x
37_K1082_2_MH052518_Soldados	So	48	Glomus	x
37_K1083_2_MH052532_Soldados	So	115	Claroideoglomus	x
37_K1083_3_MH052535_Soldados	So	1	Glomus	
37_K1083_4_MH052519_Soldados	So	48	Glomus	
37_K1084_1_MH052522_Soldados	So	55	Glomus	
37_K1085_1_MH052530_Soldados	So	114	Claroideoglomus	
37_K1085_4_MH052537_Soldados	So	1	Glomus	
37_K1086_3_MH052513_Soldados	So	25	Glomus	
37_K1086_6_MH052524_Soldados	So	55	Glomus	
37_K1247_7_MH052527_Soldados	So	56	Glomus	
4_K1002_2_MH052478_Cajas_Polylepis	Cpo	82	Acaulospora	
4_K1003_1_MH052470_Cajas_Polylepis	Cpo	41	Glomus	
4_K1003_3_MH052490_Cajas_Polylepis	Cpo	103	Acaulospora?	
4_K1004_1_MH052477_Cajas_Polylepis	Cpo	82	Acaulospora	
4_K1005_1_MH052468_Cajas_Polylepis	Cpo	41	Glomus	
4_K1005_2_MH052496_Cajas_Polylepis	Cpo	115	Claroideoglomus	
4_K1034_2_MH052495_Cajas_Polylepis	Cpo	115	Claroideoglomus	
4_K1035_1_MH052469_Cajas_Polylepis	Cpo	41	Glomus	
4_K1035_3_MH052491_Cajas_Polylepis	Cpo	103	Acaulospora?	
4_K1036_1_MH052481_Cajas_Polylepis	Cpo	82	Acaulospora	
4_K1038_3_MH052484_Cajas_Polylepis	Cpo	87	Acaulospora	

4_K1038_4_MH052492_Cajas_Polylepis	Cpo	103	Acaulospora?	x
4_K1038_5_MH052471_Cajas_Polylepis	Cpo	41	Glomus	
4_K1039_1_MH052483_Cajas_Polylepis	Cpo	87	Acaulospora	
4_K1040_1_MH052479_Cajas_Polylepis	Cpo	82	Acaulospora	
4_K1041_1_MH052498_Cajas_Polylepis	Cpo	115	Claroideoglomus	
4_K1042_1_MH052485_Cajas_Polylepis	Cpo	87	Acaulospora	
4_K1044_1_MH052473_Cajas_Polylepis	Cpo	43	Glomus	
4_K1044_3_MH052487_Cajas_Polylepis	Cpo	87	Acaulospora	
4_K1044_6_MH052489_Cajas_Polylepis	Cpo	103	Acaulospora?	
4_K1045_1_MH052488_Cajas_Polylepis	Cpo	87	Acaulospora	x
4_K1046_1_MH052494_Cajas_Polylepis	Cpo	115	Claroideoglomus	
4_K1046_6_MH052475_Cajas_Polylepis	Cpo	43	Glomus	
4_K1048_3_MH052493_Cajas_Polylepis	Cpo	115	Claroideoglomus	
4_K1087_4_MH052414_Cajas_Paramo	Cpa	82	Acaulospora	
4_K1088_2_MH052447_Cajas_Paramo	Cpa	92	Acaulospora	
4_K1089_1_MH052440_Cajas_Paramo	Cpa	92	Acaulospora	
4_K1091_2_MH052445_Cajas_Paramo	Cpa	92	Acaulospora	x
4_K1092_1_MH052448_Cajas_Paramo	Cpa	92	Acaulospora	
4_K1093_2_MH052418_Cajas_Paramo	Cpa	82	Acaulospora	
4_K1093_3_MH052398_Cajas_Paramo	Cpa	41	Glomus	
4_K1094_2_MH052431_Cajas_Paramo	Cpa	82	Acaulospora	
4_K1095_6_MH052441_Cajas_Paramo	Cpa	92	Acaulospora	
4_K1096_1_MH052450_Cajas_Paramo	Cpa	92	Acaulospora	
4_K1097_3_MH052377_Cajas_Paramo	Cpa	6	Glomus	
4_K1097_4_MH052388_Cajas_Paramo	Cpa	25	Glomus	
4_K1098_1_MH052436_Cajas_Paramo	Cpa	86	Acaulospora?	
4_K1099_2_MH052434_Cajas_Paramo	Cpa	86	Acaulospora?	
4_K1099_6_MH052390_Cajas_Paramo	Cpa	25	Glomus	
4_K1100_3_MH052411_Cajas_Paramo	Cpa	84	Acaulospora	x
4_K1101_1_MH052395_Cajas_Paramo	Cpa	40	Glomus	
4_K1101_2_MH052426_Cajas_Paramo	Cpa	82	Acaulospora	
4_K1101_3_MH052378_Cajas_Paramo	Cpa	23	Glomus	x
4_K1102_1_MH052389_Cajas_Paramo	Cpa	25	Glomus	
4_K1102_6_MH052379_Cajas_Paramo	Cpa	23	Glomus	
4_K1103_1_MH052394_Cajas_Paramo	Cpa	25	Glomus	x
4_K1103_2_MH052405_Cajas_Paramo	Cpa	43	Glomus	
4_K1104_1_MH052380_Cajas_Paramo	Cpa	23	Glomus	
4_K1104_6_MH052435_Cajas_Paramo	Cpa	86	Acaulospora?	
4_K1105_1_MH052386_Cajas_Paramo	Cpa	25	Glomus	
4_K1106_1_MH052404_Cajas_Paramo	Cpa	43	Glomus	
4_K1106_3_MH052393_Cajas_Paramo	Cpa	25	Glomus	
4_K1107_1_MH052438_Cajas_Paramo	Cpa	86	Acaulospora?	
4_K1107_2_MH052403_Cajas_Paramo	Cpa	43	Glomus	
4_K1108_5_MH052449_Cajas_Paramo	Cpa	92	Acaulospora	
4_K1109_3_MH052453_Cajas_Paramo	Cpa	92	Acaulospora	
4_K1110_8_MH052442_Cajas_Paramo	Cpa	92	Acaulospora	

4_K1111_4_MH052443_Cajas_Paramo	Cpa	92	Acaulospora	
4_K1112_1_MH052452_Cajas_Paramo	Cpa	92	Acaulospora	
4_K1113_1_MH052444_Cajas_Paramo	Cpa	92	Acaulospora	
4_K1114_3_MH052397_Cajas_Paramo	Cpa	41	Glomus	
4_K1114_4_MH052424_Cajas_Paramo	Cpa	82	Acaulospora	
4_K1115_1_MH052410_Cajas_Paramo	Cpa	84	Acaulospora	
4_K1115_6_MH052381_Cajas_Paramo	Cpa	23	Glomus	
4_K1116_1_MH052383_Cajas_Paramo	Cpa	23	Glomus	
4_K1116_2_MH052407_Cajas_Paramo	Cpa	84	Acaulospora	
4_K1117_1_MH052417_Cajas_Paramo	Cpa	82	Acaulospora	
4_K1117_2_MH052413_Cajas_Paramo	Cpa	84	Acaulospora	
4_K1118_1_MH052392_Cajas_Paramo	Cpa	25	Glomus	
4_K1119_1_MH052427_Cajas_Paramo	Cpa	82	Acaulospora	
4_K1120_1_MH052412_Cajas_Paramo	Cpa	84	Acaulospora	
4_K1121_5_MH052451_Cajas_Paramo	Cpa	92	Acaulospora	
4_K1122_1_MH052446_Cajas_Paramo	Cpa	92	Acaulospora	
4_K1125_1_MH052387_Cajas_Paramo	Cpa	25	Glomus	
4_K1129_5_MH052430_Cajas_Paramo	Cpa	82	Acaulospora	
4_K1130_3_MH052400_Cajas_Paramo	Cpa	41	Glomus	
4_K1132_7_MH052429_Cajas_Paramo	Cpa	82	Acaulospora	
4_K1133_4_MH052425_Cajas_Paramo	Cpa	82	Acaulospora	
4_K1134_1_MH052419_Cajas_Paramo	Cpa	82	Acaulospora	
4_K1135_1_MH052382_Cajas_Paramo	Cpa	23	Glomus	
4_K1135_7_MH052408_Cajas_Paramo	Cpa	81	Acaulospora	
4_K1136_1_MH052422_Cajas_Paramo	Cpa	82	Acaulospora	
4_K1136_2_MH052401_Cajas_Paramo	Cpa	43	Glomus	
4_K1137_3_MH052409_Cajas_Paramo	Cpa	82	Acaulospora	
4_K1137_7_MH052406_Cajas_Paramo	Cpa	84	Acaulospora	
4_K1138_3_MH052428_Cajas_Paramo	Cpa	82	Acaulospora	
4_K1138_6_MH052399_Cajas_Paramo	Cpa	41	Glomus	
4_K1141_1_MH052385_Cajas_Paramo	Cpa	25	Glomus	
4_K1141_2_MH052437_Cajas_Paramo	Cpa	86	Acaulospora?	
4_K1141_6_MH052433_Cajas_Paramo	Cpa	85	Acaulospora	x
4_K1142_1_MH052420_Cajas_Paramo	Cpa	82	Acaulospora	
4_K1142_2_MH052402_Cajas_Paramo	Cpa	43	Glomus	
4_K1142_3_MH052396_Cajas_Paramo	Cpa	40	Glomus	
4_K1143_1_MH052421_Cajas_Paramo	Cpa	82	Acaulospora	
4_K1144_1_MH052439_Cajas_Paramo	Cpa	86	Acaulospora?	
4_K1144_2_MH052384_Cajas_Paramo	Cpa	25	Glomus	
4_K1144_5_MH052432_Cajas_Paramo	Cpa	82	Acaulospora	
4_K1148_2_MH052415_Cajas_Paramo	Cpa	82	Acaulospora	
4_K1149_2_MH052416_Cajas_Paramo	Cpa	82	Acaulospora	
4_K1151_1_MH052391_Cajas_Paramo	Cpa	25	Glomus	
4_K1152_1_MH052423_Cajas_Paramo	Cpa	82	Acaulospora	
4_K1229_3_MH052482_Cajas_Polylepis	Cpo	84	Acaulospora	
4_K1230_1_MH052472_Cajas_Polylepis	Cpo	41	Glomus	

4_K1231_2_MH052480_Cajas_Polylepis	Cpo	82	Acaulospora	
4_K1234_1_MH052486_Cajas_Polylepis	Cpo	87	Acaulospora	
4_K1235_2_MH052474_Cajas_Polylepis	Cpo	43	Glomus	
4_K1237_6_MH052497_Cajas_Polylepis	Cpo	5	Glomus	
4_K1238_1_MH052476_Cajas_Polylepis	Cpo	82	Acaulospora	
4_K841_11_MH052458_Cajas_Polylepis1	Cpo	11	Glomus	
4_K841_3_MH052461_Cajas_Polylepis1	Cpo	36	Glomus	x
4_K842_1_MH052465_Cajas_Polylepis2	Cpo	36	Glomus	
4_K843_4_MH052462_Cajas_Polylepis3	Cpo	36	Glomus	
4_K844_4_MH052460_Cajas_Polylepis4	Cpo	36	Glomus	
4_K845_3_MH052466_Cajas_Polylepis5	Cpo	36	Glomus	
4_K846_3_MH052463_Cajas_Polylepis6	Cpo	36	Glomus	
4_K847_10_MH052467_Cajas_Polylepis7	Cpo	36	Glomus	
4_K848_1_MH052464_Cajas_Polylepis8	Cpo	36	Glomus	
4_K852_2_MH052459_Cajas_Polylepis12	Cpo	36	Glomus	
4_K854_1_MH052454_Cajas_Calamagrostis	Cpa	36	Glomus	
4_K855_3_MH052455_Cajas_Calamagrostis	Cpa	36	Glomus	
4_K856_2_MH052456_Cajas_Calamagrostis7	Cpa	36	Glomus	
4_K856_6_MH052457_Calamagrostis7_Cajas	Cpa	1	Glomus	
AB555661_Sciaphila_japonica		14	Glomus	
AB555673_Sciaphila_japonica		28	Glomus	
AB556919_Sciaphila_tosaensis		57	Glomus	
AB556928_Sciaphila_tosaensis		58	Glomus	
AB594911_Osumunda_japonica_Japan		66	Glomus	
AB594922_Osumunda_japonica_Japan		23	Glomus	
AF213462_Rhizophagus_proliferus		8	Rhizophagus	
AJ133706_Sclerocystis_sinuosa		31	Glomus	
AJ245637_Funneliformis_geosporum		77	Funneliformis	
AJ276075_Claroideoglomus_claroideum		115	Claroideoglomus	
AJ276083_Claroideoglomus_lamellosum		115	Claroideoglomus	
AJ276085_Funneliformis_fragilistratum		77	Funneliformis	
AJ276086_Funneliformis_coronatum		77	Funneliformis	
AJ276092_Scutellospora_aurigloba		111	Scutellospora	
AJ301858_Funneliformis_verruculosum		77	Funneliformis	
AJ301859_Rhizophagus_irregularis_VT114		1	Rhizophagus	
AJ301862_Paraglomus_brasilianum		121	Paraglomus	
AJ306440_Acaulospora_W3424		83	Acaulospora	
AJ699068_Glomus_Marchantia_foliacea_VTX74		23	Glomus	
AJ699069_Gl_Marchantia_foliacea		57	Glomus	
AJ699070_Glomus_Marchantia_NZ_VTX73		38	Glomus	
AJ852530_Glomus_sp_MUCL_43206		1	Glomus	
AJ852603_Gigaspora_margarita		109	Gigaspora	
AM114274_Archaeospora_trappei		134	Archaeosora	
AY635831_Rhizophagus_intraradices_VT114		1	Rhizophagus	
AY635833_Funneliformis_mosseae		77	Funneliformis	
AY903734_Glomus_Botrychium		60	Glomus	

DQ085211_Glomus_JP6		53	Glomus	
DQ085256_Glomus_JP4		78	Funneliformis	
DQ396707_Glomus_PF11		57	Glomus	
DQ396709_Uncultured_Glomus_PF14_VT83		34	Glomus	
DQ396749_Glomus_Podocarpus_VTX191		42	Glomus	
EF136887_Acaulospora_sp		91	Acaulospora	
EU417581_Glomus_Afrothismia		30	Glomus	
EU417622_Glomus_Afrothismia		5	Glomus	
EU417640_Glomus_Sciaphila_VT166		57	Glomus	
FJ009612_Rhizophagus_irregularis_clone_08		1	Rhizophagus	
FJ009618_Rhizophagus_irregularis_clone_14		1	Rhizophagus	
FJ009670_Acaulospora_mellea		95	Acaulospora	
FJ009672_Scutellospora_calospora		112	Scutellospora	
FJ831530_Glomus_Olea_NF02		7	Glomus	
FJ831546_Glomus_535_PFB_NF06		66	Glomus	
FN825899_Acaulospora_brasiliensis_VTX230		84	Acaulospora	
FR686954_Diversispora_spurca		105	Diversispora	
FR750213_Acaulospora_sp_Att1186_5		91	Acaulospora	
GQ140621_Glomus_Perilla_China		68	Glomus	
GQ140623_Archaeospora_VTX4		129	Archaeospora	
HE610427_Acaulospora_lacunosa		95	Acaulospora	
HE613472_Uncultured_Glomus_partial_VT174		74	Glomus	
HE615060_Glomus_VTX312		6	Glomus	
HF968771_Gigaspora_margarita		109	Gigaspora	
HG004504_Glomus_Sonchus_VTX113		39	Glomus	
HQ202289_Scutellospora_crenulata		111	Scutellospora	
HQ258992_Uncultured_Archaeospora		125	Archaeospora	
JF414186_Symphyogyna_Australia		91	Acaulospora	
JF414188_Ambispora_VTX283		133	Ambispora	
JF414190_Symphyogyna_NewZealand		41	Glomus	
JF414191_Glomeromycota_Symphyogyna		43	Glomus	
JN252439_Uncultured_Acaulospora_VTX26		89	Acaulospora	
JN644467_apple_tree_Halle		68	Glomus	
JQ811204_Glomus_sp_Preissia_UK_VT113		1	Glomus	
KC708343_Phaeoceros_carolinianus		55	Glomus	
KC708365_Claroideoglomus_Megaceros_NZ_VTX193		115	Claroideoglomus	
KC708371_Arch_Folioceros_China_VTX5		123	Archaeospora	
KC708376_Phaeoceros_laevis		57	Glomus	
KF386272_Acaulospora_VTX30		87	Acaulospora	
KF386291_Uncultured_Sclerocystis		24	Glomus	
KY174019_Uncultured_Glomus		40	Glomus	
L20824_Rhizophagus_vesiculiferus_VT115		1	Rhizophagus	
NG017179_Paraglomus_occultum		120	Paraglomus	
X58725_Rhizophagus_intraradices_VT114		1	Rhizophagus	
Y17633_Acaulospora_laevis		87	Acaulospora	
Y17639_Claroideoglomus_etunicatum		115	Claroideoglomus	

Y17640_Rhizophagus_fasciculatus_VT113		1	Rhizophagus	
Y17653_Funneliformis_caledonium		77	Funneliformis	
Z14010_Gigaspora_gigantea		109	Gigaspora	
