# Supplementary material for: Species composition of arbuscular mycorrhizal communities changes with elevation in the Andes of South Ecuador
Source: PLoS One. 2019 Aug 16;14(8):e0221091. doi: 10.1371/journal.pone.0221091 (PMC6697372; doi:10.1371/journal.pone.0221091)
Supplement: S3 Table — (PDF) [file pone.0221091.s006.pdf]

S3 Table	
Object	OTU No
2_F_K241_3_KX107977_Fn_Q5_Cedrela	0
2_F_K399_1_KX107972_F_T2_2550m_Graffenrieda	0
2_F_K679_7_KX107968_T2_950	1
2_F_K680_1_KX107969_T2_950_OTU31	1
3_K492_8_Cajanuma	1
35_K1028_2_Tutupali	1
35_K1029_1_Tutupali	1
37_K1086_3_Soldados	1
4_K1097_4_Cajas_Paramo	1
4_K1099_6_Cajas_Paramo	1
4_K1102_1_Cajas_Paramo	1
4_K1103_1_Cajas_Paramo	1
4_K1105_1_Cajas_Paramo	1
4_K1106_3_Cajas_Paramo	1
4_K1118_1_Cajas_Paramo	1
4_K1125_1_Cajas_Paramo	1
4_K1141_1_Cajas_Paramo	1
4_K1144_2_Cajas_Paramo	1
4_K1151_1_Cajas_Paramo	1
AB555673_Sciaphila_japonica	1
DQ396709_Uncultured_Glomus_PF14_VT83	1
1_K493_2_KX108455_Bombuscarol_1s	2
1_K494_1_KX108346_Bombuscarol_2s	2
1_K494_4_KX108392_Bombuscarol_2s	2
1_K495_1_KX108444_Bombuscarol_3s	2
1_K496_5_KX108260_Bombuscarol_5g	2
1_K497_1_KX108446_Bombuscarol_6s	2
1_K497_3_KX108354_Bombuscarol_6s	2
1_K498_3_KX108366_Bombuscarol_7s	2
1_K499_1_KX108198_Bombuscarol_8s	2
1_K499_6_KX108432_Bombuscarol_8s	2
1_K500_1_KX108360_Bombuscarol_9s	2
1_K502_1_KX108324_Bombuscarol_11s	2
1_K502_2_KX108419_Bombuscarol_11s	2
1_K503_4_KX108415_Bombuscarol_12	2
1_K503_6_KX108353_Bombuscarol_12	2
1_K503_8_KX108485_Bombuscarol_12	2
1_K506_2_KX108254_Bombuscarol_15	2
1_K506_3_KX108420_Bombuscarol_15	2
1_K508_1_KX108363_Bombuscarol_17	2
1_K508_3_KX108259_Bombuscarol_17	2
1_K512_1_KX108351_Bombuscarol_4	2
1_K512_2_KX108488_Bombuscarol_4	2
1_K513_2_KX108393_Bombuscarol_18	2

1_K514_4_KX108397_Bombuscarol_19	2
1_K517_1_KX108272_Bombuscarol_22	2
1_K517_2_KX108437_Bombuscarol_22	2
1_K518_1_KX108403_Bombuscarol_23b	2
1_K519_2_KX108355_Bombuscarol_24	2
1_K520_3_KX108398_Bombuscarol_26	2
1_K521_1_KX108475_Bombuscarol_27	2
1_K521_3_KX108352_Bombuscarol_27	2
1_K522_4_KX108402_Bombuscarol_57	2
1_K523_2_KX108290_Bombuscarol_29	2
1_K523_5_KX108395_Bombuscarol_29	2
1_K524_2_KX108476_Bombuscarol_30	2
1_K527_3_KX108373_Bombuscarol_33	2
1_K527_8_KX108282_Bombuscarol_33	2
1_K528_2_KX108321_Bombuscarol_33	2
1_K529_1_KX108487_Bombuscarol_34_OTU28	2
1_K529_4_KX108383_Bombuscarol_34	2
1_K531_1_KX108362_Bombuscarol_36	2
1_K532_2_KX108394_Bombuscarol_37_OTU21	2
1_K532_7_KX108372_Bombuscarol_37	2
1_K533_16_KX108421_Bombuscarol_38	2
1_K534_12_KX108357_Bombuscarol_39	2
1_K535_10_KX108449_Bombuscarol_40	2
1_K536_1_KX108350_Bombuscarol_41g_OTU20	2
1_K536_7_KX108463_Bombuscarol_41g	2
1_K538_1_KX108450_Bombuscarol_43	2
1_K538_5_KX108308_Bombuscarol_43_OTU17	2
1_K540_1_KX108480_Bombuscarol_OTU22	2
1_K541_4_KX108331_Bombuscarol_46	2
1_K541_5_KX108405_Bombuscarol_46	2
1_K542_14_KX108414_Bombuscarol_47	2
1_K542_5_KX108484_Bombuscarol_47	2
1_K543_10_KX108486_Bombuscarol_48	2
1_K543_9_KX108369_Bombuscarol_48	2
1_K546_1_KX108311_Bombuscarol_51_OTU18	2
1_K546_7_KX108364_Bombuscarol_51	2
1_K547_8_KX108447_Bombuscarol_52	2
1_K548_1_KX108341_Bombuscarol_53g	2
1_K548_2_KX108477_Bombuscarol_53g	2
1_K548_5_KX108296_Bombuscarol_53g_OTU14	2
1_K549_2_KX108266_Bombuscarol_54g	2
1_K549_4_KX108375_Bombuscarol_54g	2
1_K550_1_KX108441_Bombuscarol_55	2
1_K550_2_KX108380_Bombuscarol_55	2
1_K551_3_KX108433_Bombuscarol_56	2
1_K551_6_KX108367_Bombuscarol_56	2

1_K552_1_KX108448_BombuscaroI_57_OTU21	2
1_K553_2_KX108265_BombuscaroI_58g	2
1_K554_1_KX108281_BombuscaroI_59g	2
1_K554_2_KX108422_BombuscaroI_59g	2
1_K555_4_KX108323_BombuscaroI_60	2
1_K556_2_KX108479_BombuscaroI_61	2
1_K556_3_KX108348_BombuscaroI_61	2
1_K557_2_KX108325_BombuscaroI_62_OTU20	2
1_K557_3_KX108396_BombuscaroI_62	2
1_K558_1_KX108358_BombuscaroI_63	2
1_K559_3_KX108332_BombuscaroI_63	2
1_K560_2_KX108322_BombuscaroI_64s	2
1_K560_3_KX108413_BombuscaroI_64s	2
1_K561_4_KX108345_BombuscaroI_56s	2
1_K562_2_KX108356_BombuscaroI_66	2
1_K563_2_KX108435_BombuscaroI_66	2
1_K563_4_KX108320_BombuscaroI_66	2
1_K564_2_KX108289_BombuscaroI_67_OTU13	2
1_K564_3_KX108315_BombuscaroI_67	2
1_K564_5_KX108408_BombuscaroI_67	2
1_K565_15_KX108368_BombuscaroI_68b	2
1_K567_1_KX108389_BombuscaroI_69	2
1_K567_8_KX108452_BombuscaroI_69	2
1_K568_2_KX108404_BombuscaroI_70	2
1_K568_3_KX108326_BombuscaroI_70	2
1_K569_2_KX108407_BombuscaroI_71	2
1_K569_6_KX108329_BombuscaroI_71	2
1_K570_2_KX108371_BombuscaroI_72	2
1_K571_1_KX108299_BombuscaroI_73	2
1_K571_2_KX108423_BombuscaroI_73	2
1_K571_3_KX108305_BombuscaroI_73	2
1_K572_2_KX108327_BombuscaroI_74	2
1_K572_3_KX108298_BombuscaroI_74	2
1_K572_4_KX108411_BombuscaroI_74	2
1_K572_6_KX108307_BombuscaroI_74	2
1_K573_1_KX108466_BombuscaroII_5	2
1_K573_3_KX108276_BombuscaroII_5	2
1_K574_2_KX108328_BombuscaroI_75	2
1_K575_1_KX108426_BombuscaroI_76	2
1_K575_2_KX108481_BombuscaroI_OTU22	2
1_K576_5_KX108482_BombuscaroII_OTU23	2
1_K577_1_KX108255_BombuscaroII_1b	2
1_K578_1_KX108478_BombuscaroII_2b	2
1_K580_1_KX108457_BombuscaroII_4	2
1_K580_2_KX108384_BombuscaroII_4	2
1_K581_1_KX108267_BombuscaroII_5	2

1_K582_1_KX108456_BombuscaroII_6b	2
1_K582_7_KX108387_BombuscaroII_6b	2
1_K583_5_KX108472_BombuscaroII_6b	2
1_K584_14_KX108277_BombuscaroII_7b	2
1_K585_3_KX108490_BombuscaroII_8	2
1_K585_4_KX108490_BombuscaroII_8	2
1_K590_9_KX108365_BombuscaroII_11b	2
1_K591_10_KX108483_BombuscaroII_OTU23	2
1_K591_15_KX108425_BombuscaroII_12	2
1_K593_7_KX108347_BombuscaroII_13	2
1_K594_3_KX108424_BombuscaroII_14s	2
1_K595_1_KX108342_BombuscaroII_14s	2
1_K596_1_KX108381_BombuscaroII_15g	2
1_K596_2_KX108459_BombuscaroII_15g	2
1_K602_13_KX108428_BombuscaroII_19	2
1_K602_6_KX108343_BombuscaroII_19	2
1_K603_1_KX108268_BombuscaroII_20g	2
1_K603_2_KX108460_BombuscaroII_20g	2
1_K605_4_KX108261_BombuscaroII_22	2
1_K607_1_KX108252_BombuscaroII_24	2
1_K609_1_KX108256_BombuscaroII_26g	2
1_K611_1_KX108467_BombuscaroII_28	2
1_K612_2_KX108382_BombuscaroII_28	2
1_K613_10_KX108464_BombuscaroII_29g	2
1_K613_12_KX108253_BombuscaroII_29g	2
1_K614_12_KX108453_BombuscaroII_30g	2
1_K615_4_KX108312_BombuscaroII_31	2
1_K616_1_KX108279_BombuscaroII_32s	2
1_K617_1_KX108330_BombuscaroII_33g	2
1_K617-8_KX108257_BombuscaroII_33g	2
1_K619_2_KX108285_BombuscaroII_35	2
1_K622_2_KX108436_BombuscaroII_38g	2
1_K622_3_KX108269_BombuscaroII_38g	2
1_K622_4_KX108318_BombuscaroII_38g	2
1_K623_1_KX108406_BombuscaroII_39s	2
1_K623_2_KX108406_BombuscaroII_39s	2
1_K626_13_KX108427_BombuscaroII_41	2
1_K626_2_KX108427_BombuscaroII_41	2
1_K627_10_KX108340_BombuscaroII_42	2
1_K629_6_KX108391_BombuscaroII_44g	2
1_K629-8_KX108258_BombuscaroII_44g	2
1_K630_4_KX108292_BombuscaroII_45g	2
1_K630_6_KX108470_BombuscaroII_45g	2
1_K632_1_KX108377_BombuscaroII_47	2
1_K632_2_KX108273_BombuscaroII_47	2
1_K632_8_KX108295_BombuscaroII_47	2

1_K633_3_KX108310_BombuscaroII_48	2
1_K633_7_KX108474_BombuscaroII_48	2
1_K637_1_KX108286_BombuscaroII_49	2
1_K639_2_KX108262_BombuscaroII_51s	2
1_K639_3_KX108491_BombuscaroII_51s	2
1_K641_2_KX108412_BombuscaroII_53	2
1_K643_11_KX108284_BombuscaroII_55	2
1_K644_3_KX108270_BombuscaroII_56	2
1_K646_1_KX108287_BombuscaroII_56g	2
1_K646_3_KX108309_BombuscaroII_57g	2
1_K647_12_KX108465_BombuscaroII_58g	2
1_K648_1_KX108461_BombuscaroII_59g	2
1_K648_2_KX108385_BombuscaroII_59g	2
1_K650_3_KX108263_BombuscaroII_63	2
1_K650_4_KX108378_BombuscaroII_63	2
1_K651_3_KX108271_BombuscaroII_64_OTU12	2
1_K655_1_KX108264_BombuscaroII_69g	2
1_K655_2_KX108416_BombuscaroII_69g	2
1_K655_5_KX108335_BombuscaroII_69g	2
1_K656_12_KX108417_BombuscaroII_70g	2
1_K656_13_KX108280_BombuscaroII_70g	2
1_K657_15_KX108418_BombuscaroII_71	2
1_K658_3_KX108388_BombuscaroII_72	2
1_K658_6_KX108306_BombuscaroII_72	2
1_K659_1_KX108401_BombuscaroI_25	2
1_K659_2_KX108334_BombuscaroI_25	2
1_K661_7_KX108386_BombuscaroI_1b	2
1_K661_9_KX108454_BombuscaroI_1b	2
1_K663_16_KX108294_BombuscaroI_10b_OTU12	2
1_K664_1_KX108462_BombuscaroI_15g	2
1_K742_4_KX108379_BombuscaroI_03_14	2
1_K742_5_KX108405_BombuscaroI_03_14	2
1_K744_2_KX108336_BombuscaroI_03_14	2
1_K744_6_KX108468_BombuscaroI_03_14	2
1_K745_2_KX108316_BombuscaroI_03_14	2
1_K745_8_KX108442_BombuscaroI_03_14	2
1_K770_7_KX108429_BombuscaroI_03_14	2
1_K774_7_KX108550_BombuscaroI_03_14	2
1_K774_8_KX108400_BombuscaroI_03_14	2
1_K776_8_KX108494_BombuscaroI_03_14	2
1_K777_5_KX108313_BombuscaroI_03_14	2
1_K788_1_KX108493_BombuscaroI_03_14	2
1_K788_2_KX108288_BombuscaroI_03_14	2
1_K788_8_KX108344_BombuscaroI_03_14	2
1_K791_1_KX108492_BombuscaroII_03_14_OTU28	2
1_K791_2_KX108443_BombuscaroII_03_14	2

1_K803_2_KX108430_BombuscaroIII_03_14	2
1_K803_3_KX108302_BombuscaroIII_03_14	2
1_K804_1_KX108399_BombuscaroIII_03_14	2
1_K807_1_KX108283_BombuscaroIII_03_14_OTU12	2
1_K807_2_KX108314_BombuscaroIII_03_14	2
1_K807_4_KX108431_BombuscaroIII_03_14	2
1_K808_1_KX108275_BombuscaroIII_03_14	2
1_K808_4_KX108390_BombuscaroIII_03_14	2
1_K810_2_KX108274_BombuscaroIII_03_14	2
1_K821_3_KX108278_BombuscaroIII_03_14	2
1_K822_3_KX108337_BombuscaroIII_03_14	2
1_K825_2_KX108489_BombuscaroIII_03_14	2
1_K825_3_KX108434_BombuscaroIII_03_14	2
1_K826_1_KX108303_BombuscaroIII_03_14	2
1_K827_1_KX108304_BombuscaroIII_03_14	2
1_K827_2_KX108319_BombuscaroIII_03_14	2
1_K828_1_KX108301_BombuscaroIII_03_14_OTU16	2
1_K828_6_KX108291_BombuscaroIII_03_14_OTU13	2
1_K828_7_KX108297_BombuscaroIII_03_14	2
1_K829_2_KX108293_BombuscaroIII_03_14	2
1_K829_5_KX108338_BombuscaroIII_03_14	2
1_K830_5_KX108438_BombuscaroIII_03_14	2
1_K831_3_KX108359_BombuscaroIII_03_14	2
1_K834_1_KX108361_BombuscaroIII_03_14	2
1_K834_3_KX108300_BombuscaroIII_03_14	2
1_K834_4_KX108300_BombuscaroIII_03_14	2
1_K835_1_KX108339_BombuscaroIII_03_14	2
1_K835_5_KX108451_BombuscaroIII_03_14	2
1_K837_7_KX108445_BombuscaroIII_03_14	2
1_K839_1_KX108317_BombuscaroIII_03_14	2
1_K839_5_KX108410_BombuscaroIII_03_14	2
1_K840_3_KX108370_BombuscaroIII_03_14	2
1_K840_5_KX108440_BombuscaroIII_03_14	2
2_F_EF447237_7.1.3b_F_T2_Alzatea_Glomus5	2
2_F_K109_1_JX296830_F_Q5_Plant17	2
2_F_K114_1_JX296821_K114_1_F_Q5_Plant18	2
2_F_K133_1_DQ336483_F_Q2_Guarea_k	2
2_F_K143_13_DQ336496_F_A_Hyeronima_a	2
2_F_K143_17_DQ336497_F_A_Hyeronima_a	2
2_F_K147_21_DQ336476_F_A_Graffenrieda1	2
2_F_K147_22_EU152135_F_T2_Graffenrieda1	2
2_F_K152_10_JX296759_Fp_Q5_Tabebuia17_OTU16	2
2_F_K159_3_JX296827_Fn_Q5_Tabebuia22_OTU18	2
2_F_K162_4_JX296836_Fn_Q5_Tabebuia23	2
2_F_K168_7_DQ336471_F_A_Clethra	2
2_F_K171_6_DQ336493_F_Q5_Heliocarpus11	2

2_F_K172_10_JX296779_F_T1u_Plant4	2
2_F_K173_2_DQ336522_F_A_Vismia	2
2_F_K173n_4_JX296761_F_T1u_OTU19	2
2_F_K184_3_JX296803_Fp_Q5_Tabebuia	2
2_F_K184_6_JX296797_Fp_Q5_Tabebuia	2
2_F_K188_3_JX296804_Fp_Q5_Tabebuia27	2
2_F_K188_5_JX296832_Fp_Q5_Tabebuia27	2
2_F_K190n_4_JX296807_Fp_Q5_Nectandra4	2
2_F_K192_6_JX296809_Fp_Q5_Nectandra5_OTU17	2
2_F_K22_11_JX296791_Fp_Q5_Cedrela19	2
2_F_K22_2_JX296801_Fp_Q5_Cedrela19	2
2_F_K23_5_JX296799_Fp_Q5_Cedrela20	2
2_F_K23_7_JX296798_Fp_Q5_Cedrela20	2
2_F_K230_5_DQ336521_F_T2_Podocarpus1_OTU24	2
2_F_K235_23_EU152146_F_T2_Podocarpus3	2
2_F_K253_1_EU152151_F_Q2_Campnosperma	2
2_F_K253_7_EU152152_F_Q2_Campnosperma	2
2_F_K255_3_EU152153_F_T2_Podocarpus2_OTU25	2
2_F_K257_3_EU152154_F_Q2_Faramea_OTU19	2
2_F_K260_7_EU152159_F_T2_Prunus_cf_o	2
2_F_K281n_6_KX107935_Fp_Q5_Cedrela	2
2_F_K282n_1_KX107936_Fp_Q5_Cedrela	2
2_F_K298n_2_KX107929_Fn_Q5_Cedrela	2
2_F_K319_7_KX107945_F_T2_Graffenrieda1	2
2_F_K334_2_KX107960_F_Q5_Wurzeln	2
2_F_K399_3_KX107944_F_T2_2550m_Graffenrieda	2
2_F_K427_4_KX107949_F_T2_Wurzeln	2
2_F_K452_1_F_KX107951_T2_2550m_Graffenrieda	2
2_F_K677_2_KX107958_T2_950_OTU24	2
2_F_K680_2_KX107950_T2_950	2
2_F_K682_1_KX107942_T2_950	2
2_F_K692_2_KX107953_T2_950	2
2_F_K704_2_KX107957_T2_950	2
2_F_K708_5_KX107954_T2_550	2
2_F_K710_7_KX107946_T2_550	2
2_F_K727_8_KX107956_T2_250	2
2_F_K730_8_KX107955_T2_250	2
2_F_K731_4_KX107943_T2_250_Graffenrieda	2
2_F_K732_1_KX107952_T2_250	2
2_F_K734_1_KX107959_T2_250	2
2_F_K96_3_JX296789_F_Q5_Plant27	2
3_K634_1_Cajanuma8	2
3_K939_2_Cajanuma_Coreplot_unten	2
35_K1168_9_Tutupali	2
37_K1073_4_Soldados	2
37_K1075_7_Soldados	2

37_K1077_1_Soldados	2
37_K1078_4_Soldados	2
4_K1101_3_Cajas_Paramo	2
4_K1102_6_Cajas_Paramo	2
4_K1104_1_Cajas_Paramo	2
4_K1115_6_Cajas_Paramo	2
4_K1116_1_Cajas_Paramo	2
4_K1135_1_Cajas_Paramo	2
4_K841_11_Cajas_Polylepis1	2
AB555661_Sciaphila_japonica	2
AB594922_Osumunda_japonica_Japan	2
AF213462_Rhizophagus_proliferus_OTU10	2
AJ133706_Sclerocystis_sinuosa	2
AJ699068_Glomus_Marchantia_foliacea_VTX74	2
AJ699070_Glomus_Marchantia_NZ_VTX73	2
EU417581_Glomus_Afrothismia	2
KF386291_Uncultured_Sclerocystis_OTU27	2
1_K498_1_KX108198_BombuscaroI_7s_OTU1	3
1_K501_2_KX108225_BombuscaroI_10s	3
1_K514_1_KX108224_BombuscaroI_19_OTU4	3
1_K519_3_KX108216_BombuscaroI_24	3
1_K520_1_KX108205_BombuscaroI_26	3
1_K535_14_KX108206_BombuscaroI_40	3
1_K545_5_KX108251_BombuscaroI_OTU9	3
1_K547_7_KX108213_BombuscaroI_52	3
1_K550_3_KX108214_BombuscaroI_55	3
1_K553_1_KX108207_BombuscaroI_58g	3
1_K554_4_KX108212_BombuscaroI_59g	3
1_K559_7_KX108250_BombuscaroI_63	3
1_K567_11_KX108210_BombuscaroI_69	3
1_K570_1_KX108208_BombuscaroI_72	3
1_K574_1_KX108200_BombuscaroI_75_OTU7	3
1_K579_1_KX108203_BombuscaroII_3	3
1_K597_8_KX108221_BombuscaroII_16b	3
1_K602_10_KX108219_BombuscaroII_19_OTU7	3
1_K607_7_KX108239_BombuscaroII_24	3
1_K616_2_KX108199_BombuscaroII_32s	3
1_K620_1_KX108240_BombuscaroII_36_OTU8	3
1_K628_9_KX108245_BombuscaroII_43s	3
1_K629_1_KX108241_BombuscaroII_44g	3
1_K640_1_KX108246_BombuscaroII_52g	3
1_K647_2_KX108226_BombuscaroII_58g	3
1_K647_4_KX108215_BombuscaroII_58g	3
1_K648_3_KX108227_BombuscaroII_59g	3
1_K649_1_KX108228_BombuscaroII_60s	3
1_K651_2_KX108247_BombuscaroII_64	3

1_K651_4_KX108217_BombuscaroII_64	3
1_K653_4_KX108201_BombuscaroII_67	3
1_K661_2_KX108209_BombuscaroI_1b	3
1_K662_10_KX108204_BombuscaroI	3
1_K741_2_KX108229_BombuscaroI_03_14	3
1_K742_2_KX108234_BombuscaroI_03_14	3
1_K743_2_KX108232_BombuscaroI_03_14	3
1_K745_1_KX108235_BombuscaroI_03_14	3
1_K747_7_KX108202_BombuscaroI_03_14	3
1_K766_5_KX108230_BombuscaroI_03_14_OTU4	3
1_K777_1_KX108238_BombuscaroI_03_14	3
1_K777_8_KX108220_BombuscaroI_03_14_OTU3	3
1_K778_9_KX108244_BombuscaroI_03_14_OTU8	3
1_K783_5_KX108231_BombuscaroI_03_14	3
1_K784_1_KX108218_BombuscaroI_03_14	3
1_K784_3_KX108233_BombuscaroI_03_14	3
1_K790_7_KX108237_BombuscaroII_OTU5	3
1_K795_7_KX108248_BombuscaroII_03_14	3
1_K797_5_KX108242_BombuscaroII_03_14	3
1_K799_1_KX108249_BombuscaroII_03_14	3
1_K799_5_KX108222_BombuscaroII_03_14	3
1_K802_3_KX108243_BombuscaroII_03_14	3
1_K836_1_KX108236_BombuscaroIII_OTU5	3
1_K837_1_KX108197_BombuscaroIII_03_14_OTU0	3
1_K838_5_KX108211_BombuscaroIII_03_14	3
1_K838_7_KX108223_BombuscaroIII_03_14	3
2_F_K1_5_JX296690_Fp_Q5_Cedrela1	3
2_F_K107_8_JX296685_F_Q5_Plant16	3
2_F_K12_2_JX296714_Fp_Q5_Cedrela5	3
2_F_K127_4_JX296748_F_Q5_Plant19	3
2_F_K128_28_DQ336488_F_Neill_Guarea_OTU9	3
2_F_K128_29_DQ336489_F_Neill_Guarea_p	3
2_F_K130_14_DQ336482_F_Q2_Guarea_k	3
2_F_K146_5_DQ336502_F_Q2_Hyeronima_o	3
2_F_K167_2_DQ336466_F_Q5_Cedrela61	3
2_F_K183_4_JX296857_F_T1o_Plant9	3
2_F_K186n_1_JX296722_Fp_Q5_Nectandra2	3
2_F_K192_7_JX296723_Fp_Q5_Nectandra5	3
2_F_K22_10_JX296742_Fp_Q5_Cedrela19_OTU2	3
2_F_K22_3_JX296724_Fp_Q5_Cedrela19	3
2_F_K228_2_DQ336516_F_Q5_Nectandra7	3
2_F_K23_1_JX296733_Fp_Q5_Cedrela20	3
2_F_K232_1_EU152144_F_T2_Prumnopytis_OTU2	3
2_F_K232_8_EU152145_F_T2_Prumnopytis_OTU6	3
2_F_K24_12_JX296741_Fn_Q5_Cedrela21	3
2_F_K24_7_JX296734_Fn_Q5_Cedrela21	3

2_F_K25_6_JX296725_Fn_Q5_Cedrela22	3
2_F_K26_1_JX296727_F_Tom_Clusia_sp	3
2_F_K280n_2_KX107920_Fp_Q5_Cedrela	3
2_F_K285n_2_KX107914_Fn_Q5_Cedrela	3
2_F_K29_10_JX296696_F_Q5_Cedrela24	3
2_F_K31_9_JX296716_Fn_Q5_Cedrela	3
2_F_K32_9_JX296717_Fn_Q5_Cedrela27	3
2_F_K33_11_JX296758_Fp_Q5_Cedrela28_OTU6	3
2_F_K448_8_KX107911_F_T2_950_OTU0	3
2_F_K45_3_JX296751_Fp_Q5_Cedrela35	3
2_F_K53_3_JX296750_F_Q5_Plant24	3
2_F_K678_9_KX108001_T2_950_OTU35	3
2_F_K683_6_KX107909_T2_950	3
2_F_K684_2_KX107908_T2_950	3
2_F_K688_1_KX107902_T2_950	3
2_F_K691_6_KX108013_T2_950_OTU36	3
2_F_K724_2_KX107906_T2_250	3
2_F_K78_2_JX296719_Fn_Q5_Cedrela50	3
2_F_K81_20_JX296707_Fp_Q5_Cedrela53	3
2_F_K81_3_JX296749_Fp_Q5_Cedrela53	3
2_F_K81_6_JX297756_Fp_Q5_Cedrela53	3
2_F_K95_2_JX396731_F_Q5_Plant26	3
3_K936_4_Cajanuma_Coreplot_unten	3
3_K938_6_Cajanuma_Coreplot_unten	3
3_K950_10_Cajanuma_Coreplot_unten	3
3_K950_9_Cajanuma_Coreplot_unten	3
3_K953_10_Cajanuma_Coreplot_unten	3
3_K969_3_Cajanuma_Coreplot_unten	3
3_K970_14_Cajanuma_Coreplot_unten	3
3_K977_16_Cajanuma_Mirador	3
3_K978_8_Cajanuma_Mirador	3
33_K1160_1_Nero_3300m	3
35_K1166_2_Tutupali	3
37_K1079_1_Soldados	3
37_K1080_2_Soldados	3
37_K1081_2_Soldados	3
37_K1083_3_Soldados	3
37_K1085_4_Soldados	3
4_K1097_3_Cajas_Paramo	3
4_K1237_6_Cajas_Polylepis	3
4_K841_3_Cajas_Polylepis1	3
4_K842_1_Cajas_Polylepis2	3
4_K843_4_Cajas_Polylepis3	3
4_K844_4_Cajas_Polylepis4	3
4_K845_3_Cajas_Polylepis5	3
4_K846_3_Cajas_Polylepis6	3

4_K847_10_Cajas_Polylepis7	3
4_K848_1_Cajas_Polylepis8	3
4_K852_2_Cajas_Polylepis12	3
4_K854_1_Cajas_Calamagrostis	3
4_K855_3_Cajas_Calamagrostis	3
4_K856_2_Cajas_Calamagrostis7	3
4_K856_6_Calamagrostis7_Cajas	3
AJ301859_Rhizophagus_intraradices	3
AJ852530_Glomus_sp_MUCL_43206	3
AY635831_Rhizophagus_intraradices_VTX113	3
EU417622_Glomus_Afrothismia	3
FJ009612_Glomus_irregulare_clone_08	3
FJ009618_Rhizophagus_irregularis_clone_14	3
FJ831530_Glomus_Olea_NF02	3
HE615060_Glomus_VTX312	3
HG004504_Glomus_Sonchus_VTX113	3
JQ811204_Glomus_Preissia_UK_VTX113	3
L20824_Rhizophagus_vesiculiferum	3
X58725_Rhizophagus_intraradices_VTX113	3
Y17640_Glomus_fasciculatum_VTX113	3
35_K1060_5_Tutupali	4
4_K1101_1_Cajas_Paramo	4
4_K1142_3_Cajas_Paramo	4
KY174019_Uncultured_Glomus	4
1_K504_2_KX108557_Bombuscarol_13	5
1_K505_1_KX108532_Bombuscarol_14_OTU54	5
1_K505_2_KX108553_Bombuscarol_14	5
1_K506_1_KX108560_Bombuscarol_15	5
1_K507_1_KX108535_Bombuscarol_16	5
1_K507_2_KX108574_Bombuscarol_16	5
1_K508_2_KX108565_Bombuscarol_17	5
1_K514_2_KX108630_Bombuscarol_OTU70	5
1_K515_1_KX108563_Bombuscarol_20	5
1_K515_2_KX108631_Bombuscarol_OTU70	5
1_K516_2_KX108495_Bombuscarol_21	5
1_K520_4_KX108533_Bombuscarol_26	5
1_K521_4_KX108541_Bombuscarol_27	5
1_K522_2_KX108547_Bombuscarol_28	5
1_K523_3_KX108534_Bombuscarol_29	5
1_K525_9_KX108527_Bombuscarol_31	5
1_K533_10_KX108552_Bombuscarol_38	5
1_K533_14_KX108540_Bombuscarol_38	5
1_K533_9_KX108569_Bombuscarol_38_OTU55	5
1_K548_6_KX108564_Bombuscarol_53g_OTU53	5
1_K555_1_KX108548_Bombuscarol_60	5
1_K560_1_KX108566_Bombuscarol_64s	5

1_K561_2_KX108551_BombuscaroI_65s	5
1_K566_16_KX108567_BombuscaroI_68b_OTU53	5
1_K567_2_KX108568_BombuscaroI_69	5
1_K569_3_KX108571_BombuscaroI_71	5
1_K570_4_KX108536_BombuscaroI_72	5
1_K595_4_KX108562_BombuscaroII_14s	5
1_K604_7_KX108499_BombuscaroII_21s	5
1_K614_9_KX108528_BombuscaroII_30g	5
1_K615_5_KX108632_BombuscaroII_31	5
1_K617_4_KX108497_BombuscaroII_33g	5
1_K643_9_KX108496_BombuscaroII_55_OTU41	5
1_K650_7_KX108530_BombuscaroII_63	5
1_K657_12_KX108537_BombuscaroII_71	5
1_K658_5_KX108573_BombuscaroII_72	5
1_K658_7_KX108546_BombuscaroII_72	5
1_K663_9_KX108498_BombuscaroI_10b	5
1_K768_5_KX108542_BombuscaroI_03_14	5
1_K768_6_KX108570_BombuscaroI_03_14	5
1_K768_7_KX108554_BombuscaroI_03_14	5
1_K769_3_KX108529_BombuscaroI_03_14	5
1_K770_4_KX108538_BombuscaroI_03_14_OTU54	5
1_K770_8_KX108559_BombuscaroI_03_14	5
1_K774_2_KX108550_BombuscaroI_03_14	5
1_K776_4_KX108539_BombuscaroI_03_14	5
1_K791_5_KX108500_BombuscaroII_03_14	5
1_K792_1_KX108492_BombuscaroII_03_14	5
1_K792_2_KX108555_BombuscaroII_03_14	5
1_K803_6_KX108561_BombuscaroIII_03_14	5
1_K805_1_KX108543_BombuscaroIII_03_14	5
1_K805_2_KX108558_BombuscaroIII_03_14	5
1_K828_2_KX108531_BombuscaroIII_03_14	5
1_K829_3_KX108556_BombuscaroIII_03_14	5
1_K829_6_KX108549_BombuscaroIII_03_14	5
1_K832_15_KX108544_BombuscaroIII_03_14	5
1_K839_4_KX108572_BombuscaroIII_03_14_OTU55	5
2_F_EF447220_K1c3_Glomus_Alzatea_VTX191	5
2_F_EF447226_K7c5_F_Alzatea	5
2_F_EF447230_K8c4_F_T2_Alzatea_Glomus3	5
2_F_EF447238_8.2.10_F_T2_Alzatea_Glomus6	5
2_F_K142_4_DQ336499_F_T2_Hyeronima_m	5
2_F_K144_2_EU152134_F_A_Hyeronima_sp	5
2_F_K149_5_DQ336479_F_T2_Graffenrieda2	5
2_F_K150_1_DQ336480_F_A_Graffenrieda3_OTU52	5
2_F_K174n_3_JX296893_F_T1u_Plant6	5
2_F_K181_2_JX296883_T1o_Plant7	5
2_F_K181_8_JX296890_F_T1o_Plant7	5

2_F_K181_9_JX296898_F_T1o_Plant7	5
2_F_K182_1_JX296891_F_T1o_OTU41	5
2_F_K182_9_JX296884_F_T1o_OTU40	5
2_F_K184_2_JX296887_Fp_Tabebuia	5
2_F_K229_2_DQ336474_F_Q3_Clusia_e	5
2_F_K252_1_EU152150_F_T2_Alzatea_OTU52	5
2_F_K260_3_EU152158_F_T2_Prunus_OTU39	5
2_F_K331_9_KX108078_F_Q5_Piper	5
2_F_K423_4_KX108024_F_T2_1250_Wurzeln_OTU39	5
2_F_K427_1_KX108025_F_T2_1250_Wurzeln	5
2_F_K440_2_KX108023_F_T2_2550m_Wurzeln	5
2_F_K443_1_KX108068_F_T2_2500m_Wurzeln	5
2_F_K450_1_KX108036_F_Graffenrieda_T2	5
2_F_K666_1_KX108026_T2_1250	5
2_F_K671_2_KX108074_T2_1250	5
2_F_K677_9_KX108077_T2_950	5
2_F_K678_11_KX108020_T2_950	5
2_F_K678_4_KX108027_T2_950	5
2_F_K679_10_KX108021_T2_950	5
2_F_K681_5_KX108018_T2_950	5
2_F_K681_8_KX108072_T2_950	5
2_F_K682_2_KX108071_T2_950	5
2_F_K702_1_KX108073_T2_950	5
2_F_K705_1_KX108066_T2_950	5
2_F_K707_4_KX108029_T2_550_OTU42	5
2_F_K708_1_KX108070_T2_550	5
2_F_K711_1_KX108076_T2_550	5
2_F_K712_2_KX108019_T2_550	5
2_F_K713_1_KX108035_T2_550	5
2_F_K714_1_KX108032_T2_550	5
2_F_K716_1_KX108030_T2_550	5
2_F_K717_3_KX108034_T2_550	5
2_F_K721_3_KX108075_T2_550	5
2_F_K722_2_KX108028_T2_550	5
2_F_K723_3_KX108031_T2_550_OTU42	5
2_F_K730_3_KX108067_T2_250	5
2_F_K733_6_KX108033_T2_250	5
2_F_K85_4_JX296888_F_Plant25	5
3_14_106_2_Cajanmua_Rynchospora_corymbosa	5
3_14_112_3_Cajanuma_Hypericum2	5
3_14_116_2_Cajanuma_Melastomataceae	5
3_K1192_3_Cajanuma_Paramo	5
3_K1201_1_Cajanuma_Paramo	5
3_K377_2_Cajanuma20	5
3_K379_1_Cajanuma5	5
3_K380_2_Cajanuma6	5

3_K384_6_Cajanuma18	5
3_K394_2_Cajanuma32	5
3_K432_2_Cajanuma40	5
3_K471_1_Cajanuma16	5
3_K472_2_Cajanuma19	5
3_K476_1_Cajanuma13	5
3_K476_7_Cajanuma	5
3_K477_4_Cajanmua	5
3_K478_7_Cajanuma15	5
3_K479_2_Cajanuma18	5
3_K482_2_Cajanuma23	5
3_K483_1_Cajanuma	5
3_K485_1_Cajanuma35	5
3_K486_1_Cajanuma12	5
3_K488_1_Cajanuma38	5
3_K489_7_Cajanuma	5
3_K490_1_Cajanuma41	5
3_K509_1_Cajanuma19	5
3_K511_1_Cajanuma34	5
3_K635_3_Cajanuma17	5
3_K813_5_Cajanuma03_14_3200m	5
3_K908_1_Cajanuma_Coreplot_unten	5
3_K910_1_Cajanuma_Coreplot_unten	5
3_K923_6_Cajanuma_Coreplot_unten	5
3_K929_1_Cajanuma_Coreplot_unten	5
3_K932_6_Cajanuma_Coreplot_unten	5
3_K939_1_Cajanuma_Coreplot_unten	5
3_K939_3_Cajanuma_Coreplot_unten	5
3_K946_9_Cajanuma_Coreplot_unten	5
3_K947_9_Cajanuma_Coreplot_unten	5
3_K949_9_Cajanuma_Coreplot_unten	5
3_K951_9_Cajanuma_Coreplot_unten	5
3_K953_4_Cajanuma_Coreplot_unten	5
3_K955_2_Cajanuma_Mirador	5
3_K956_8_Cajanuma_Mirador	5
3_K968_5_Cajanuma_Mirador	5
3_K974_8_Cajanuma_Mirador	5
3_K978_1_Cajanuma_Mirador	5
3_K978_3_Cajanuma_Mirador	5
3_K981_1_Cajanuma_Mirador	5
3_K985_4_Cajanuma_Mirador	5
33_K1162_2_Nero_3300m	5
35_K1033_1_Tutupali	5
37_K1072_3_Soldados	5
37_K1075_2_Soldados	5
4_K1003_1_Cajas_Polylepis	5

4_K1005_1_Cajas_Polylepis	5
4_K1035_1_Cajas_Polylepis	5
4_K1038_5_Cajas_Polylepis	5
4_K1044_1_Cajas_Polylepis	5
4_K1046_6_Cajas_Polylepis	5
4_K1093_3_Cajas_Paramo	5
4_K1103_2_Cajas_Paramo	5
4_K1106_1_Cajas_Paramo	5
4_K1107_2_Cajas_Paramo	5
4_K1114_3_Cajas_Paramo	5
4_K1130_3_Cajas_Paramo	5
4_K1136_2_Cajas_Paramo	5
4_K1138_6_Cajas_Paramo	5
4_K1142_2_Cajas_Paramo	5
4_K1230_1_Cajas_Polylepis	5
4_K1235_2_Cajas_Polylepis	5
DQ085211_Glomus_JP6	5
DQ396749_Glomus_Podocarpus_VTX191	5
JF414190_Symphyogyna_NewZealand	5
JF414191_Glomeromycota_Symphyogyna	5
2_F_EF447223_K3c6_F_T2_Alzatea_Glomus2	6
2_F_EF447234_K11c8_F_T2_Alzatea_Glomus1	6
2_F_K142_9_DQ336500_F_T2_Hyeronima_m	6
2_F_K149_4_DQ336478_F_T2_Graffenrieda2_OTU68	6
2_F_K150_3_DQ336481_F_A_Graffenrieda3	6
2_F_K172_11_JX296914_T1u_Plant4	6
2_F_K174n_1_JX296913_F_T1u_Plant6	6
2_F_K174n_6_JX296939_F_T1u_Plant6	6
2_F_K438_5_KX108113_F_T2_2550m_Wurzeln	6
2_F_K440_8_KX108084_F_T2_2550m_OTU57	6
2_F_K444_1_KX108111_F_T2_2550m_Wurzeln	6
2_F_K447_1_KX108109_F_T2_1250_OTU68	6
2_F_K450_3_KX108079_F_T2_950_Graffenrieda	6
2_F_K450_4_KX108114_F_T2_950_Graffenrieda	6
2_F_K455_2_KX108080_F_T2_2550m_Plant	6
2_F_K455_8_KX108110_T2_2500m	6
2_F_K460_1_KX108081_F_T2_2550m_Graffenrieda_h	6
2_F_K677_10_KX108082_T2_950_OTU57	6
2_F_K702_2_KX108085_T2_950	6
2_F_K705_2_KX108115_T2_950	6
2_F_K711_3_KX108086_T2_550	6
2_F_K721_1_KX108112_T2_550	6
2_F_K722_3_KX108083_T2_550	6
3_14_112_1_Cajanuma_Hypericum2	6
1_K597_6_KX108637_BombuscaroII_16b	7
1_K598_6_KX108634_BombuscaroII_17	7

1_K600_12_KX108638_BombuscaroII_18_OTU72	7
1_K602_9_KX108635_BombuscaroII_19	7
1_K614_11_KX108639_BombuscaroII_30g	7
1_K628_1_KX108633_BombuscaroII_43s	7
1_K662_12_KX108636-Bombuscarol_3	7
1_K795_1_KX108640_BombuscaroII_03_14	7
2_F_K108_3_KX108120_F_Q5_Roots	7
2_F_K128n_12_JX297009_F_Q5_OTU72	7
2_F_K128n_7_JX297012_F_Q5_OTU73	7
2_F_K129_22_EU152132_F_Q2_Guarea_sp	7
2_F_K17_7_JX297010_F_Q5_Cedrela14	7
2_F_K334_7_KX108121_F_Q5_Plant	7
2_F_K78_1_JX297008_Fn_Q5_Cedrela50	7
2_F_K96_2_JX297011_F_Q5_OTU73	7
1_K528_1_KX108516_Bombuscarol_33	8
1_K531_5_KX108517_Bombuscarol_36_OTU48	8
1_K536_4_KX108502_Bombuscarol_41g	8
1_K566_9_KX108501_Bombuscarol_68b_OTU44	8
1_K577_6_KX108509_BombuscaroII_1b	8
1_K578_5_KX108508_BombuscaroII_2b	8
1_K578_7_KX108518_BombuscaroII_2b	8
1_K583_1_KX108504_BombuscaroII_6b	8
1_K584_1_KX108506_BombuscaroII_7b_OTU47	8
1_K586_2_KX108511_BombuscaroII_9b	8
1_K603_7_KX108512_BombuscaroII_20g	8
1_K617_2_KX108505_BombuscaroII_33g	8
1_K618_1_KX108513_BombuscaroII_34	8
1_K620_2_KX108515_BombuscaroII_36	8
1_K621_4_KX108503_BombuscaroII_37s	8
1_K630_8_KX108514_BombuscaroII_45g	8
1_K662_14_KX108507_Bombuscarol_3	8
1_K794_1_KX108510_BombuscaroII_03_14	8
2_F_K1_1_JX2969181_Fp_Q5_Cedrela1	8
2_F_K128n_11_JX296929_F_Q5_Plant20	8
2_F_K129_14_DQ336490_F_Q2_Guarea_sp	8
2_F_K132_10_DQ336487_F_Q2_Guarea	8
2_F_K144_10_DQ336504_F_A_Hyeronima_OTU44	8
2_F_K162_1_JX296926_Fn_Q5_Tabebuia23	8
2_F_K173_4_DQ336523_F_A_Vismia	8
2_F_K23_6_JX296923_Fp_Q5_Cedrela20_OTU46	8
2_F_K24_15_JX296924_Fn_Q5_Cedrela21	8
2_F_K257_5_EU152155_F_Q2_Faramea	8
2_F_K258_4_EU152156_F_A_Critoniopsis	8
2_F_K259_1_EU152157_F_Q2_Miconia	8
2_F_K280n_7_KX108045_Fp_Q5_Cedrela_OTU45	8
2_F_K281n_1_KX107900_Fp_Q5_Cedrela_OTU45	8

2_F_K37_13_JX296916_F_Q5_Plant21	8
2_F_K38_11_JX296915_F_Q5_Plant22	8
2_F_K448_1_KX108057_F_T2_950_Plant	8
2_F_K448_5_KX108060_F_T2_950	8
2_F_K690_1_KX108061_T2_950	8
2_F_K690_5_KX108058_T2_950_OTU48	8
2_F_K78_10_JX296917_Fn_Q5_Cedrela50	8
3_K429_1_Cajanuma45	8
3_K491_1_Cajanuma44	8
3_K492_1_Cajanuma45	8
3_K947_10_Cajanuma_Coreplot_unten	8
37_K1075_5_Soldados	8
37_K1076_2_Soldados	8
37_K1078_2_Soldados	8
37_K1079_2_Soldados	8
37_K1081_1_Soldados	8
37_K1082_2_Soldados	8
37_K1083_4_Soldados	8
37_K1084_1_Soldados	8
37_K1086_6_Soldados	8
37_K1247_7_Soldados	8
AB556919_Sciaphila_tosaensis	8
AB556928_Sciaphila_tosaensis	8
AJ699069_Gl_Marchantia_foliacea	8
AY903734_Glomus_Botrychium	8
DQ396707_Glomus_PF11	8
EU417640_Glomus_Sciaphila_VT166	8
HE613472_Uncultured_Glomus_partial_VT174	8
KC708343_Phaeoceros_carolinianus	8
KC708376_Phaeoceros_laevis	8
1_K619_5_KX108519_BombuscaroII_35	9
1_K629_2_KX108526_BombuscaroII_44g	9
1_K640_5_KX108520_BombuscaroII_52g_OTU51	9
1_K641_1_KX108525_BombuscaroII_53	9
1_K786_1_KX108521_BombuscaroI_03_14	9
1_K790_1_KX108524_BombuscaroII_03_14	9
1_K836_2_KX108522_BombuscaroIII_03_14_OTU51	9
1_K838_2_KX108523_BombuscaroIII_03	9
1_K498_2_KX108600-BombuscaroI_7s	10
1_K501_1_KX108603_BombuscaroI_10s_OTU60	10
1_K503_2_KX108584_BombuscaroI_12	10
1_K519_1_KX108594_BombuscaroI_24_OTU58	10
1_K520_2_KX108588_BombuscaroI_26	10
1_K530_2_KX108597_BombuscaroI_35	10
1_K535_11_KX108595_BombuscaroI_40	10
1_K536_5_KX108599_BombuscaroI_41g	10

1_K538_4_KX108598_BombuscaroI_43	10
1_K540_2_KX108587_BombuscaroI_45	10
1_K542_7_KX108593_BombuscaroI_47	10
1_K543_12_KX108576_BombuscaroI_48	10
1_K544_10_KX108592_BombuscaroI_49	10
1_K547_1_KX108575_BombuscaroI_52	10
1_K551_1_KX108589_BombuscaroI_56	10
1_K566_11_KX108608_BombuscaroI_OTU61	10
1_K572_1_KX108604_BombuscaroI_74	10
1_K577_2_KX108622_BombuscaroII_1b_OTU63	10
1_K585_2_KX108623_BombuscaroII_8	10
1_K586_4_KX108618_BombuscaroII_9b	10
1_K593_1_KX108580_BombuscaroII_13	10
1_K593_2_KX108619_BombuscaroII_13	10
1_K594_5_KX108610_BombuscaroII_14s	10
1_K599_3_KX108624_BombuscaroII_OTU66	10
1_K600_9_KX108628_BombuscaroII_18_OTU67	10
1_K605_1_KX108585_BombuscaroII_22_OTU69	10
1_K606_1_KX108586_BombuscaroII_23	10
1_K606_2_KX108612_BombuscaroII_23_OTU61	10
1_K606_5_KX108614_BombuscaroII_23_OTU62	10
1_K610_1_KX108615_BombuscaroII_27s	10
1_K612_1_KX108620_BombuscaroII_28	10
1_K619_7_KX108581_BombuscaroII_35	10
1_K620_7_KX108626_BombuscaroII_36	10
1_K621_1_KX108627_BombuscaroII_37s	10
1_K624_7_KX108606_BombuscaroII_39s	10
1_K626_9_KX108582_BombuscaroII_41	10
1_K638_11_KX108629_BombuscaroII_50_OTU67	10
1_K638_8_KX108625_BombuscaroII_OTU66	10
1_K642_1_KX108579_BombuscaroII_54s	10
1_K643_13_KX108607_BombuscaroII_OTU61	10
1_K643_4_KX108616_BombuscaroII_55	10
1_K644_4_KX108609_BombuscaroII_56	10
1_K644_6_KX108621_BombuscaroII_56	10
1_K652_1_KX108613_BombuscaroII_66s	10
1_K652_7_KX108611_BombuscaroII_66s	10
1_K653_2_KX108601_BombuscaroII_67_OTU60	10
1_K654_4_KX108617_BombuscaroII_68	10
1_K656_2_KX108605_BombuscaroII_70g	10
1_K659_4_KX108590_BombuscaroI_25	10
1_K750_13_KX108602_BombuscaroI_03_14	10
1_K791_3_KX108583_BombuscaroII_03_14	10
1_K820_3_KX108591_BombuscaroIII_03_14_OTU58	10
1_K822_2_KX108596_BombuscaroIII_03_14	10
1_K831_4_KX108577_BombuscaroIII_03_14	10

1_K833_3_KX108578_BombuscaroIII_03_14	10
2_F_K1_12_JX296943_Fp_Q5_Cedrela1_OTU69	10
2_F_K1_3_JX296974_Fp_Cedrela1	10
2_F_K106_2_JX296959_Fp_Plant15	10
2_F_K106_4_JX296954_F_Q5_Plant15	10
2_F_K107_5_JX296983_F_Plant16	10
2_F_K12_3_JX296945_Fp_Cedrela5	10
2_F_K127_12_JX296980_F_Plant19	10
2_F_K127_15_JX296951_F_Plant19	10
2_F_K131_11_DQ336485_F_Guarea_cf_k	10
2_F_K151_3_JX296986_Fp_Tabebuia16	10
2_F_K152_1_JX2970071_Fp_Tabebuia17	10
2_F_K152_2_JX296952_Fp_Q5_Tabebuia17	10
2_F_K153_5_JX296950_Fp_Tabebuia18	10
2_F_K153_6_JX296995_Fp_Tabebuia18	10
2_F_K170_7_DQ336508_F_Q2_Inga3	10
2_F_K174_2_DQ336524_F_Vismia	10
2_F_K175_7_DQ336514_F_Q5_Nectandra6	10
2_F_K181_5_JX296975_F_T1o_Plant7	10
2_F_K185n_3_JX296969_Fp_Nectandra1	10
2_F_K187n_2_JX296948_Fp_Q5_Tabebuia26	10
2_F_K189_4_DQ336507_F_Inga2_OTU64	10
2_F_K189n_3_JX296978_Fp_Nectandra3	10
2_F_K190_1_DQ336505_F_Inga1	10
2_F_K23_4_JX296968_Fp_Cedrela20	10
2_F_K243_1_KX108102_F_Hyeronima	10
2_F_K25_5_JX296947_Fn_Cedrela22	10
2_F_K280n_4_KX108106_Fp_Cedrela_OTU62	10
2_F_K280n_6_KX108045_Fp_Cedrela	10
2_F_K32_10_JX296953_Fn_Q5_Cedrela27	10
2_F_K32_11_JX296966_Fn_Cedrela27	10
2_F_K320_1_KX108089_F_Graffenrieda	10
2_F_K334_5_KX108090_F_Q5	10
2_F_K37_10_JX296946_F_Q5_Plant21	10
2_F_K38_13_JX296958_Fp_Plant22	10
2_F_K39_11_JX296982_F_Plant28	10
2_F_K43_8_JX296965_Fn_Cedrela33	10
2_F_K53_7_JX296967_F_Plant24	10
2_F_K78_11_JX297001_Fn_Cedrela50_OTU59	10
2_F_K78_4_JX297019_Fn_Q5_Cedrela50	10
2_F_K79_9_JX296985_Fn_Cedrela51	10
2_F_K80_6_JX296955_Fp_Cedrela52	10
2_F_K81_19_JX296960_Fp_Cedrela53	10
2_F_K81_22_JX296984_Fp_Cedrela53	10
2_F_K95_4_JX296961_F_Plant26	10
AB594911_Osumunda_japonica_Japan	10

FJ831546_Glomus_535_PFB_NF06	10
GQ140621_Glomus_Perilla_China	10
JN644467_apple_tree_Halle	10
2_F_K128n_10_JX297017_F_Q5_OTU76	11
2_F_K17_6_JX297016_F_Q5_Cedrela14_OTU76	11
2_F_K187n_5_JX297015_Fp_Q5_Tabebuia26_OTU75	11
2_F_K262_7_KX108122_F_Q5_Baum00025	11
AJ245637_Funneliformis_geosporum_OTU74	11
AJ276085_Funneliformis_fragilistratum	11
AJ276086_Funneliformis_coronatum	11
AJ301858_Funneliformis_verruculosum	11
AY635833_Funneliformis_mosseae	11
DQ085256_Glomus_JP4_OTU75	11
Y17653_Funneliformis_caledonium_OTU74	11
1_K503_1_KX108650_Bombuscarol_12	12
1_K565_16_KX108651_Bombuscarol_68	12
2_F_K109_8_JX297056_F_Q5_Plant17	12
2_F_K151_2_JX297055_Fp_Q5_Tabebuia16_OTU82	12
2_F_K152_3_JX297058_Fp_Q5_Tabebuia17	12
2_F_K153_10_JX297053_Fp_Q5_Tabebuia18	12
2_F_K24_13_JX297054_Fn_Q5_Cedrela21	12
2_F_K284n_8_KX108149_Fn_Q5_Cedrela_OTU82	12
2_F_K314n_1_KX108150_Fp_Q5_Tabebuia	12
2_F_K39_1_JX297052_Fp_Q5_Plant28	12
2_F_K399_6_KX108151_F_T2_2550m_Graffenrieda	12
2_F_K81_1_JX297051_Fp_Q5_Cedrela53	12
2_F_K96_4_JX297057_F_Q5_Plant27_OTU83	12
3_K290_1_Cajanuma3	12
3_K374_1_Cajanuma2	12
3_K481_2_Cajanuma22	12
3_K915_4_Cajanuma_Coreplot_unten	12
3_K934_13_Cajanuma_Coreplot_unten	12
3_K936_6_Cajanuma_Coreplot_unten	12
3_K937_3_Cajanuma_Coreplot_unten	12
3_K940_2_Cajanuma_Coreplot_unten	12
3_K945_12_Cajanuma_Coreplot_unten	12
3_K951_16_Cajanuma_Coreplot_unten	12
35_K1059_1_Tutupali	12
35_K1059_2_Tutupali	12
35_K1060_2_Tutupali	12
35_K1061_1_Tutupali	12
35_K1062_3_Tutupali	12
37_K1072_2_Soldados	12
4_K1088_2_Cajas_Paramo	12
4_K1089_1_Cajas_Paramo	12
4_K1091_2_Cajas_Paramo	12

4_K1092_1_Cajas_Paramo	12
4_K1095_6_Cajas_Paramo	12
4_K1096_1_Cajas_Paramo	12
4_K1108_5_Cajas_Paramo	12
4_K1109_3_Cajas_Paramo	12
4_K1110_8_Cajas_Paramo	12
4_K1111_4_Cajas_Paramo	12
4_K1112_1_Cajas_Paramo	12
4_K1113_1_Cajas_Paramo	12
4_K1121_5_Cajas_Paramo	12
4_K1122_1_Cajas_Paramo	12
EF136887_Acaulospora_sp_OTU84	12
FR750213_Acaulospora_sp_Att1186_5	12
JF414186_Symphyogyna_Australia	12
JN252439_Uncultured_Acaulosporaceae_sand_dune_	12
1_K498_6_KX108666_BombuscaroI_7s	13
1_K499_8_KX108652_BombuscaroI_8s	13
1_K513_5_KX108654_BombuscaroI_18	13
1_K524_1_KX108660_BombuscaroI_30	13
1_K530_1_KX108653_BombuscaroI_35	13
1_K531_2_KX108658_BombuscaroI_36	13
1_K537_4_KX108661_BombuscaroI_42	13
1_K539_1_KX108662_BombuscaroI_44_OTU88	13
1_K539_2_KX108679_BombuscaroI_44_OTU94	13
1_K540_5_KX108663_BombuscaroI_45	13
1_K543_11_KX108664_BombuscaroI_48	13
1_K555_2_KX108673_BombuscaroI_50_OTU86	13
1_K560_6_KX108655_BombuscaroI_64s	13
1_K565_10_KX108665_BombuscaroI_68b	13
1_K569_4_KX108680_BombuscaroI_71	13
1_K574_3_KX108681_BombuscaroI_75_OTU94	13
1_K590_14_KX108674_BombuscaroII_11b_OTU90	13
1_K591_5_KX108667_BombuscaroII_12	13
1_K591_9_KX108365_BombuscaroII_12_OTU89	13
1_K594_7_KX108678_BombuscaroII_14s	13
1_K608_2_KX108670_BombuscaroII_25b	13
1_K620_6_KX108671_BombuscaroII_36	13
1_K625_10_KX108656_BombuscaroII_40_OTU85	13
1_K638_16_KX108676_BombuscaroII_50	13
1_K638_5_KX108659_BombuscaroII_50	13
1_K788_4_KX108657_BombuscaroI_03_14	13
1_K791_4_KX108669_BombuscaroII_03_14	13
1_K823_2_KX108672_BombuscaroIII_03_14	13
1_K825_1_KX108677_BombuscaroIII_03_14	13
2_F_K127_1_JX297072_F_Q5_Plant19	13
2_F_K152_6_JX297025_Fp_Q5_Tabebuia17	13

2_F_K185n_5_JX297044_Fp_Q5_Nectandra1	13
2_F_K228n_1_KX108153_Fp_Q5_Tabebuia_OTU85	13
2_F_K228n_4_KX108161_Fp_Q5_Tabebuia	13
2_F_K24_1_JX297020_Fn_Q5_Cedrela21	13
2_F_K24_3_KX108169_Fn_Q5_Cedrela21_OTU87	13
2_F_K24_4_JX297027_Fn_Q5_Cedrela21	13
2_F_K241_14_KX108165_Fn_Q5_Cedrela	13
2_F_K315_1_KX108158_Fp_Q5_Tabebuia	13
2_F_K32_5_JX297022_Fn_Q5_Cedrela27	13
2_F_K320_8_KX108163_F_T2_Graffenrieda2	13
2_F_K35_1_AY394664_F_T2_Graffenrieda2_Acaulosp	13
2_F_K367_3_KX108160_Fp_Q5_Tabebuia	13
2_F_K38_14_JX297024_F_Q5_Plant22	13
2_F_K39_2_JX297032_F_Q5_Plant28	13
2_F_K45_1_JX297028_Fp_Q5_Cedrela35	13
2_F_K725_8_KX108156_T2_250	13
2_F_K726_2_KX108166_T2_250	13
2_F_K728_1_KX108159_T2_250	13
2_F_K736_2_KX108162_T2_250	13
2_F_K78_5_JX297021_Fn_Q5_Cedrela50	13
2_F_K85_1_JX297023_F_Q5_Plant25	13
3_K374_7_Cajanuma2	13
3_K474_2_Cajanuma22	13
3_K486_2_Cajanuma12	13
3_K492_7_Cajanuma45	13
FJ009670_Acaulospora_mellea	13
HE610427_Acaulospora_lacunosa	13
2_F_K184_15_JX297059_Fp_Q5_Tabebuia	14
2_F_K45_2_JX297049_Fp_Q5_Cedrela35_OTU81	14
3_K1195_4_Cajanuma_Paramo	14
3_K388_2_Cajanuma26	14
3_K412_2_P63_Cajanuma44	14
3_K429_3_Cajanuma45	14
3_K471_2_Cajanuma16	14
3_K474_4_Cajanuma22	14
3_K476_3_Cajanuma13	14
3_K478_1_Cajanuma15	14
3_K481_6_Cajanuma22	14
3_K483_4_Cajanuma26	14
3_K811_2_Cajanuma03_14_3200m	14
3_K917_4_Cajanuma_Coreplot_unten	14
3_K925_1_Cajanuma_Coreplot_unten	14
3_K926_11_Cajanuma_Coreplot_unten	14
3_K927_9_Cajanuma_Coreplot_unten	14
3_K929_3_Cajanuma_Coreplot_unten	14
3_K933_4_Cajanuma_Coreplot_unten	14

3_K937_6_Cajanuma_Coreplot_unten	14
3_K943_5_Cajanuma_Coreplot_unten	14
3_K944_11_Cajanuma_Coreplot_unten	14
3_K947_16_Cajanuma_Coreplot_unten	14
33_K1155_2_Nero_3300m	14
33_K1156_2_Nero_3300m	14
33_K1159_5_Nero_3300m	14
33_K1161_1_Nero_3300m	14
33_K1162_3_Nero_3300m	14
33_K1163_1_Nero_3300m	14
35_K1026_1_Tutupali	14
35_K1029_2_Tutupali	14
35_K1030_2_Tutupali	14
35_K1031_2_Tutupali	14
35_K1033_7_Tutupali	14
35_K1053_2_Tutupali	14
35_K1054_1_Tutupali	14
35_K1056_1_Tutupali	14
35_K1061_8_Tutupali	14
35_K1167_1_Tutupali	14
35_K1169_2_Tutupali	14
4_K1003_3_Cajas_Polylepis	14
4_K1035_3_Cajas_Polylepis	14
4_K1038_3_Cajas_Polylepis	14
4_K1038_4_Cajas_Polylepis	14
4_K1039_1_Cajas_Polylepis	14
4_K1042_1_Cajas_Polylepis	14
4_K1044_3_Cajas_Polylepis	14
4_K1044_6_Cajas_Polylepis	14
4_K1045_1_Cajas_Polylepis	14
4_K1234_1_Cajas_Polylepis	14
KF386272_Acaulospora_VTX30	14
Y17633_Acaulospora_laevis	14
1_K516_8_KX108641_BombuscaroI_21	15
1_K517_3_KX108649_BombuscaroI_22	15
1_K518_4_KX108642_BombuscaroI_23b	15
1_K537_1_KX108643_BombuscaroI_42_OTU77	15
1_K540_6_KX108644_BombuscaroI_45	15
1_K578_3_KX108645_BombuscaroII_2b	15
1_K579_7_KX108647_BombuscaroII_3_OTU80	15
1_K608_1_KX108648_BombuscaroII_25b	15
1_K661_12_KX108646_BombuscaroI_1b	15
2_F_EF447239_K10c4_F_T2_Alzatea	15
2_F_EF447241_K15c1_F_T2_Uncultured_Acaulospora	15
2_F_EF447243_K16c1_F_T2_Alzatea_Acaulospora_Ad	15
2_F_EF447244_5.4.1_F_T2_Alzatea	15

2_F_EF447245_7.1.3_F_T2_Alzatea	15
2_F_K172_14_JX297061_F_T1u_Plant4	15
2_F_K26_4_JX297066_F_Tom_Clusia_sp	15
2_F_K262_2_KX108148_F_Q5_Baum	15
2_F_K400_2_KX108145_F_T2_2550m_Graffenrieda_C	15
2_F_K443_6_F_KX108137_T2_2550m_Wurzeln	15
2_F_K451_4_F_KX108126_T2_2550m_Podocarpus	15
2_F_K458_1_KX108125_F_T2_2550m_Plant	15
2_F_K459_2_KX108140_F_T2_2550m_Clusia	15
2_F_K667_8_KX108132_T2_1250	15
2_F_K669_7_KX108142_T2_1250	15
2_F_K670_8_KX108124_T2_1250_OTU77	15
2_F_K670_9_KX108134_T2_1250	15
2_F_K671_1_KX108138_T2_1250	15
2_F_K674_4_KX108141_T2_1250	15
2_F_K676_2_KX108143_T2_1250	15
2_F_K703_1_KX108135_T2_950	15
2_F_K704_5_KX108123_T2_950	15
2_F_K710_1_KX108139_T2_550	15
2_F_K710_2_KX108129_T2_550	15
2_F_K711_2_KX108130_T2_550	15
2_F_K715_5_KX108136_T2_550	15
3_14_107_1_Cajanuma_Chusquea	15
3_14_110_1_Cajanuma_Eriocaulon_microcephalum	15
3_14_111_1_Cajanuma_Hypericum1	15
3_K1187_1_Cajanuma_Paramo	15
3_K1189_5_Cajanuma_Paramo	15
3_K1195_2_Cajanuma_Paramo	15
3_K1196_2_Cajanuma_Paramo	15
3_K1197_1_Cajanuma_Paramo	15
3_K1199_1_Cajanuma_Paramo	15
3_K1202_6_Cajanuma_Paramo	15
3_K378_2_Cajanuma24	15
3_K380_1_Cajanuma6	15
3_K381_1_Cajanuma7	15
3_K381_4_Cajanuma7	15
3_K387_1_Cajanuma25	15
3_K388_1_Cajanuma26	15
3_K392_1_Cajanuma30	15
3_K397_8_Cajanuma40	15
3_K398_2_Cajanuma41	15
3_K403_1_Cajanuma36	15
3_K406_6_Cajanuma37	15
3_K407_5_Cajanuma39	15
3_K430_2_Cajanuma45	15
3_K472_1_Cajanuma19	15

3_K475_6_Cajanuma23	15
3_K477_2_Cajanuma14	15
3_K487_1_Cajanuma36	15
3_K489_1_Cajanuma40	15
3_K489_2_Cajanuma40	15
3_K634_3_Cajanuma8	15
3_K813_1_Cajanuma03_14_3200m	15
3_K917_1_Cajanuma_Coreplot_unten	15
3_K920_14_Cajanuma_Coreplot_unten	15
3_K931_2_Cajanuma_Coreplot_unten	15
3_K934_1_Cajanuma_Coreplot_unten	15
3_K945_10_Cajanuma_Coreplot_unten	15
3_K950_15_Cajanuma_Coreplot_unten	15
3_K954_2_Cajanuma_Mirador	15
3_K956_1_Cajanuma_Mirador	15
3_K956_3_Cajanuma_Mirador	15
3_K958_3_Cajanuma_Mirador	15
3_K959_4_Cajanuma_Mirador	15
3_K961_5_Cajanuma_Mirador	15
3_K962_1_Cajanuma_Mirador	15
3_K963_1_Cajanuma_Mirador	15
3_K963_2_Cajanuma_Mirador	15
3_K964_3_Cajanuma_Mirador	15
3_K965_1_Cajanuma_Mirador	15
3_K967_15_Cajanuma_Mirador	15
3_K968_12_Cajanuma_Mirador	15
3_K970_4_Cajanuma_Coreplot_unten	15
3_K975_7_Cajanuma_Mirador	15
3_K981_4_Cajanuma_Mirador	15
3_K983_3_Cajanuma_Mirador	15
3_K987_4_Cajanuma_Mirador	15
3_K989_2_Cajanuma_Mirador	15
33_K1162_1_Nero_3300m	15
33_K1163_2_Nero_3300m	15
33_K1164_2_Nero_3300m	15
35_K1031_1_Tutupali	15
35_K1032_1_Tutupali	15
35_K1032_3_Tutupali	15
35_K1033_2_Tutupali	15
35_K1064_1_Tutupali	15
35_K1167_2_Tutupali	15
4_K1002_2_Cajas_Polylepis	15
4_K1004_1_Cajas_Polylepis	15
4_K1036_1_Cajas_Polylepis	15
4_K1040_1_Cajas_Polylepis	15
4_K1087_4_Cajas_Paramo	15

4_K1093_2_Cajas_Paramo	15
4_K1094_2_Cajas_Paramo	15
4_K1098_1_Cajas_Paramo	15
4_K1099_2_Cajas_Paramo	15
4_K1100_3_Cajas_Paramo	15
4_K1101_2_Cajas_Paramo	15
4_K1104_6_Cajas_Paramo	15
4_K1107_1_Cajas_Paramo	15
4_K1114_4_Cajas_Paramo	15
4_K1115_1_Cajas_Paramo	15
4_K1116_2_Cajas_Paramo	15
4_K1117_1_Cajas_Paramo	15
4_K1117_2_Cajas_Paramo	15
4_K1119_1_Cajas_Paramo	15
4_K1120_1_Cajas_Paramo	15
4_K1129_5_Cajas_Paramo	15
4_K1132_7_Cajas_Paramo	15
4_K1133_4_Cajas_Paramo	15
4_K1134_1_Cajas_Paramo	15
4_K1135_7_Cajas_Paramo	15
4_K1136_1_Cajas_Paramo	15
4_K1137_3_Cajas_Paramo	15
4_K1137_7_Cajas_Paramo	15
4_K1138_3_Cajas_Paramo	15
4_K1141_2_Cajas_Paramo	15
4_K1141_6_Cajas_Paramo	15
4_K1142_1_Cajas_Paramo	15
4_K1143_1_Cajas_Paramo	15
4_K1144_1_Cajas_Paramo	15
4_K1144_5_Cajas_Paramo	15
4_K1148_2_Cajas_Paramo	15
4_K1149_2_Cajas_Paramo	15
4_K1152_1_Cajas_Paramo	15
4_K1229_3_Cajas_Polylepis	15
4_K1231_2_Cajas_Polylepis	15
4_K1238_1_Cajas_Polylepis	15
AJ306440_Acaulospora_W3424	15
FN825899_Acaulospora_brasiliensis_VTX230_OTU78	15
1_K579_5_KX108682_BombuscaroII_3_OTU96	16
2_F_K43_10_JX297075_Fn_Q5_Cedrela33_OTU96	16
37_K1081_4_Soldados	16
FR686954_Diversispora_spurca	16
1_K599_8_KX108684_BombuscaroII_18_OTU97	17
1_K612_4_KX108683_BombuscaroII_28	17
1_K628_11_KX108685_BombuscaroII_43s	17
1_K631_4_KX108686_BombuscaroII_46_OTU97	17

1_K516_1_KX108690_BombuscaroI_21_OTU112	18
1_K626_16_KX108692_BombuscaroII_41_OTU115	18
1_K651_5_KX108693_BombuscaroII_64	18
1_K830_7_KX108691_BombuscaroIII_03_14	18
1_K840_2_KX108694_BombuscaroIII_03_14	18
2_F_EF447242_K15c2_F_T2_Alzatea_Uncultured_Giga	18
2_F_K109_3_JX297091_F_Q5_Plant17_OTU113	18
2_F_K164_4_KX108188_FW	18
2_F_K172_12_JX297090_F_T1u_Plant4	18
2_F_K180n_5_KX108189_FW	18
2_F_K207n_2_KX108190_FW	18
2_F_K22_1_JX297088_Fp_Q5_Cedrela19_OTU112	18
2_F_K29_4_JX297083_F_Q5_Cedrela24	18
2_F_K319_1_KX108194_F_T2_Graffenrieda1_OTU117	18
3_K480_3_Cajanuma22	18
3_K937_5_Cajanuma_Coreplot_unten	18
AJ276092_Scutellospora_auriglоba	18
AJ852603_Gigaspora_margarita	18
FJ009672_Scutellospora_calospora	18
HF968771_Gigaspora_margarita	18
HQ202289_Scutellospora_crenulata	18
Z14010_Gigaspora_gigantea	18
2_F_K192_1_JX297079_Fp_Q5_Nectandra5_OTU99	19
2_F_K23_9_JX297081_Fp_Q5_Cedrela20	19
2_F_K262_4_KX108173_F_Q5	19
2_F_K37_16_JX297077_F_Q5_Plant21	19
2_F_K81_2_JX297080_Fp_Q5_Cedrela53	19
3_K933_8_Cajanuma_Coreplot_unten	19
3_K938_10_Cajanuma_Coreplot_unten	19
3_K942_10_Cajanuma_Coreplot_unten	19
3_K950_16_Cajanuma_Coreplot_unten	19
35_K1064_3_Tutupali	19
37_K1080_8_Soldados	19
37_K1083_2_Soldados	19
37_K1085_1_Soldados	19
4_K1005_2_Cajas_Polylepis	19
4_K1034_2_Cajas_Polylepis	19
4_K1041_1_Cajas_Polylepis	19
4_K1046_1_Cajas_Polylepis	19
4_K1048_3_Cajas_Polylepis	19
AJ276075_Claroideoglomus_claroideum_OTU98	19
AJ276083_Claroideoglomus_lamellosum_OTU98	19
KC708365_Claroideoglomus_Megaceros_NZ_VTX193	19
Y17639_Claroideoglomus_etunicatum_OTU98	19
37_K1078_1_Soldados	20
2_F_K25_9_JX297082_Fn_Q5_Cedrela22_OTU101	21

2_F_K284n_1_KX108174_Fn_Q5_Cedrela	21
2_F_K665_1_KX108175_T2_1250_OTU101	21
37_K1079_7_Soldados	21
X58724_Endogone_pisiformis	22
AJ301862_Paraglomus_brasilianum	23
NG017179_Paraglomus_occultum	23
1_K532_1_KX108688_Bombuscarol_37_OTU109	24
1_K638_6_KX108687_BombuscaroII_50_OTU102	24
2_F_K109_16_JX297093_F_Q5_Plant17	24
2_F_K168n_1_KX108186_FW	24
2_F_K168n_5_KX108183_FW	24
2_F_K184_5_JX297100_Fp_Q5_Tabebuia	24
2_F_K185n_1_JX297095_Fp_Q5_Nectandra1_OTU102	24
2_F_K185n_12_JX297106_Fp_Q5_Nectandra1_OTU10	24
2_F_K188_2_JX297109_Fp_Q5_Tabebuia27_OTU108	24
2_F_K22_5_JX297103_Fp_Q5_Cedrela19	24
2_F_K23_11_JX2971041_Fp_Q5_Cedrela20	24
2_F_K24_9_JX297108_Fn_Q5_Cedrela21	24
2_F_K262_3_KX108176_F_Q5	24
2_F_K281n_5_KX108184_Fp_Q5_Cedrela	24
2_F_K298n_1_KX108178_Fn_Q5_Cedrela	24
2_F_K298n_5_KX108179_Fn_Q5_Cedrela_OTU105	24
2_F_K315_2_KX108181_Fp_Q5_Tabebuia	24
2_F_K32_6_JX297092_Fn_Q5_Cedrela27	24
2_F_K43_12_JX297105_Fn_Q5_Cedrela33	24
2_F_K78_13_JX297111_Fn_Q5_Cedrela50	24
2_F_K80_2_JX297110_Fp_Q5_Cedrela52_OTU109	24
3_K373_1_Cajanuma1	24
3_K909_1_Cajanuma_Coreplot_unten	24
3_K916_14_Cajanuma_Coreplot_unten	24
3_K944_1_Cajanuma_Coreplot_unten	24
35_K1259_1_Tutupali	24
GQ140623_Archaeospora_VTX4	24
HQ258992_Uncultured_Archaeospora	24
KC708371_Arch_Folioceros_China_VTX5	24
1_K772_1_KX108689_Bombuscarol_03_14_OTU111	25
2_F_K109_11_JX297117_F_Q5_Plant17	25
2_F_K187n_7_JX297118_Fp_Q5_Tabebuia26	25
2_F_K24_6_JX297113_Fn_Q5_Cedrela21	25
2_F_K37_12_JX297114_F_Q5_Plant21	25
2_F_K79_5_JX297116_Fn_Q5_Cedrela51	25
3_K489_4_Cajanuma	25
3_K930_2_Cajanuma_Coreplot_unten	25
3_K914_2_Cajanuma_Coreplot_unten	26
JF414188_Ambispora_VTX283	26
AM114274_Archaeospora_trappei	27
