# Supplementary material for: Species composition of arbuscular mycorrhizal communities changes with elevation in the Andes of South Ecuador
Source: PLoS One. 2019 Aug 16;14(8):e0221091. doi: 10.1371/journal.pone.0221091 (PMC6697372; doi:10.1371/journal.pone.0221091)
Supplement: S4 Table — (PDF) [file pone.0221091.s007.pdf]

S4 Table. Shared OTUs between elevation belts

First Sample	Second Sample	OTUs First Sample	OTUs Second Sample	Shared OTUs observed
1000 m	2000 m	57	66	30 (32%)
1000 m	3000 m	57	37	11 (13%)
1000 m	4000 m	57	32	5 (6%)
2000 m	3000 m	66	37	19 (23%)
2000 m	4000 m	66	32	15 (18%)
3000 m	4000 m	37	32	19 (32%)
