# Supplementary material for: Species composition of arbuscular mycorrhizal communities changes with elevation in the Andes of South Ecuador
Source: PLoS One. 2019 Aug 16;14(8):e0221091. doi: 10.1371/journal.pone.0221091 (PMC6697372; doi:10.1371/journal.pone.0221091)
Supplement: S5 Table — (PDF) [file pone.0221091.s008.pdf]

S5 Table. Similarity indices

First Sample	Second Sample	Jaccard Classic	Sorensen Classic	Chao-Jaccard-Raw Abundance-based	Chao-Jaccard-Est Abundance-based	Chao-Sorensen-Raw Abundance-based	Chao-Sorensen-Est Abundance-based	Morisita-Horn	Bray-Curtis
1000 m	2000 m	0,32	0,49	0,23	0,28	0,38	0,44	0,12	0,24
1000 m	3000 m	0,13	0,23	0,16	0,45	0,27	0,63	0,04	0,05
1000 m	4000 m	0,06	0,112	0,073	0,13	0,136	0,23	0,028	0,024
2000 m	3000 m	0,226	0,369	0,31	0,534	0,473	0,696	0,315	0,251
2000 m	4000 m	0,181	0,306	0,211	0,322	0,348	0,487	0,251	0,191
3000 m	4000 m	0,38	0,551	0,55	0,74	0,71	0,85	0,612	0,497
