# Supplementary material for: Species composition of arbuscular mycorrhizal communities changes with elevation in the Andes of South Ecuador
Source: PLoS One. 2019 Aug 16;14(8):e0221091. doi: 10.1371/journal.pone.0221091 (PMC6697372; doi:10.1371/journal.pone.0221091)
Supplement: S6 Table — (PDF) [file pone.0221091.s009.pdf]

S6 Table. Number of OTUs and number of sequences for the different genera at the elevation belts

(number of OTUs/number of sequences)	1000 m	2000 m	3000 m	4000 m
Glomus	39/441	41/249	15/68	16/80
Claroideoglomus	0/0	2/8	1/4	4/11
Acaulospora	11/40	11/62	16/100	10/88
Diversispora	2/5	1/1	0/0	1/1
Gigaspora	2/5	2/6	0/0	0/0
Scutellospora	0/0	2/3	1/2	0/0
Archaeospora	3/3	7/24	3/6	1/1
Ambispora	0/0	0/0	1/1	0/0
Sum: number of OTUs	57	66	37	32
Sum: number of sequences	494	353	181	181
