# Supplementary material for: Species composition of arbuscular mycorrhizal communities changes with elevation in the Andes of South Ecuador
Source: PLoS One. 2019 Aug 16;14(8):e0221091. doi: 10.1371/journal.pone.0221091 (PMC6697372; doi:10.1371/journal.pone.0221091)
Supplement: S7 Table — (PDF) [file pone.0221091.s010.pdf]

S7 Table. Richness indices

	OTUs observed	Chao 1 abundance data	Chao 2 incidence data	Jack 1
**1000 m**	56	65	65	71
**2000 m**	66	73	77	83
**3000 m**	37	61	49	52
**4000 m**	32	58	48	45
