# Supplementary material for: Species composition of arbuscular mycorrhizal communities changes with elevation in the Andes of South Ecuador
Source: PLoS One. 2019 Aug 16;14(8):e0221091. doi: 10.1371/journal.pone.0221091 (PMC6697372; doi:10.1371/journal.pone.0221091)
Supplement: S8 Table — (A) Frequency of OTUs, divided into 4 classes: number of OTUs in <1% of the samples, in 1–5% of samples, in 5–10% of samples, in ≥10% of samples; (B) OTUs occurring in ≥ 10% of samples of the elevational belt. (PDF) [file pone.0221091.s011.pdf]

S8 Table. Frequencies of OTUs

A Frequencies of OTUs, divided into 4 classes: number of OTUs in <1% of the samples, in 1-5% of samples, in 5-10% of samples, in ≥10% of samples;

B OTUs occurring in ≥ 10% of samples of the elevation belt

**A**	1000m	2000 m	3000 m	4000 m
OTUs <1%	19 (33.3%)	15 (22.7%)	16 (43.2%)	13 (40.6%)
OTUs 1-5%	26 (45.6%)	39 (59.1%)	15 (40.5%)	11 (34.4%)
OTUs 5-10%	7 (12.3%)	9 (13.6%)	3 (8.1%)	7 (21.9%)
OTUs ≥10%	5 (8.8%)	3 (4.5%)	3 (8.1%)	1 (3.1%)

**B**								
	OTU2	10.40%	OTU1	10.50%	OTU43	11%	OTU82	15.50%
	OTU53	11.80%	OTU49	10.50%	OTU80	12%		
	OTU11	17.90%	OTU16	13.30%	OTU87	12.70%		
	OTU22	34%						
	OTU17	39.20%						
