# Supplementary material for: Species composition of arbuscular mycorrhizal communities changes with elevation in the Andes of South Ecuador
Source: PLoS One. 2019 Aug 16;14(8):e0221091. doi: 10.1371/journal.pone.0221091 (PMC6697372; doi:10.1371/journal.pone.0221091)
Supplement: S9 Table — Coloured backgrounds: Lilac: Glomus, green: Acaulospora, blue: Diversispora, red: Gigaspora, brown: Scutellospora, rose: Claroideoglomus, cyan: Archaeospora, light green: Ambispora. The red numbers indicate the most abundant OTUs. (PDF) [file pone.0221091.s012.pdf]

S9 Table. List of OTUs with number of sequences at the elevation levels and closest Blast matches (lilac: „*Glomus*“, green: *Acaulospora*, blue: *Diversispora*, red: *Gigaspora*, brown: *Scutellospora*, rose: *Claroideoglomus*, cyan: *Archaeospora*, light green: *Ambispora*)
The red numbers indicate the most abundant OTUs in this elevation.

OTU	1000 m	2000 m	3000 m	4000 m	MaarjAM Blast: Virtual taxa, similarity
0	2	6		1	**VT112 100%**
1	1	19	5	4	**VT113, VT114, VT115**
2	22	5			VT280 99%
3	3				VT280 99%
4	2				VT92 98,9%
5		5		1	**EU417622 VT92 99,9%**
6			3	1	**HE615060 VT312 99,1%**
7		3			**FJ831530 VT248 99,7%**
10	6				VT84 98,2%
11	38			1	VT126 99%
12	2				VT126 98%
13	11				VT397 99,1%
14		3			**AB555661 VT80 99,4%**
15		3			VT84 99%
16		24			VT84 99,6% Ec DQ336476
17	83		1		VT80 98,5%
18	3				VT100 97,2%
19	3				VT100 97,6%
20	11				VT80 98%
21	11	8			VT79 98,6%
22	72				VT80 98;6%
23				11	**VT74 99,7% 2 Seq.**
24		1			**KF386291 VT72 99,5%**
25		2	1	12	VT88 99,2%
26		2		2	VT223 99%
29	7	7			VT269 Ec
30	4	6			**EU417581 VT76 98,9%**
31		2			**AJ133706 Sclerocystis VT69 99,9%**
32	12				VT111 99,4%
33	2	1			VT235 99,9% Ec
35		1			VT68 98,6%
36		2	1	12	**VT68 99,7%**
38			**1**		**AJ699070 VT73 99,6%**
39			1	2	**HG004504 VT113 99,1%**
40				3	**KY174019** VT74 97,9%
41	1	9	14	12	**JF414190 VT191 99,6%**
42			15		**DQ396749 VT191**
43		10	20	8	**VT191 99,6%**

44	5	8			**VT191 99,4%??**
45		2			VT191 98,9%
46	3				VT191 98,1%
47	3	11			VT124 Ec
48			2	3	**VT123 99,7%**
49		19	1		VT183Ec 100%
50	17				VT292 98,7%
52		11	1		VT182 100% Ec
53	25				**DQ085211 VT292 99,7%**
54	6	1			VT292 98,9%
55		2	1	6	**KC708343 VT315 99,6%**
56		4		1	VT315 99%
57	12				**VT166 99,6% 5 Seq.**
58	1				**AB556928 VT159 99,5%**
59		2			VT423 99,4%
60	2	2	1		**AY903734**; VT423 98,9%
61	10				VT93 98,9%
62	8				VT93 98,5%
63	7	1			VT93 98,5% VT219 98,5%
64	9	4			VT93 98,4% VT219 9845%
65	2	1			VT214 99,4%
66		14			**FJ831546 VT219 99,4%**
67		8			VT53 100% Ec
68	13	16			**GQ140621 VT93 99,5%**
69	2				VT214 98,4%
70	4				VT214 98,5%
71		12			VT179 99,9% Ec
72	8	6			VT122 97,9%
73		2			VT122 97,9%
75	8				VT407 97%
78		1			**DQ085256 VT64 99,9%**
79		3			Glo VT64 99,0%
80	4	11	22		Acau VT12 98,9%
81			3	1	Acau VT12 98,6%
82	4	14	16	28	Acau VT14 99,4%
83	1	1	1		**AJ306440 Acau VT231 99,6%**
84			9	11	**Acau FN 825899 VT230 99,8%**
85			1	1	Acau VT231 97,4%
86			3	6	? Acau VT14 97,8%
87		2	23	16	**Acau KF386272 VT30 99,6%**
88	2	9			Acau VT26 99%
89		1			**Acau VT26 99,5%**
90			3		Acau VT26 98,9%
91		1	7	5	**Acau VT23 99,4%**
92				15	Acau VT23 98,6%
93	6	8	1		Acau VT24 98,5%

94	6	2			Acau VT24 99,1%
95	8	12			**Acau VT24 99,3% A. mellea, A. lacunosa**
96		1			Acau VT24 99,1%
97	1				Acau VT24 98,3%
98			2		Acau VT23 98%
99	2		1		? Acau VT24 97,8%
100	3				? Acau VT24 97,1%
101	3				? Acau VT24 98,6%
102			1	1	Acau VT28 98,9%
103			3	4	? Acau VT28 97,5%
104			4		Acau VT26 97,5%
105	1	1			**AM713428 D. spurca VT54 99,9%**
106				1	**HE615082 Diver VT62 99,6%**
107	4				Diver VT262 98,1%
108	1	1			Giga VT39 99,0%
109	4	5			**AJ852603 Giga VT39 99,7%**
110		1	2		Scutello VT261 98,6%
113		2			Scutello VT52 98,7%
114		5		1	Claroideo VT57 99,5%
115			4	8	**Claroideo VT193 99,8% 3 Seq**
116				1	Claroideo VT57 98,8%
117		3		1	**Claroideo VT56 99,9%**
122	1	6	1		Archaeo VT5 98,9%
124		1			Archaeo VT5 98,2%
125				1	**HQ258992 Archaeo VT5 100%**
126		6	3		Archaeo VT4 98,2%
127		3			Archaeo VT4 97,7%
128	1	3			Archaeo VT4 98,4%
130		3			Archaeo VT4 95%
131	1	2	2		Archaeo VT4 94,4%
133			1		**Ambisp JF414188 VT283 99,4%**
